# Supplementary material for: Bacterial diversity of herbal rhizospheric soils in Ordos desert steppes under different degradation gradients
Source: PeerJ. 2023 Nov 1;11:e16289. doi: 10.7717/peerj.16289 (PMC10625353; doi:10.7717/peerj.16289)
Supplement: Supplemental Information 1 [file peerj-11-16289-s001.zip › 6_taxonomy_community/2_Phylum/All/Phylum_abund_top30_cluster.pdf]

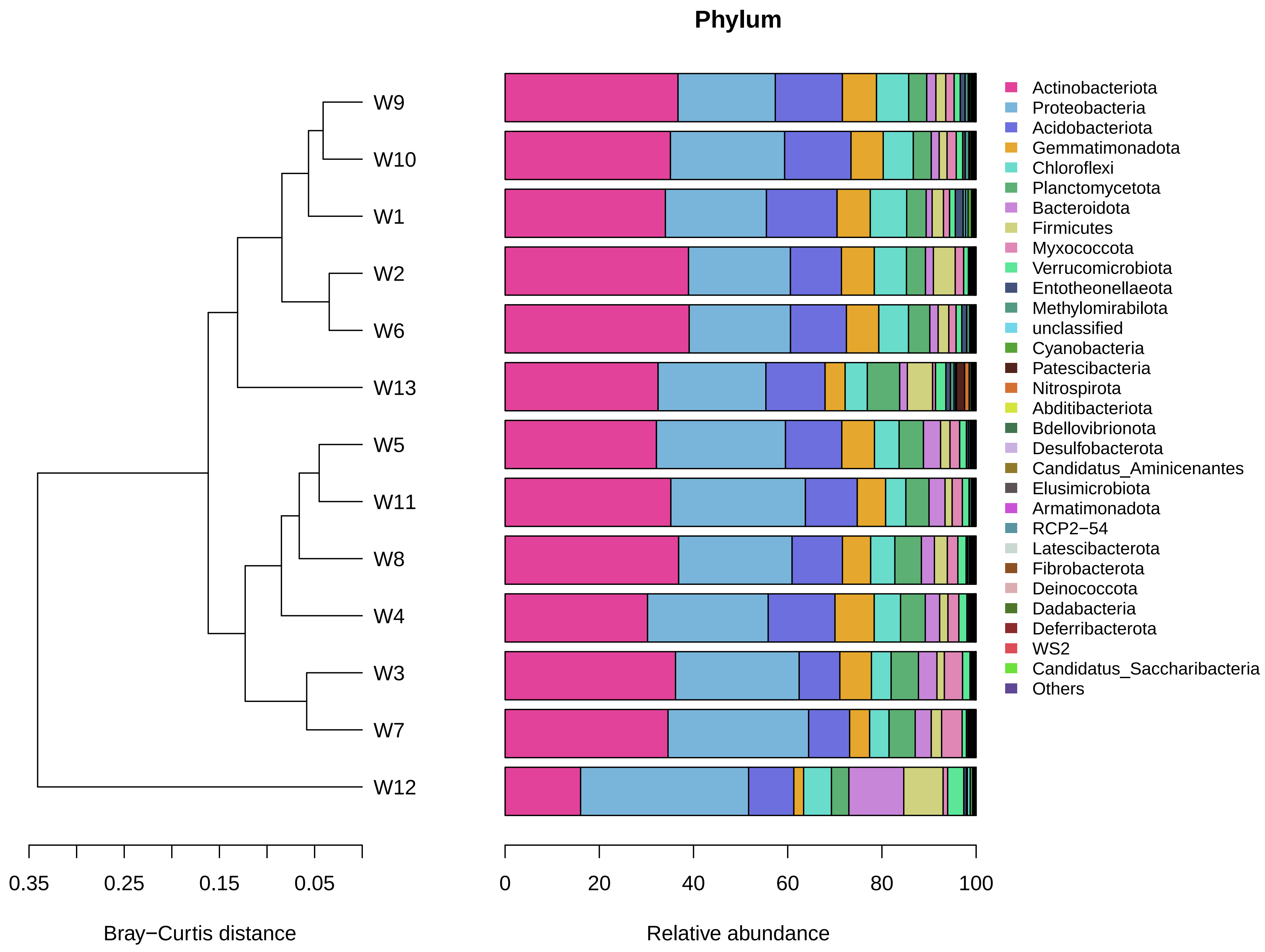

Phylum
W9
W10
W1
W2
W6
W13
W5
W11
W8
W4
W3
W7
W12
0.35
0.25
0.15
0.05
Bray−Curtis distance
0
20
40
60
80
100
Relative abundance
Actinobacteriota
Proteobacteria
Acidobacteriota
Gemmatimonadota
Chloroflexi
Planctomycetota
Bacteroidota
Firmicutes
Myxococcota
Verrucomicrobiota
Entotheonellaeota
Methylomirabilota
unclassified
Cyanobacteria
Patescibacteria
Nitrospirota
Abditibacteriota
Bdellovibrionota
Desulfobacterota
Candidatus_Aminicenantes
Elusimicrobiota
Armatimonadota
RCP2−54
Latescibacterota
Fibrobacterota
Deinococcota
Dadabacteria
Deferribacterota
WS2
Candidatus_Saccharibacteria
Others
