# Supplementary material for: Bacterial diversity of herbal rhizospheric soils in Ordos desert steppes under different degradation gradients
Source: PeerJ. 2023 Nov 1;11:e16289. doi: 10.7717/peerj.16289 (PMC10625353; doi:10.7717/peerj.16289)
Supplement: Supplemental Information 1 [file peerj-11-16289-s001.zip › 8_advanced_analyse/1_taxonomy_analysis/4_Genus_phylotree/genus_phylotree.pdf]

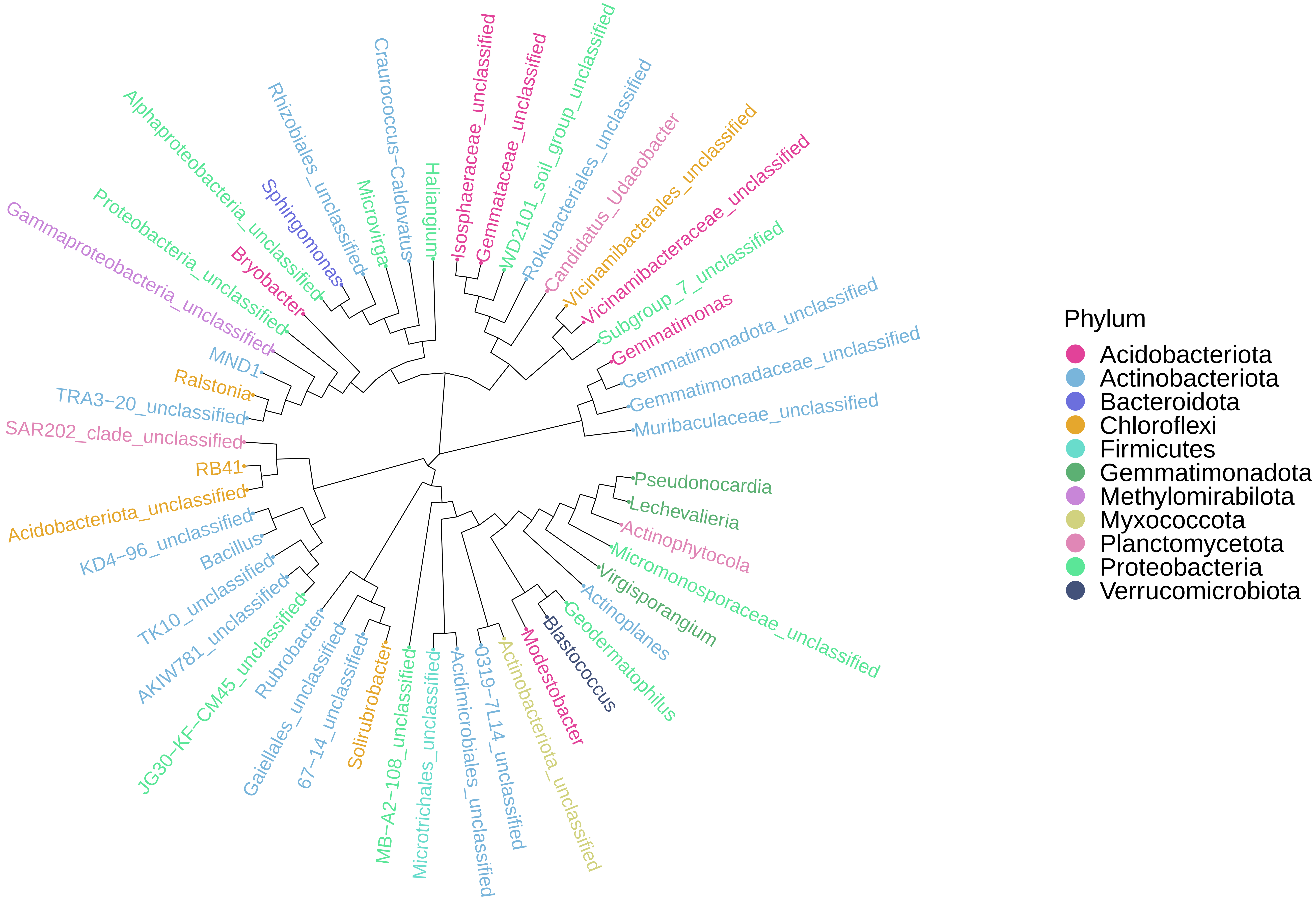

Isosphaeraceae_unclassified
Gemmataceae_unclassified
WD2101_soil_group_unclassified
Rokubacteriales_unclassified
Candidatus_Udaeobacter
Vicinamibacterales_unclassified
Vicinamibacteraceae_unclassified
Subgroup_7_unclassified
Gemmatimonas
Gemmatimonadota_unclassified
Gemmatimonadaceae_unclassified
Muribaculaceae_unclassified
Pseudonocardia
Lechevalieria
Actinophytocola
Micromonosporaceae_unclassified
Virgisporangium
Actinoplanes
Geodermatophilus
Blastococcus
Modestobacter
Actinobacteriota_unclassified
0319−7L14_unclassified
Acidimicrobiales_unclassified
Microtrichales_unclassified
MB−A2−108_unclassified
Solirubrobacter
67−14_unclassified
Gaiellales_unclassified
Rubrobacter
JG30−KF−CM45_unclassified
AKIW781_unclassified
TK10_unclassified
Bacillus
KD4−96_unclassified
Acidobacteriota_unclassified
RB41
SAR202_clade_unclassified
TRA3−20_unclassified
Ralstonia
MND1
Gammaproteobacteria_unclassified
Proteobacteria_unclassified
Bryobacter
Alphaproteobacteria_unclassified
Sphingomonas
Rhizobiales_unclassified
Microvirga
Craurococcus−Caldovatus
Haliangium
Phylum
Acidobacteriota
Actinobacteriota
Bacteroidota
Chloroflexi
Firmicutes
Gemmatimonadota
Methylomirabilota
Myxococcota
Planctomycetota
Proteobacteria
Verrucomicrobiota
