# Supplementary material for: Bacterial diversity of herbal rhizospheric soils in Ordos desert steppes under different degradation gradients
Source: PeerJ. 2023 Nov 1;11:e16289. doi: 10.7717/peerj.16289 (PMC10625353; doi:10.7717/peerj.16289)
Supplement: Supplemental Information 1 [file peerj-11-16289-s001.zip › 8_advanced_analyse/1_taxonomy_analysis/5_Krona.html]

Javascript must be enabled to view this page.

members
magnitude
magnitudeUnassigned

W13
W12
W11
W10
W9
W8
W7
W6
W5
W4
W3
W2
W1

61342591915253054934530204757946561540484653047463531464026246357

19535210410614611454148961004194219

19535210410614611454148961004194219

19535210410614611454148961004194219

19535210410614611454148961004194219

19535210410614611454148961004194219

19535210410614611454148961004194219

19535210410614611454148961004194219

61147588395242654828528744746546507539004643447363531054016846138

2

2

2

2

2

2

121502218316067784826596334

121915285767744826546334

121915285767744826546334

121915285767744826546334

1279184749673812405023

1279184749673812405023

12610101871014141311

12610101871014141311

150

150

150

150

150

333345

333345

333345

333345

333345

635147912189102344109

55352205

55352205

55352205

55352205

55352205

35143771045182425

35143771045182425

35143771045182425

2

2

35143771045182225

35143771045182225

53

53

53

53

53

28422334

28422334

28422334

28422334

28422334

25

25

25

25

25

25

3

3

3

3

3

3

32

32

32

32

32

32

2

2

2

2

2

2

75517

75517

75517

75517

75517

75517

60287099132818512379796256125

60287099132818512379796256125

60287099132818512379796256125

60287099132818512379796256125

60287099132818512379796256125

60287099132818512379796256125

2556162614447107485153844018

402626257637294769246

402626257637294769246

402626257637294769246

322620225334154465174

322620225334154465174

863233143472

863233143472

25561223518223111226151612

175122512927116651410

175122512927116651410

175122512927116651410

175122512927116651410

806110106134161022

806110106134161022

807262222

807262222

6138114168

6138114168

22264222

22264222

22264222

22264222

22264222

22264222

11131635182172887422731223621364

729664510

729664510

729664510

729664510

729664510

1113162016216367539511132621358

10118765913411671421

11732171021

42

42

773171021

773171021

324

3

3

24

24

8794694923

864469317

864469317

15466

15466

318

310

310

8

8

2251347

2251347

2251347

43

43

43

43

111316105878405334312358

111316105878405334312358

111316105878405334312358

111316105878405334312358

357

357

357

357

3614

3614

3614

3614

3614

81591513446

676

676

676

676

289137446

289137446

289137446

289137446

2

2

2

2

12754613316683917162490

12754613316683917162490

12754613316683917162490

12754613316683917162490

12754613316683917162490

12754613316683917162490

140442111315002133211094611457139011162512746121531394787099944

7512805821717129276148613797045682083611359

7512805821717129276148613797045682083611359

7512805821717129276148613797045682083611359

7512805821717129276148613797045682083611359

7512805821717129276148613797045682083611359

366239621099674526285768594976640888491571122464236041

2

2

2

2

2

2

2

2

21180155145249205199197214352145181

177129125235193162197200346119151

36224542312719512137

36224542312719512137

71463343

71463343

5535648063706383944568

2

3588

23131435191917273711

2920453744514656463468

3

310412

310412

38363561564161651163528

9

1311223533303932711928

2519916181122332416

6410512

4136263532173026391615

4136263532173026391615

2

2

21913113309230

21913113309230

21913113309230

710

710

710

31773275614

2

2

3473275612

3473275612

13

13

53522746366102429

52446286102424

52446286102424

52446286102424

35113

35

35

113

113

85

85

85

2

2

2

7

7

7

285174192153395223533651

285174192153395223533651

285174192153395223533651

285174192153395223533651

1272134932172590254125143527251827192565309825082784

182731171714237619

182731171714237619

182731171714237619

24

24

24

21636272960114133341416

84

84

21636272960113333341412

21636272960113333341412

2251101865587108205818270503897

488353

103

48

8

25

18413

18413

1320252222291816203126

1320252222291816203126

131397

131397

28132410

28132410

26

26

42

42

8

8

5

5

1088

1088

8334541226854952

27252117483932

86202052051020

14

14

70730263121206

70730263121206

255410

255410

6

6

6

234

234

234

12

12

12

1412910149162116

14714161010

10

14

7

14168

2

56

56

910911

10

9911

168171271196240145175238155122184113167

10

10

1915039611294060561003327

1915039611294060561003327

16817180136201844619895668480140

1620173431

16817164136201844619875495049140

78133618111614

7101514111310

101514111310

7

3

3

83214

83214

4

4

253114643125375331128

253114643125375331128

253114643125375331128

563

563

563

2529302922

2529302922

5

2529302422

359450675749468549588657730629830911

359450675749468549588657730629830911

359450675749468549588657730629830911

2916551601001006442818695113121124127

3

3

2121825522310632512133575

2121825522310602512133575

3

12711152321

12711152321

137902613111362425683233

521

4663254232

9

4421131152242624

137

483429213071822515352052

483429213071822515352052

8

8

31518108142041791815

31518108142041791815

672514521044756144412108551334108217291001737

128859106175455824431418

128859106175455824431418

3135406659277063717822

3135406659277063717822

372

372

6

6

25199148151161195129199179277204159

25199148151161195129199179277204159

4

4

2422212715234225163630

2422212715234225163630

55917683396783562460725533921487361

55917683396783562460725533921487361

193141139298204164238258401182147

193141139298204164238258401182147

112

112

112

72281328346167584376179229150257443

533262402305564553110480

13

533262402305551553110480

51

51

13917321219159101721

13917321219159101721

82223834

82223834

19425722911522727210616479132363

19425722911522727210616479132363

2510023

2510023

5182576181962437691827213582

2

2

4452574056661818641616191259

4452574056661818641616191259

151925613111917

151925613111917

7366565246

7366565246

11230749

11230749

11230749

6526373830645043472439

3613221620321632

3613221620321632

7

7

292624914441827152439

292624914441827152439

2444926817133123617529330939114572

2443825816132622916628829439113258

7

7

40225815032621716628828138712849

1707961110915693861835718

232179892161261101951952047131

1999

1999

4242

4242

17

17

342

2

34

247

247

1110105795151314

1110105795151314

1110105795151314

8268353631333459467451

8268353631333459467451

8268353631333459467451

8268353631333459467451

5108355

5108355

5108355

5108355

2420557923

2420557923

53

53

242057623

242057623

2

2

2

2

449125340922344156720652355168732783732358417461289

28393129375038

28393129375038

28393129375038

91713

91713

91713

449125340552305151920362355168732413669354617461289

39760151121073876561322611

4

56

34160151121073876561282611

25192222172926601611

25192222172926601611

3442241514175642383035

3442241514175642383035

14323148145183239194200230185167110

14323148145183239194200230185167110

1621329316619992199108112269143108134

1621329316619992199108112269143108134

38510122853183711221566171012882677299929011365969

287181782131575110521071821168621232066924699

6638244392286262614235

485

26363526

19

37917202243561929

9

57301942

5

1921

3327212748

1510

66478325

49

6

16

11

26715

16405456276210231292209426450437258178

43104128859321819

4

14191322

8291214056505318549

311638213016

2

17

111015221618

3231

14

14

251918182945702918

18

5682524181454684228

1046723133454

8237384633257947873419

8237384633257947873419

2812

28

12

28141912

19

10

94

1912

12

12

368338227379297239329259509198224

561175623648

561175623648

561175623648

318289161348253131263210499164149

2036122422132931263923

2036122422132931263923

34

34

295253149324231118234175473125126

295253149324231118234175473125126

454355243946304963467

454355243946304963467

454355243946304963467

456210

456210

456210

456210

3901175636521101411297169919452009738498

3901175636521101411297169919452009738498

515101215

515101215

7617

7617

7582273271943767567121

7582273271943767567121

9661077

9661077

4

4

75491014

75491014

71

71

2504765141171931271462044525

2504765141171931271462044525

2448205574037688345867907431713643458

2448205574037688345867907431713643458

352207219344283377215293266165289160225

352207219344283377215293266165289160225

71162177177249181149207138270126148

71162177177249181149207138270126148

92072014131681913

92072014131681913

28120748147991082013143192177

28120748147991082013143192177

438304287244322182389268232226232197259

438304287244322182389268232226232197259

438304287244322182389268232226232197259

438304287244322182389268232226232197259

269379131144134119113108100154118

269379131144134119113108100154118

269379131144134119113108100154118

269379131144134119113108100154118

2

2

2

2

816756284174343859204378484556327171

615749284166343819204378484552327164

435243143861847310615929223519967

435243143861847310615929223519967

2517547743243998811946234

121391713

25

7223175835157821

90394738186395849923334

20

5710555118

125214718953265024

12

592765532643

72167

8

592011012

150123453338107596885736030

150123453338107596885736030

30712669

30

29

7676

309

201784047

103

103

98784047

98784047

3982638372449831420142551

3982638372449831420142551

42

42

3982634352449831420142551

3982634352449831420142551

617379351814306335446387657170658192164

8

8

8

8

12313116

6

6

6

1231311

1231311

1231311

597594

597594

597594

597594

4942631362082121791221471081036424112

4942631362082121791221471081036424112

4942631362082121791221471081036424112

4942631362082121791221471081036424112

116210593941473242225416258515852

6724373615654994256

7

7

1021127221025

25

10211272210

24

24

67272424918796

67272424918796

12

12

1265

1265

9

9

1445301684234743304529670

10170705245150

10170705245150

564291614148632785270

162663

55

39

1311427

1718854

56204105635216

88161170

70

11

16

88

24

24

492017232311821017913512

492017232311821017913512

492017232311821017913512

4

4

4

229152514171071735334

229152514171071735334

15251417131214

12

9

2282524934

4345549817411018054811822026829190105

2

2

2

2

19680233233513810443

19680233233513810443

19680233233513810443

19680233233513810443

4325941814978147497802102682879062

15573507417845941101164223

15573507417845941101164223

15573507417845941101164223

313

313

313

25511

25511

5

11

25

21542226202961939

152629911

116

11

9

4229

2114324

2114324

2522202924

2229

52024

20

2225920954267328835931249728

382714352729

3827352729

14

221261043232554952

22

12643205554952

27

10

1717263026383428

1717263026383428

25928161416

25928161416

89711603725753160295324962924310122811990156616432275

421

421

421

421

2

2

2

2

3139361135542221984466305570

3139361135542221984466305570

2

2

22

22

2

2

3137161135522201984466305570

3137161135522201984466305570

2053451071562965245

2053451071562965245

2053451071562965245

2053451071562965245

371639106509358297134414146123235158

371639106509358297134414146123235158

371639106509358297134414146123235158

371639106509358297134414146123235158

19213216113046414315499183125109

19213216113046414315499183125109

4581684140884658344151

4581684140884658344151

14751938942455108411498458

14751938942455108411498458

34725151593120371514

34725151593120371514

34725151593120371514

34725151593120371514

5

5

5

5

552529231193187276197308271251241218236

346

346

346

47220590718611355120130107958896

47220590718611355120130107958896

26

16

446247596555797365666571

37528242732415742292325

27

134

71

8032414112210116395188141138146130140

8032414112210116395188141138146130140

24

203045313029504343373041

122

6080777012366114989510910099

202

31

10

13

13

13

30124244232

30124244232

942443

942443

30322

30322

71756124121682833443

71756124121682833443

71146105110667827417

71146105110667827417

610191115626

610191115626

2401829291654341921191734

2401829291654341921191734

2401829291654341921191734

2401829291654341921191734

70269625147817371534129711381472126110567798991302

4521613010351714436

4521613010351714436

4521613010351714436

2931222457214197301269298456229238191185

447171527287420

447171527287420

190115826352271221318124856903544

190115826352271221318124856903544

741111251091399214513813410711884

741111251091399214513813410711884

64

64

293930444031454232413437

293930444031454232413437

27594377105636410410896108871037744

396446616292740268

396446616292740268

3

3

4

4

534839344434664646533229

534839344434664646533229

2706437315242416814331410197

2706437315242416814331410197

1744135126866359442221651822423672186427

634719

634719

1991448111116

1991448111116

1557135121658954937213746417919529146369

1557135121658954937213746417919529146369

5

5

1872859314771393430274039

1872859314771393430274039

332332312542192836282922

332332312542192836282922

332332312542192836282922

812108467

812108467

812108467

14077328566436270269378266352207315400

14077328566436270269378266352207315400

14077328566436270269378266352207315400

42

42

42

55510

55510

55510

185

185

185

2029132028165120189666741946587

8

8

12372409978605541834065

12372409978605541834065

19974666

19974666

64

64

37

37

39815

39815

1721121111

1721121111

167

167

731067

731067

4622224496222428302343

222445222126252337

222445222126252337

462463256

462463256

166011811249121378

166011811249121378

166011811249121378

119231951391915372429953

119231951391915372429953

119231951391915372429953

48125

48125

48125

2

4812

3

9824209152116157454152222174131144184

40913932521526161522

35

3

5

1711

1711

1016107

1016107

35

35

910927151610

1015

99111610

16

23

23

365

5

36

2

2

5824200139107125402137222148115129162

152311

8

148

3

3

4

67

67

439

431

8

1917189

1917189

582491102941056911911612010698107

3

24

5891102941056911911312010698107

1474

1474

2

2

702013911514103173155

710

3514791081110310318

7

3566337

42

42

42

42

170463218017312410288208120141113133118

4

4

4

7718772614

77

77

18772614

77

82614

10

30562

2

2

2280

2280

776

776

91716224

91716224

91716224

8415761531399810286122116115113119114

47

47

211211819

211211819

10

10

15559462471221165049

155511

9462471111165049

84669855781155310065

84669855781155310065

1049

1049

1049

4

4

4

4

4

2511111410948

2511111410948

2511111410948

2511111410948

2511111410948

2511111410948

511345553237

511345553237

511345553237

511345553237

511345553237

511345553237

1992394891848719281194651753016105211221495114354192281567815774

12656488271158117985680612659399186839521671

438971031061771079615163591287105

438971031061771079615163591287105

438971031061771079615163591287105

438971031061771079615163591287105

218301221498482222131323250261152225299

169188197482401206118316245258152208270

169188197482401206118316245258152208270

169188197482401206118316245258152208270

4911346241173108

2346134734

2346134734

26113

26113

4

4

11710

11710

10352

10352

10352

2022165721

2022165721

1122165721

9

34412219512013170193156122124125158

34412219512013170193156122124125158

34412219512013170193156122124125158

34412219512013170193156122124125158

7233

7233

7233

7233

2582503813574003965095984704763955121106

23511114314416017715914813415198174

314

314

23561936084105875352606578

23561936084105875352606578

4750847658729582913396

4750847658729582913396

281361512121121293

281361512121121293

281361512121121293

212138516109372118

212138516109372118

212138516109372118

25813230201250208235411301333186364911

25813230201250208235411301333186364911

25813230201250208235411301333186364911

225567

225567

25

25

25

567

567

567

2

2

2

1711632483713

1711632483713

1711632483713

1711632483713

1711632483713

88649628447048424411843022722637180430

88649628447048424411843022722637180430

88649628447048424411843022722637180430

88649628447048424411843022722637180430

88649628447048424411843022722637180430

24814864713711338104448710687795306166881197

74294250

74294250

74294250

74294250

11

11

11

11

17414864713421296104448710077795306166881197

1741486471337126710444669797695306166591177

1741486471337126710444669797695306166591177

1741486471337126710444669797695306166591177

529212011

529212011

529212011

810299

92

92

810207

810207

8480399224355247465226391234481220492336148821613789

32578223412316610

32578223412316610

32578223412316610

32578223412316610

513121121650326928371756940318515261783125116302366

17597518491449120692256916208128667158991085

6459926487877213056137107

6459926487877213056137107

139774057310779236603611203567563445526836

33

2511072104183601874744018919519160323

3

975633354644519424257725378353346366482

22916727

1491544302431155331

11011148191151147103223149139191200109

11011148191151147103223149139191200109

252641224051181072434233633

252641224051181072434233633

107680

107680

107680

316713447471788150681435914576817955367051173

316713447471788150681435914576817955367051173

316713447471788150681435914576817955367051173

2

2

2

205172932103131210231122695

205172932103131210231122695

205172932103131210231122695

15223013

15223013

15223013

302418737771956176887129115965235532375141413

252413707221710151476727513715064921954241240

252413707221710151476727513715064921954241240

252413707221710151476727513715064921954241240

500503552462541041622517614290173

500503552462541041622517614290173

500503552462541041622517614290173

131511

131511

131511

131511

5544186880585497650768388554805862025649910769254626

210209333124202012

210209333124202012

10209333124202012

10209333124202012

2

2

73825516719622540623826125839120095

73825516719622540623826125839120095

18415016319835119521024937620091

18415016319835119521024937620091

86269

86269

71171919482319154

71171919482319154

738147146

738147146

832026251695181129662618178827742041386621731179

202332140915012540226114332368167733201812927

596254260678595294574433922401146

596254260678595294574433922401146

2062349765210016175839776121324713426

2062349765210016175839776121324713426

111365858986110495568176321074698355

111365858986110495568176321074698355

5611

5611

5611

112

112

112

2249701194245529559

2249701194245529559

2249701194245529559

836013712160497268424010896

4232251811719

4232251811719

8360958935495457423310877

8360958935495457423310877

781327721191817191135716

781327721191817191135716

781327721191817191135716

13313614624511914027925833010179

13313614624511914027925833010179

13313614624511914027925833010179

922989293735584791267559561511861819437

922989293735584791267559561511861819437

38301446364955758238

38301446364955758238

140313939113454589

140313939113454589

269131691591016

269131691591016

2913324242281877558118

2913324242281877558118

1413102014

1413102014

128213510740182511054

128213510740182511054

6091358977225833810154

6091358977225833810154

11261930381854267334

11261930381854267334

435842533592450

435842533592450

3229262147211225446186224105486370149

3229262147211225446186224105486370149

2817633146644466348966

2817633146644466348966

589269017416613329811710617921233544

1910

10

10

19

19

582311470811514939

23

23

9

9

2039

2039

283281514

283281514

30627073

30627073

5216497689313088931221817336

5216486689313088931111817336

5216486689313088931111817336

1111

1111

352452958168

352452958168

352452958168

2

2

2

3571392624198203

3571392624198203

3571392624198203

1725119941178

14

14

17

17

1141178

1141178

25

25

85

85

121786258

121786258

121786258

121786258

721234469815221543

14264

14264

14264

772322271522123

7323272133

7323272133

4141312

4141312

9

9

8

8

222853

3

3

22285

22285

341211642471306121611113885148243

341211642471306121611113885148243

341211642471306121611113885148243

341211642471306121611113885148243

321271016023147249154269295459

321271016023147249154269295459

32127101602314724113952295459

32127101602314724113952295459

815217

815217

664257489477490523644519516398494451327

612257442439457480632475469365477416295

2

2

1046481127110861558618415640

1046481127110861558618415640

5041818

5041818

4863115412713412618117312912310311790

4863115412713412618117312912310311790

108226148193180222263177165139186137137

108226148193180222263177165139186137137

182776988

182776988

95145931129468

95145931129468

524738334312444733173532

52272933212219152232

52272933212219152232

54

54

2092212222318179

2092212222318179

254488329372061342166638250372157557447

254488329372061342166638250372157557447

254488329372061342166638250372157557447

254488329372061342166638250372157557447

12254

12254

12254

12254

163925124221595209018142720360215361603292420991731

163925124221595209018142720360215361603292420991731

13771251139

13771251139

18368512127951044904146014316659152

18368512127951044904146014316659152

633228588832614810756

633228588832614810756

8531799337109969451086144884176916201129925

8531799337109969451086144884176916201129925

8

8

55443315084762984351062313399715333363

55443315084762984351062313399715333363

498994288301169133122143187119215

498994288301169133122143187119215

3007332362043855464215

3007332362043855464215

4644207578629873464436153

4644207578629873464436153

11192051117

11192051117

8395528476168893934496041

12545

75

75

545

545

41349

41349

41349

8380528464150813929406041

8380528464150813929406041

8380528464150813929406041

11

11

11

5

2

2

2

2

3

3

3

3

3500233762195527529859034872548047554682725447654048

19

19

19

19

208831392452221731292859316752366182

20883116216222127912729316752156182

20883116216222127912729316752156182

20883116216222127912729316752156182

2329463813210

2329463813210

2329463813210

2364188422472679281025231503269022141904261718112406

2364188422472679281025231503269022141904261718112406

2364188422472679281025231503269022141904261718112406

2364188422472679281025231503269022141904261718112406

4138241630205112033

94

4

4

9

9

19

19

19

413824163020429733

413824163020429733

413824163020429733

4472142726182014022329246816331705190835511778895

4472142726182014022329246816331705190835511778895

79

79

9414

94

14

861475986341106815115468509311952930233

17

869806662598239133136267231581750155

20

3749

1524303916205938

14314165126771081181171851954

343214

1924

166547244567547197104

33

19

16

2

2

171419

9844

93514382070467025

20

55

2036

935141834461525

32411140230371235213184180370157148

3611189933019310212710830289

36

32502295414211157726868148

64

64

62485694955281861647646

62485694955281861647646

1159119913922131146211

1159119913922131146211

24273720312592

20

242737312592

2057151824

6

20151824

51

31228231327480253178380176244177151250

31228231327480253178380176244177151250

1237170708410510315112940

26

977170708410510315112940

11261138

1111

2638

33327221216261527

33327221216261527

1002218411416814615495155401232126

4530

55464049131

2923

36

42292614

36

5419253111

18

31

41

22

40

5816603543594466448972

40

923

7751134107

46

246156498219183264301183232158181166150

17141815

18

18

1714

1714

15

15

24615628910510214191103115796978130

67

67

2461562221059914191103115796978130

11

4452124737452039913

33

41

21

84

187106448094595670593036102

5972971015

31

3

3

78221210

78221210

7

8221210

4

4

4

20990591051958095671127420

37

37

413

413

2915

2915

21193322202435

21193322202435

3519

3519

2327

2327

472

11

36

2

99523433507067412

99523433507067412

5

5

52133845303218

52133845303218

2

2

2

2

9583240311644427

6762015

6762015

6762015

9523233251642412

9523233251642412

8

9433316227

325

24

9224

10

10

10

10

7995

7995

7995

95

79

92551138

92551138

92551138

255

91138

251150290120110235871251656462

251150290120110235871251656462

351935

351935

82161761019

82161019

176

158117231758711355811176158

8011417

1583711758879155811176158

22

8647054

8647054

2

2

999

9

99

3

3

27133384413

13813

2733

44

5

5

5

5

235308369299437282375394348684398255

235308369299437282375394348684398255

187121172236115170204127319130155

1271111818

150121139225104162201127276130103

2

2

3

23242552

22875454567

2

22875454367

362641433327555069

362641433327555069

785222111138917813517129626833

497233

85731138985110110189117

25

8611256110771

7

2

142153

13492023748757688837429653659817845409563

623

623

6

6

6

23

23

23

1479116104325

1479116104325

1479116104325

1479116104325

1479116104325

2165

2165

2165

25

25

16

16

12021112665615616653400584567665589347553

3463395514285757982965960

3460395513866717478945960

3460395513866717478945960

3460395513866717478945960

34144542

4

4

7

7

347542

347542

5

5

5

23

23

23

23

709828592550512452243479452558466254481

709679592550512452241479452558466254481

53615634732433626984340284367256179356

53615634732433626984340284367256179356

17352324522617618315713916819121075125

17352324522617618315713916819121075125

2

2

2

149

149

149

301101339374827202517246

301101339374827202517246

172153999

172153999

7

7

2848133922452741617156

2848133922452741617156

158284111022241610106

158284111022241610106

15828411101924141010

15828411101924141010

326

326

2

2

2

2

2

831366618217469014925462

831366618217469014925462

831366618217469014925462

831366618217469014925462

831366618217469014925462

119261414

119261414

119261414

119261414

119261414

119261414

3

3

3

3

3

3

7703568257817742755350884050641755636733459443636952

723234367819434649347398287377318441363

723234367814431649347398287377318441363

723234367814431649347398287377318441363

723234367814431649347398287377318441363

723234367814431649347398287377318441363

53

53

53

53

194571086298143413104

1945710057754188

243

23

23

4

4

194579853754185

194579853754185

194579853754185

859643316

859643316

859643316

859643316

1812176034553299367121971140283427333684230022823534

4264

4264

4264

4264

27175181610242454129

27175181610242454129

27175181610242454129

27175181610242454129

181217603063309334611940950260823923222163820443364

181217603063309334611940950260823923222163820443364

181217603063309334611940950260823923222163820443364

181217603063309334611940950260823923222163820443364

1556101163179102

1556101163179102

1556101163179102

1556101163179102

1571491222522145

1571491222522145

1571491222522145

1571491222522145

34616513522015115131739658619750

34616513522015115131739658619750

1268586106485918018327612338

1158586106485918016826712338

11159

17138278980619116122628

56132919175383

11538277651427410814328

64710209

64710209

3838172523153632757

3838172523153632757

5593912

539

912

5

2

2

2

2

2

233893270257235289333243256263374191255

21616178

21616178

21616178

21616178

47998817611232512

8

8

8

56763

3

3

5676

5676

47948171116199

47948171116199

47948171116199

229891263232210281325209242252351166243

229891263232210281325209242252351166243

229891263232210281325209242252351166243

229891263232210281325209242252351166243

9724

9724

9724

9724

9724

6

6

6

6

6

4142236912442615257114731719243317601885100612302151

380284311714159351386562163377

380284311714159351386562163377

380284311714159351386562163377

380284311714159351386562163377

376223411201249824301414168422951695182399011972074

272111727611483162191711651509105510046307591342

16127174104933

16127174104933

2669105168314201500879108813749979506097191257

2669105168314201500879108813749979506097191257

52121625112131601314850123782

52121625112131601314850123782

104111694401015809497519786640819360438732

104111694401015809497519786640819360438732

104111694401015809497519786640819360438732

269265277494430380214409409378397151461

269265277494430380214409409378397151461

269265277494430380214409409378397151461

269265277494430380214409409378397151461

269265277494430380214409409378397151461

140135221552

140135221552

140135221552

140135221552

140135221552

106192114671121

106192114671121

106192114671121

106192114671121

106192114671121

7510610310769209781151461566559

7510610310769209781151461566559

7510610310769209781151461566559

7510610310769209781151461566559

7510610310769209781151461566559

53624

53624

53624

53624

53624

39116

39116

39116

39116

39116

4811192154253551721233491681423797264

4811192154253551721233491681423797264

4811192154253551721233491681423797264

4811192154253551721233491681423797264

4811192154253551721233491681423797264

4811192154253551721233491681423797264

8543

8543

8543

8543

8543

8543

99468971786926103013141587958169014532087676587

4326232028272236392924

4326232028272236392924

4326232028272236392924

4326232028272236392924

4326232028272236392924

971938166

971938166

71916

4

4

71912

71912

3

3

3

6386

6

6

386

386

6443171155977184811381392783152812161779568488

992227175158151289217193212144171144168

13263

13263

3

3

2

106

191581446

191581446

2

131381446

6

732227128132125258187157186130133127141

1299237

27

12993

612227128127125226178145177130133120134

612227128127125226178145177130133120134

107

107

5236

5236

562

562

562

417410926302410251027

36

36

9511108

9511108

7883

7883

34

34

610822526

610822526

44651510

44651510

8

8

32

32

32

32913445

335

335

244

244

2845

2845

127265730351470364552391683598890241249

60265723351470359549391683596890239249

1774488137284610689148739

1774488137284610689148739

2726363644194345461510

2726363644194345461510

1042014959213949541910

1042014959213949541910

785258624658871201752516

785258624658871201752516

76477124

76477124

128115847511

128115847511

52645222448

52645222448

4347167421836237111114422

4347167421836237111114422

66

66

6326

6326

4545454626365559473645

4545454626365559473645

19210811911592671951842576855

19210811911592671951842576855

176368422

176368422

1347

1347

715030282952403332311829

715030282952403332311829

67

67

67

75322

75322

75322

53329081327

3

3

3

427

427

427

4628669

4628669

4628669

4324

4324

4324

22492210919476726

224911538108

224911538108

224911538108

5837418

16

16

8218

8218

5194

5194

47

47

47

323

323

323

422

422

422

2

2

2

19467057924921646648619361846169218164

18824290131225951184072592658626

44223116172523296

44223116172523296

1293382756933201713120

1293382756933201713120

178116

178116

170747315369851811542056020

170747315369851811542056020

15

15

74

74

74

824716645619485313731

9115101518

9115101518

71291659539483010931

71291659539483010931

273810

273810

14114974321347936991011973228

1317422126502786771773219

1317422126502786771773219

7108591317207

7108591317207

3

3

14182472

14182472

323101448

323101448

323101448

1941893383043242206048793210

5618411336783113397

5618411336783113397

13852253071641835402510

13852253071641835402510

29

29

2

2

32

32

32

257430

257430

257430

257430

257430

35024132317721334161417

35024132317721334161417

35024132317721334161417

2172

2172

350223

350223

15132315521417

15132015521417

3

7332456

7332456

7157788056607161913337

7157788056607161913337

7157788056607161913337

7157788056607161913337

7157788056607161913337

2982839344985301752192315

298143427492426833131015

298143427492426833131015

298143427492426833131015

298143427492426833131015

1457614919613

69562

69562

69562

14575541411

54

54

1457501411

5711

5014

14

3428

3428

3428

2975

2975

453

453

2713171019141917603

2713171019141917603

2713171019141917603

44

44

2713171019101917563

2713171019101917563

914141099322

914141099322

914141099322

914141099322

914141099322

914141099322

10613166785248152482922493438

2

2

2

2

2

234232

234232

234232

234232

234232

2

2

2

2

2

10483156543642134292520402115

10483156543642134292520402115

404275

404275

404275

3142284935

312243

312243

4895

4895

3216101194317131816

3216101194317131816

3216101194317131816

1008241821221811471325

1008241821221811471325

1008241821221811471325

1653

1653

1653

13481321017224721

22

22

22

22

24

24

24

24

2

2

2

2

13392142229

13392142229

13392142229

13392142229

244

244

244

244

438343

438343

438343

438343

2

2

2

2

2

49322222

7

7

7

7

3

3

3

3

422222

422222

422222

422222

2

2

2

2

42

42

42

42

42

4222218825842098202826782586243824032488308316241918

1111128446424410223218

1111128446424410223218

1111128446424410223218

1111128446424410223218

1111128446424410223218

3059204688877597812931307103094582011166471092

65684511

65684511

65684511

65684511

23211743254303426328298353202192146165484

23211743254303426328298353202192146165484

177761426431616261622171133

177761426431616261622171133

1872154016923731021511928511814737113342

1872154016923731021511928511814737113342

112162317551911411531

112162317551911411531

4

4

26112750345080108424912512674

26112750345080108424912512674

289544010333413726433552369

9873129152

9873129152

9873129152

2895431233346426312637367

2895431233346426312637367

2895431233346426312637367

3432491341172061182521661311678357178

3432491341172061182521661311678357178

102333763806571373879332357

102333763806571373879332357

17325372380362526192553

17325372380362526192553

2242166549893010193686231968

2242166549893010193686231968

106454340307813612481564426835389410

106454340307813612481564426835389410

711141012

711141012

912824125923086629616

912824125923086629616

106356301283688506441478364739389382

106356301283688506441478364739389382

34489115

34489115

34489115

34489115

34489115

48681120141956615211124

486811201419566151124

16

16

16

1136

736

736

4

4

48201415536151113

48201415536151113

48201415536151113

5245

5

5

524

4

52

21

21

21

21

95

95

95

95

95

159122971873

159122971873

159122971873

159122971873

159122971873

8457416711263950136812101290143316241932964771

18242713201217156108

18242713201217156108

271352472

271352472

111711466178688

111711466178688

52

52

32122

32122

8417416531239921135511901278141616091926950763

932

932

932

4610188132521212

2738485

2738485

19101591716212

19101591716212

8417416071229921133711731265138815881905948761

8417416071229921133711731265138815881905948761

8417416071229921133711731265138815881905948761

4

4

4

4

24

24

24

24

7611912512326881191131246272

7611912512326881191131246272

7611912512326881191131246272

7611912512326881191131246272

7611912512326881191131246272

7611912512326881191131246272

590333592935461641545682001625796764

590333592935461641545682001625796764

590333592935461641545682001625796764

590333592935461641545682001625796764

28712225178209102783449296

28712225178209102783449296

590333312223241137635998842347468

590333312223241137635998842347468

3

3

3

3

3

3

7252438942

7252438942

7252438942

7252438942

7252438942

7252438942

32894943793917111013091027122492983583818651116

295832694725938357366706694544572951450656

8433

8433

8433

8433

8687253218975842

8687253218975842

1751113759

1751113759

693714191197833

693714191197833

4255273

554

554

554

42233

23

23

423

423

19114011621192771811954246510063102148

11

11

11

14676332386394

28

28

1468341

1468341

258615

258615

51

23

17

11

331510

331510

1724

1724

1724

176540186108227158187424591006090348

1122

11

22

17653907110822715517742259986073148

1317

22

222

19

152163365839727516020573497

8

1337264262132

7

1067282837143578920931412654916

150

20

94

74

11

235

25

2890

13

153822170

211

25

15382109

25

289903266655352271720125744

684

684

684

283063266655352271720125744

12411476

12411476

16326261424723820125536

16326261424723820125536

22

22

4

4

3062

3062

5

5

11

11

11

11

1511836271367448480418209358332220364516

1311820233333417464377184320310197324484

18463632269185783952

18463632269185783952

4718137268165841163999535983167

4718137268165841163999535983167

2

2

23

23

827143219216345235136203198130202265

27262092382420212114

8

807136193196253197112175177130181251

1693

1693

9

163

56

56

56

203834311632253316204032

203834311632253316204032

612

1713

202117181615192116202332

171717

17920765610801042949588861

17920765610801042949588861

17920765610801042949588861

17920765610801042949588861

17920765610801042949588861

196162416201740

196162216171737

196162216151737

4

4

196162216151733

196162216151733

2

2

2

233

233

233

233

1521674301248219544271435422313465310359

481223

481223

481223

481223

1251011122424

1251011122424

1251011122424

1251011122424

22

22

22

22

61171324481313926191012

4242

42

42

24

24

61

61

61

17132046131352619812

131384213135211588

131384213135211588

7454

7454

454

454

90128581899720414021819514717391154

545135765

545135765

545135765

4

4

4

20443238110961058570962481

10121131210

10121131210

692216382415918

692216382415918

104711

104711

143532283737593638572453

143532283737593638572453

2422217

2422217

1587

1587

701285374859903910010570716273

13143581429452225441

13143581429452225441

34

34

2

2

13149111047

13149111047

2

2

108

108

1612852339165811623432354115

1612852339165811623432354115

7981113479

7981113479

2555513

2555513

2555513

2555513

14726436106193136501144768

2312

2312

12

23

42

42

42

851175695

8594565

8594565

239

239

2

2

2

123036689513302745

123036689513302745

123036689513302745

14522921821263184114

4

4

13

13

6454

6454

4

4

7

7

145229102212231676

145229102212231676

4

4

4

4

4632810477571638216016978135136122

4632810477571638216016978135136122

5

5

2

2

2

2

3

3

106

106

5519

5519

4432810271571487915015478110131108

4432810271571487915015478110131108

2

2

6101039

6101039

381575113610699441060202583395910982052723602

677154910

677154910

677154910

677154910

677154910

260121108160861194891703808471

260121108160861194891703808471

765

765

765

260114102160861144891703807771

91465644482464443162964

91465644482464443162964

260961919431021820

260961919431021820

4

4

122414914

122414914

83354211621458

83354211621458

6572765227

6572765227

7

7

7

3812481015954769970190678585910281672639521

3

3

3

3

197131318479267306334360212369437173362

197131318479267306334360212369437173362

197131318479267306334360212369437173362

197131318479267306334360212369437173362

1674514106122476

1674514106122476

1674514106122476

1674514106122476

3198229024213337612

3198229024213337612

2

2

2998229024213337612

2998229024213337612

26

26

26

26

533

533

533

533

16811765645847562314703956136051156439139

12294342103281331541604061596

12294342073261331541604061596

12294342073261331541604061596

32

32

10938226321318125112635431542816385

298

298

536448527331140121623224

536448527331140121623224

591468342107351201061788729

591468342107351201061788729

230435367523868551492924

230435367523868551492924

21

21

109111029201914263318158

109111029201914263318158

168905419214180812063901657844

168905419214180812063901657844

168905419214180812063901657844

4911

4911

4911

86247329174164240146394

86247329174164240146394

86247329174164240146394

3475276188

3475276188

3475276188

3475276188

2

2

2

2

109

109

109

109

109

109

78176214301162224

22

22

22

22

22

401214622

401214622

401214622

401214622

401214622

17624422

176442

176442

176442

176442

22

22

22

22

382125

382125

382125

382125

382125

2886349922543507362724441916341424372655222227523583

8122173311

8122173311

8122173311

8122173311

8122173311

1981301441742028713317414615591143333

1981301441742028713317414615591143333

1981301441742028713317414615591143333

1981301441742028713317414615591143333

1981301441742028713317414615591143333

76533336354356919412152527518457226532

1812692913894401466040622812932154428

1812692913894401466040622812932154428

1812692913894401466040622812932154428

1812692913894401466040622812932154428

5846472154129486111947552572104

5846472154129486111947552572104

5846472154129486111947552572104

5846472154129486111947552572104

2893571005143914411302960133411381317134713981407

5320461188585876564587671283371111281019

10652274

10652274

10652274

22115481817534328

22115481817534328

22115481817534328

532046098648337556378536938287061078987

532046098648337556378536938287061078987

532046098648337556378536938287061078987

200153169206246151189248126156173110123

342413394520232165184

64

64

2

2

342411393920232165144

342411393920232165144

73396615197552

73396615197552

73396615197552

2001531281492241061382138412810387117

2124582651372356421337

1025191115

2114332651182345421322

148222374

148222374

5215310710314380871696172577480

5215310710314380871696172577480

36225348337386126210300328460160265

36225348337386126210300328460160265

36225348337386126210300328460160265

36225348337386126210300328460160265

3

3

3

3

17551016155119475050807947

1914510603688157

1914510603688157

1914510603688157

1914510603688157

252010293521341456449

252010293521341456449

17202019151431219

17202019151431219

810916620142523

810916620142523

322

322

322

322

1711224016242372314188

725913141418

725913141418

725913141418

174203571123798

174203571123798

174203571123798

3322

3322

2

2

32

3

2

3

3

45321

45321

45321

45321

108800124180265133862899217967182169

108800124180265133862899217967182169

108800124180265133862899217967182169

108800124180265133862899217967182169

108800124180265133862899217967182169

1011773

1011773

1011773

1011773

1011773

220132895425778145463834

220132895425778145463834

220132895425778145463834

220132895425778145463834

220132895425778145463834

6242

6242

6242

6242

2

642

2

2

2

2

2

4027376373

4027376373

4027376373

4027376373

4027376373

395511315590457331259483340337294350637

395511315590457331259483340337294350637

395511315590457331259483340337294350637

395511315590457331259483340337294350637

395511315590457331259483340337294350637

9028371023866143106507985

9028371023866143106477985

9028371023866143106477985

9028371023866143106477985

9028371023866143106477985

3

3

3

3

24

24

24

24

24

1001858199386410253208404265279187252325

1001858199386410253208404265279187252325

1001858199386410253208404265279187252325

1001858199386410253208404265279187252325

1001858199386410253208404265279187252325

14816184101652955

14816184101652955

14816184101652955

8611134251935

8611134251935

8611134251935

6255816102

6556162

6556162

2210

2210

2612123131713752384328531979371732273953356528073269

37723718316326212513616410216297111160

37723718316326212513616410216297111160

37723718316326212513616410216297111160

37723718316326212513616410216297111160

37723718316326212513616410216297111160

26141929191951227

26141929191951227

26141929191951227

26141929191951227

26141929191951227

19475321326496270470687711952782296

19475321326496270470687711952782296

19475321326496270470687711952782296

19280196151327106160327349288218151

19280196151327106160327349288218151

3560223878445884107145

21212

1460223666445884107145

1606515313186266302278557419145

1606515313186266302278557419145

20968445714568378188499345495284302570

20968445714568378188499345495284302570

4486373714221855155

4486373714221855155

4486373714221855155

20968401706562341151485323477229287565

328354793127

328354793127

16302313192631

16302313192631

20968353668562283104463304420171287565

20968353668562283104463304420171287565

4774250299304232129323374373281193327

4774250299304232129323374373281193327

4774250299304232129323374373281193327

4774250299304232129323374373281193327

4774250299304232129323374373281193327

166578417362144228015731251214116442139194014031789

166578417362144228015731251214116442139194014031789

166578417362144228015731251214116442139194014031789

56373027467076648877

56373027467076648877

5267137941821891302663014729421

5267137941821891302663014729421

160378498016311891806542170869510953607181479

160378498016311891806542170869510953607181479

574333392655584743036136671044503212

574333392655584743036136671044503212

2696868927430510170611116100

2696868927430510170611116100

2696868927430510170611116100

2696868927430510170611116100

2696868927430510170611116100

81974251822235

611

611

611

611

611

81974191811235

81974191811235

81974191811235

85

85

8197419101123

8197419101123

7979

7979

7979

7979

7979

7979

6

6

6

6

6

6

11271653657

11271653657

11271653657

11271653657

11271653657

11271653657
