# Supplementary figures and images for: Bacterial diversity of herbal rhizospheric soils in Ordos desert steppes under different degradation gradients
Source: PeerJ. 2023 Nov 1;11:e16289. doi: 10.7717/peerj.16289 (PMC10625353; doi:10.7717/peerj.16289)

# Length Distribution of Sequences

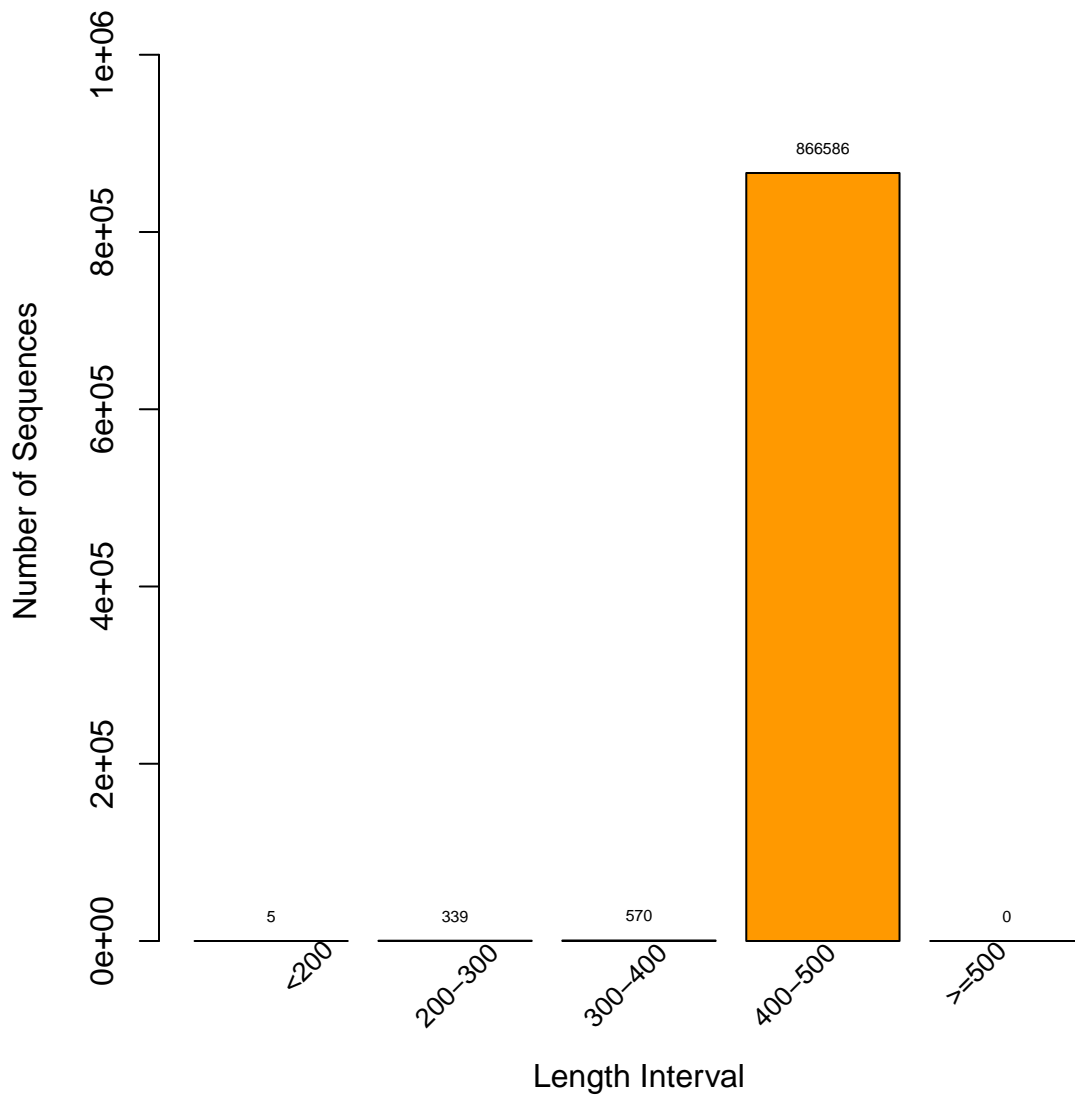

Supplement: Supplemental Information 1 [file peerj-11-16289-s001.zip › 2_clean_data/all_samples_len_dist.pdf]

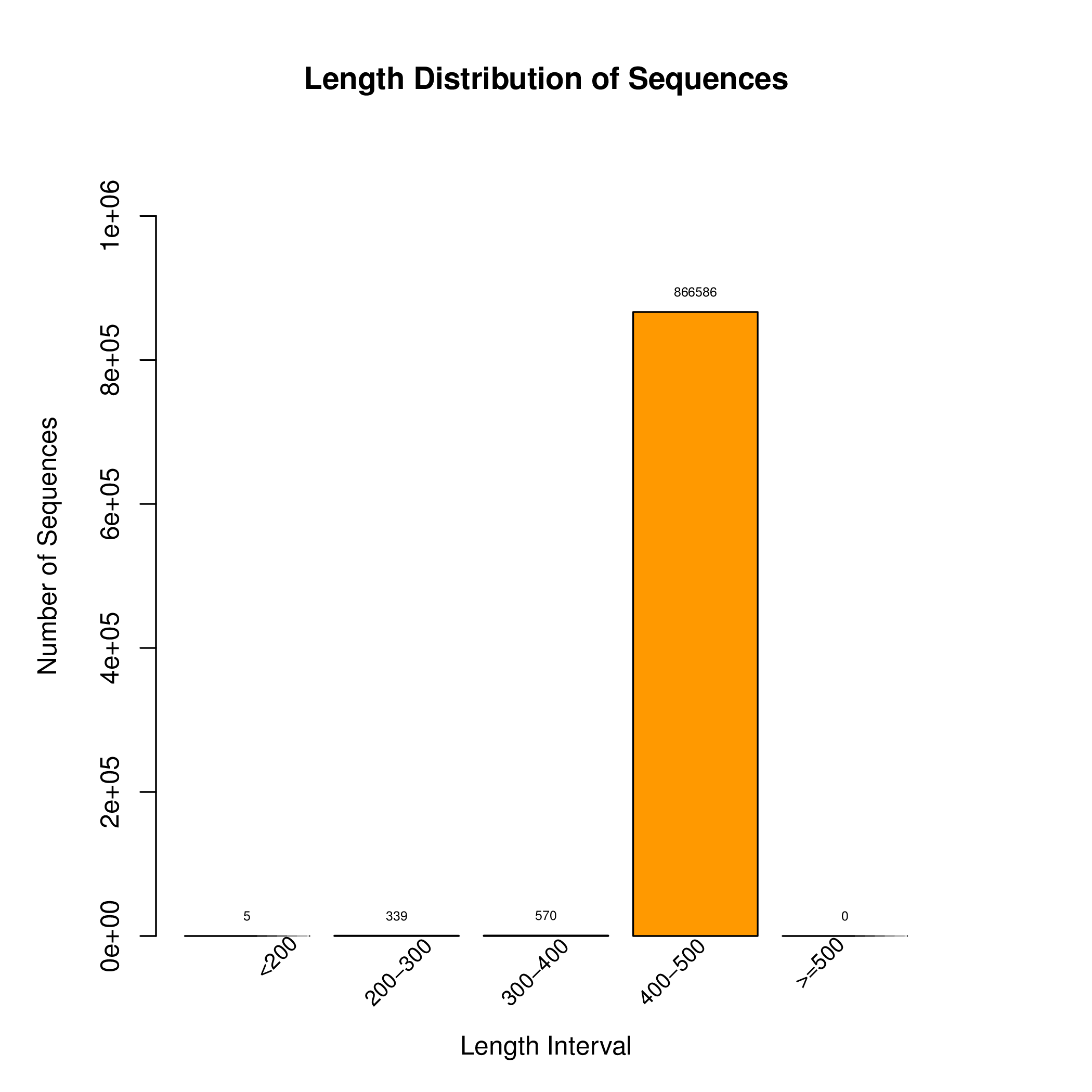

Supplement: Supplemental Information 1 [file peerj-11-16289-s001.zip › 2_clean_data/all_samples_len_dist.png]

# Length Distribution of Sequences

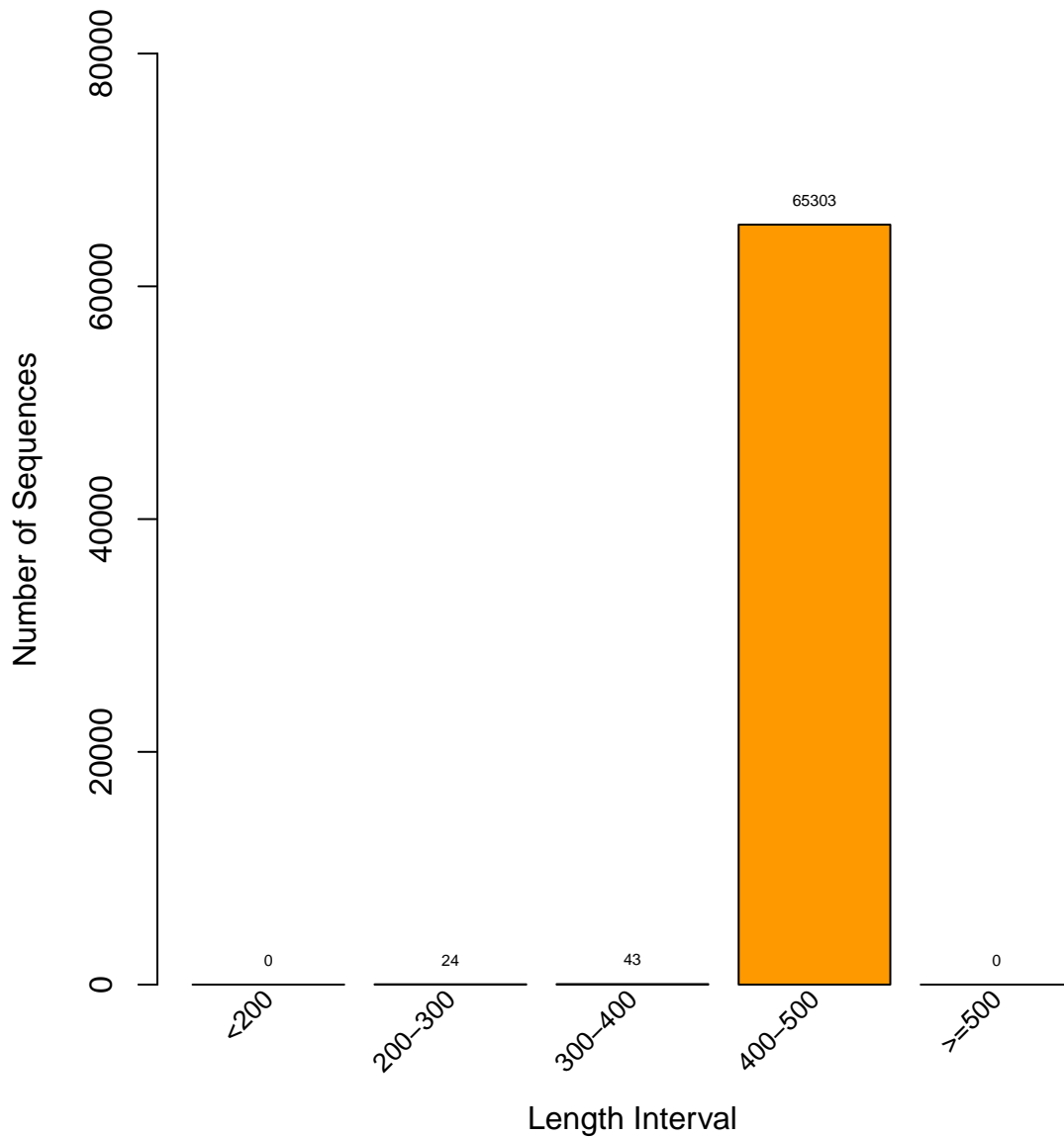

Supplement: Supplemental Information 1 [file peerj-11-16289-s001.zip › 2_clean_data/W1/W1_len_dist.pdf]

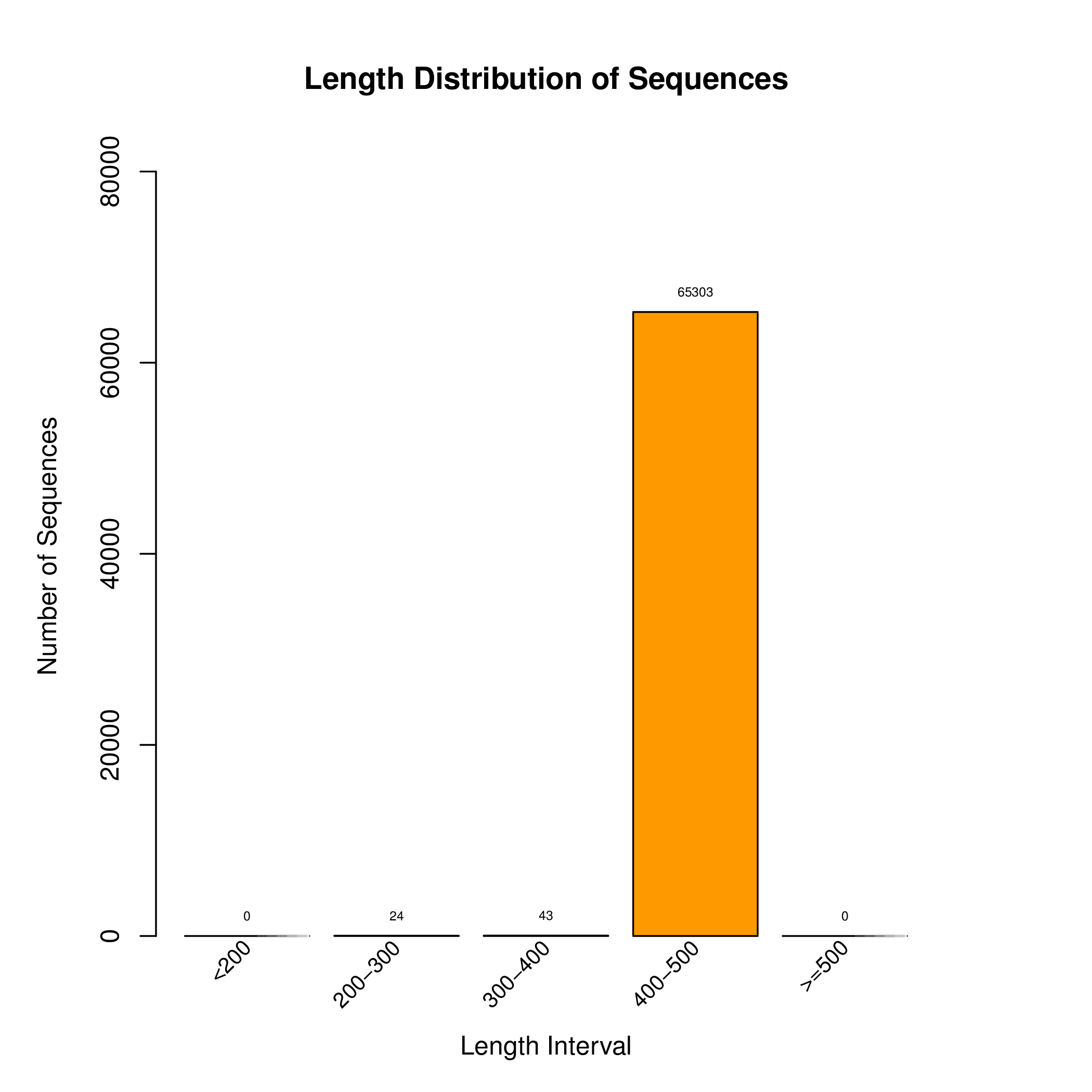

Supplement: Supplemental Information 1 [file peerj-11-16289-s001.zip › 2_clean_data/W1/W1_len_dist.png]

# Length Distribution of Sequences

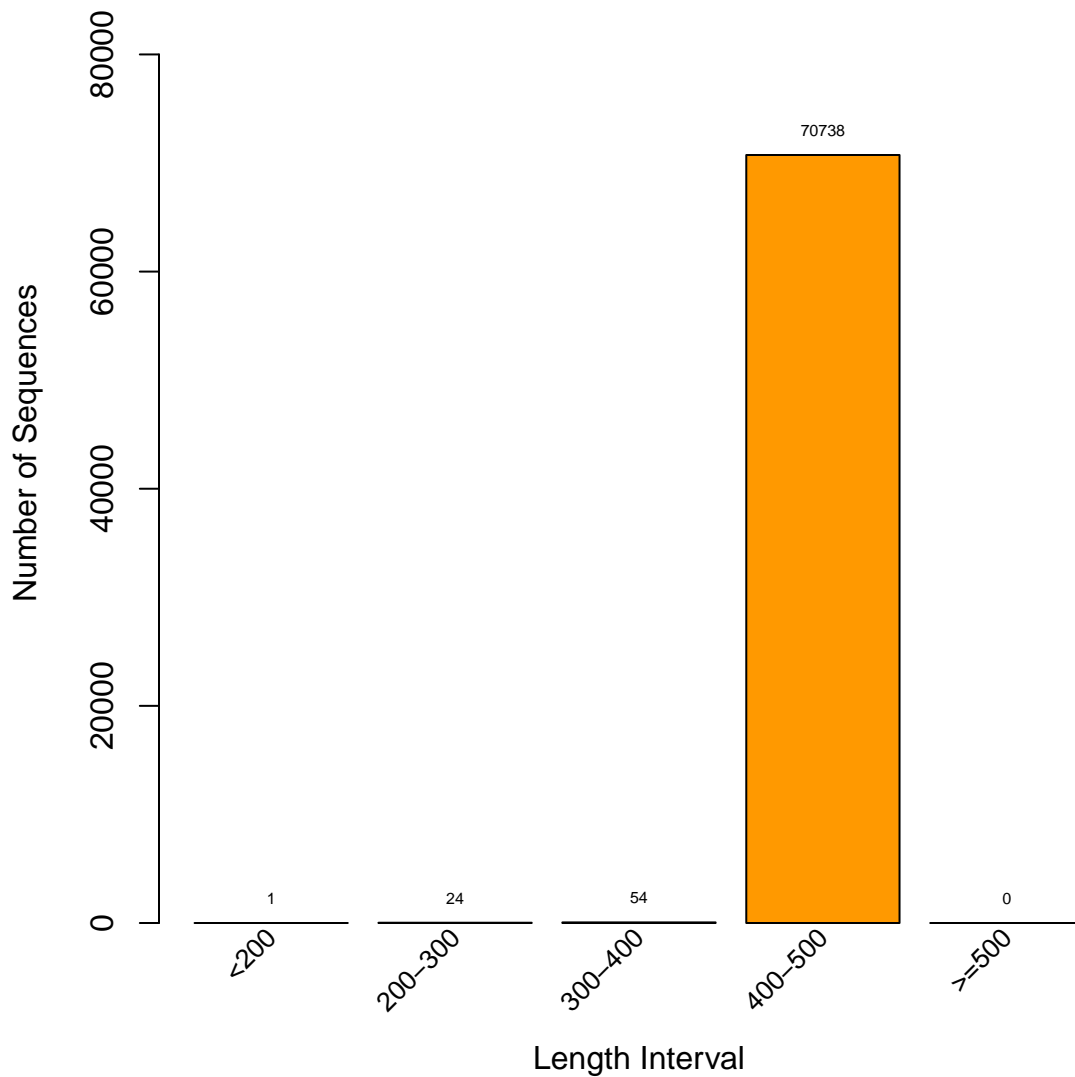

Supplement: Supplemental Information 1 [file peerj-11-16289-s001.zip › 2_clean_data/W10/W10_len_dist.pdf]

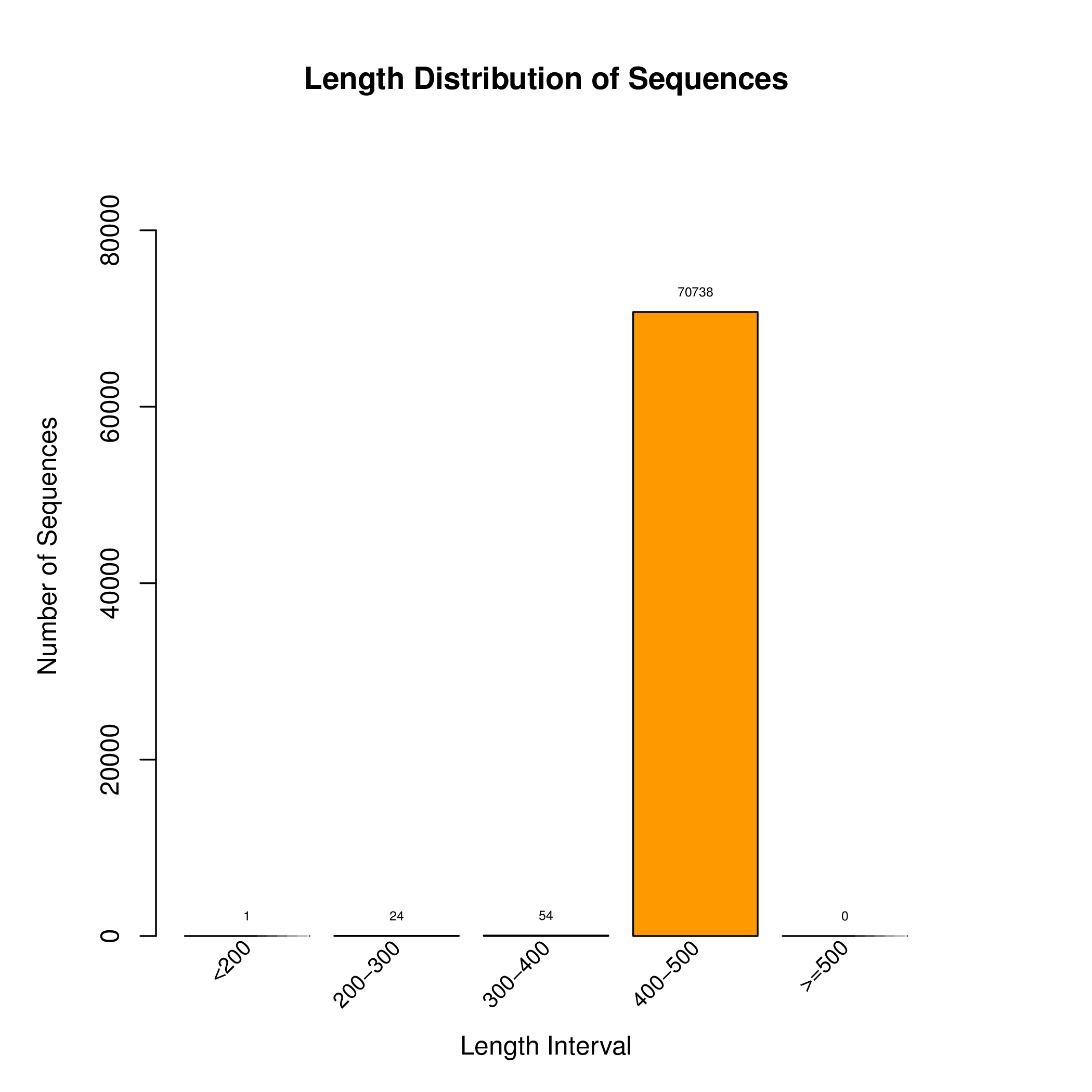

Supplement: Supplemental Information 1 [file peerj-11-16289-s001.zip › 2_clean_data/W10/W10_len_dist.png]

# Length Distribution of Sequences

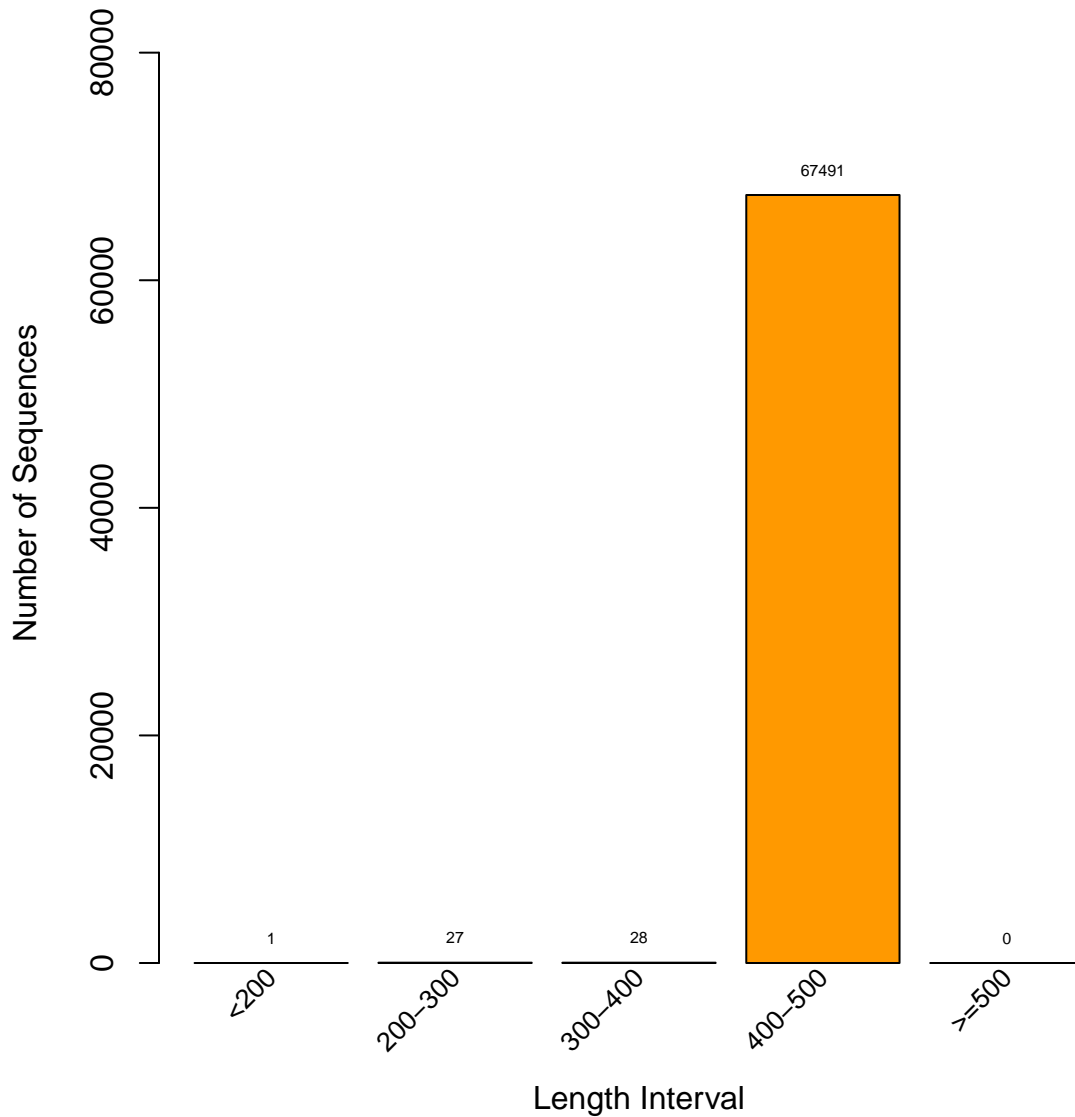

Supplement: Supplemental Information 1 [file peerj-11-16289-s001.zip › 2_clean_data/W11/W11_len_dist.pdf]

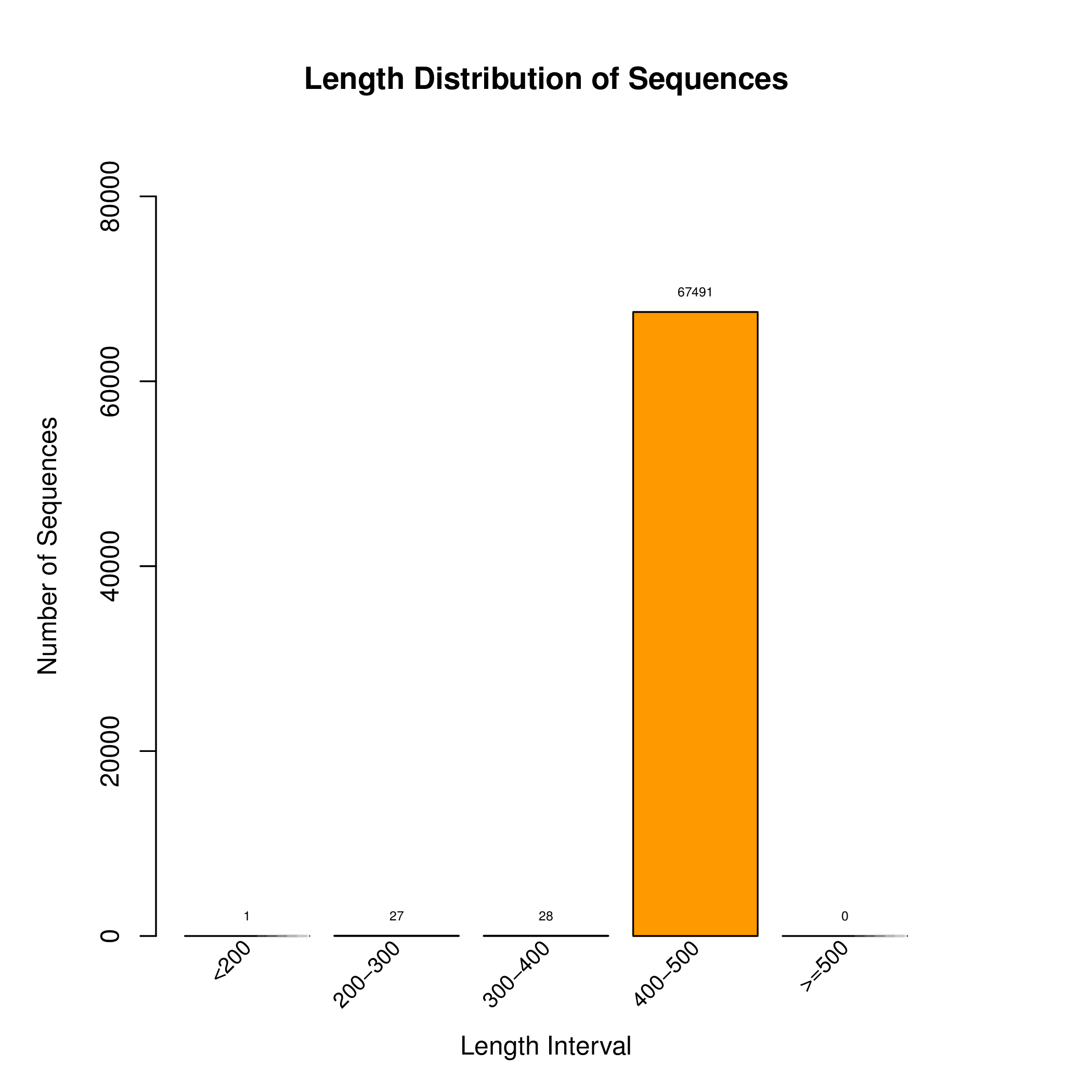

Supplement: Supplemental Information 1 [file peerj-11-16289-s001.zip › 2_clean_data/W11/W11_len_dist.png]

# Length Distribution of Sequences

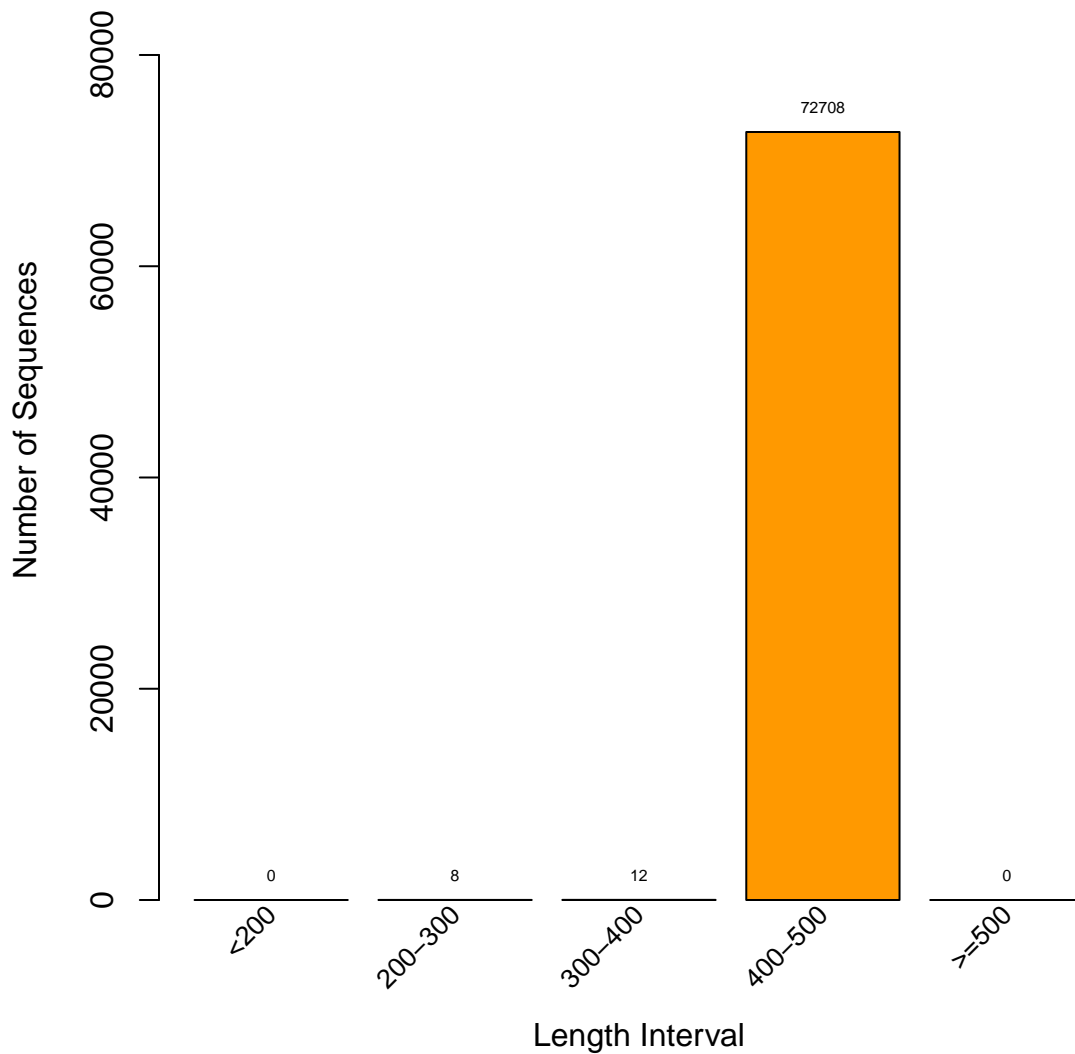

Supplement: Supplemental Information 1 [file peerj-11-16289-s001.zip › 2_clean_data/W12/W12_len_dist.pdf]

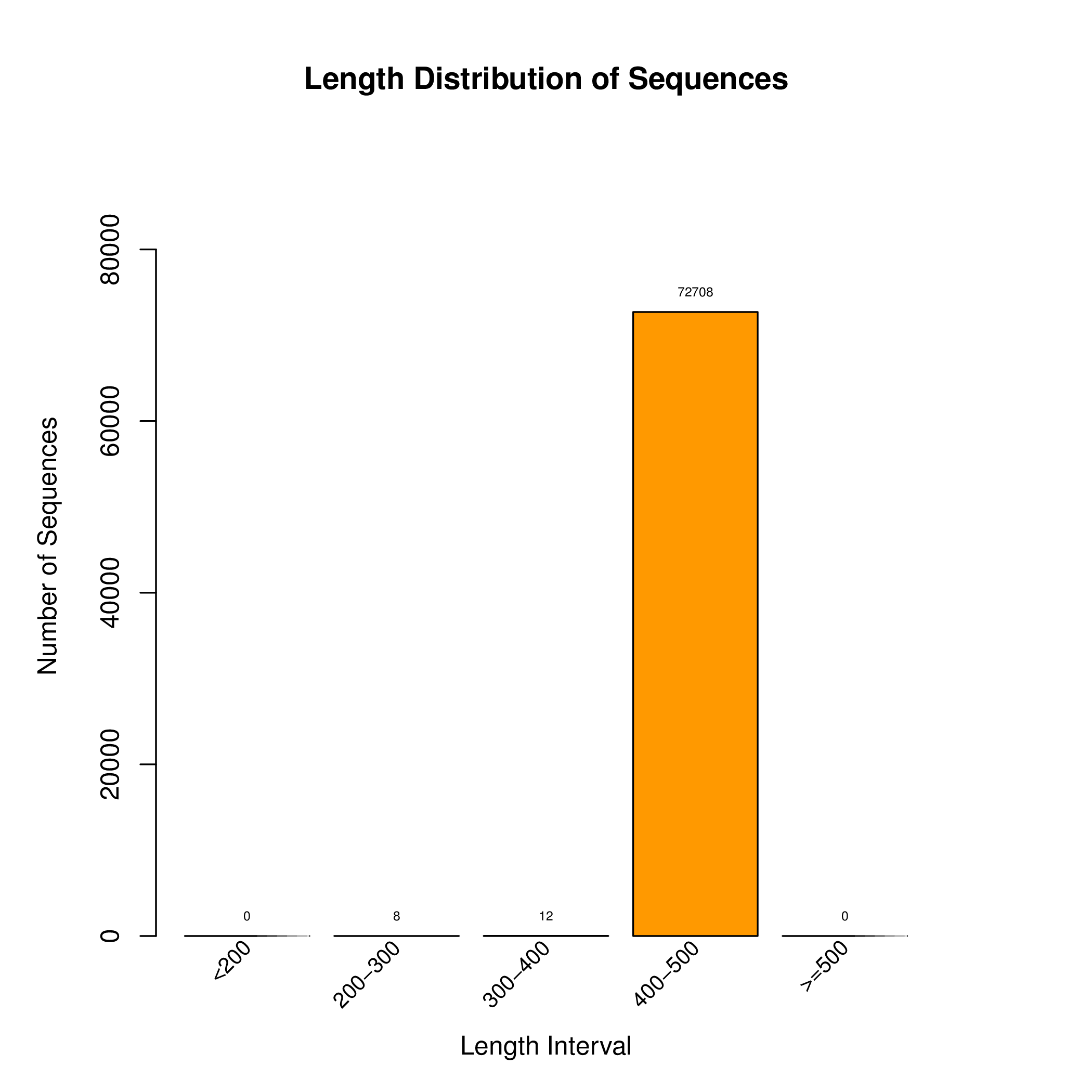

Supplement: Supplemental Information 1 [file peerj-11-16289-s001.zip › 2_clean_data/W12/W12_len_dist.png]

# Length Distribution of Sequences

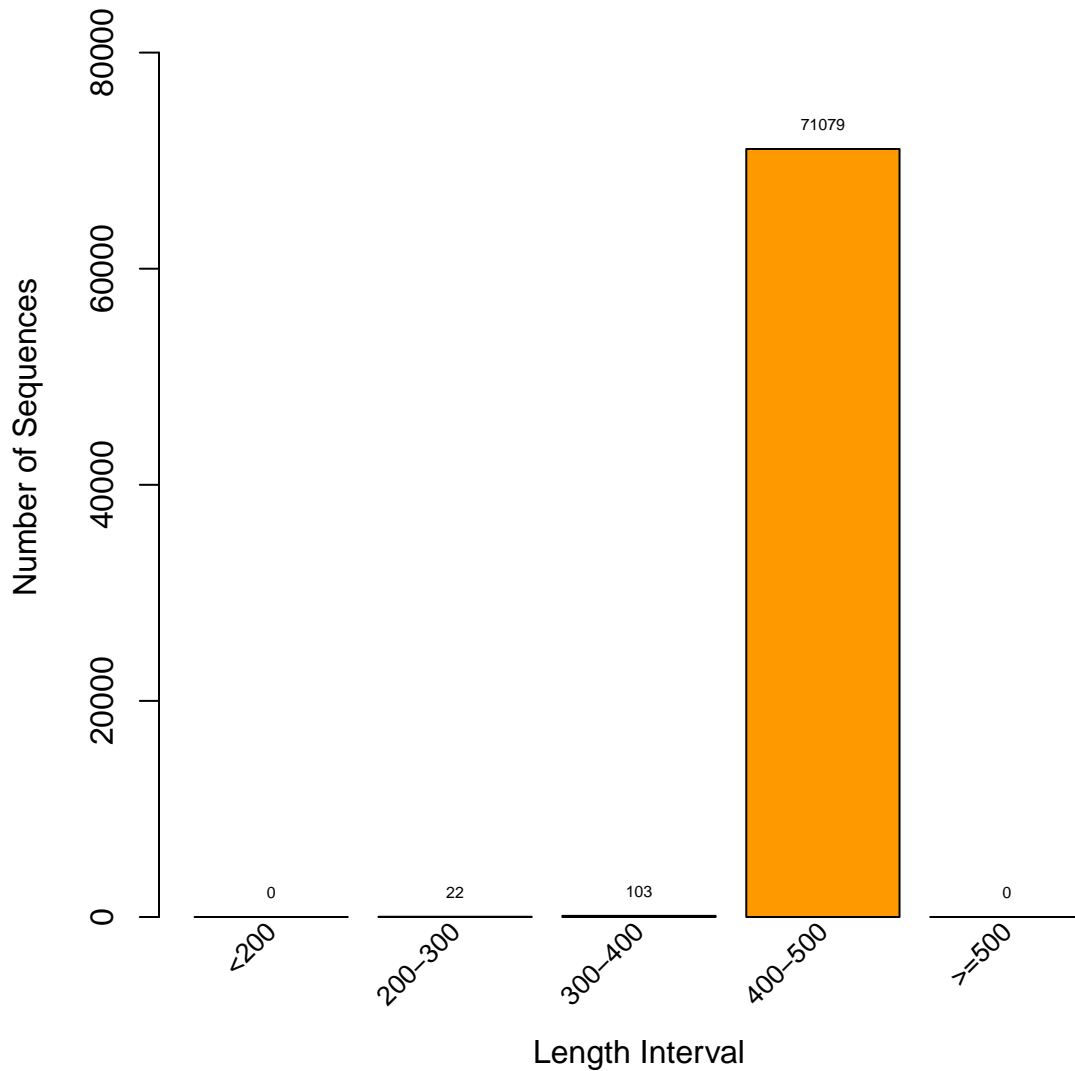

Supplement: Supplemental Information 1 [file peerj-11-16289-s001.zip › 2_clean_data/W13/W13_len_dist.pdf]

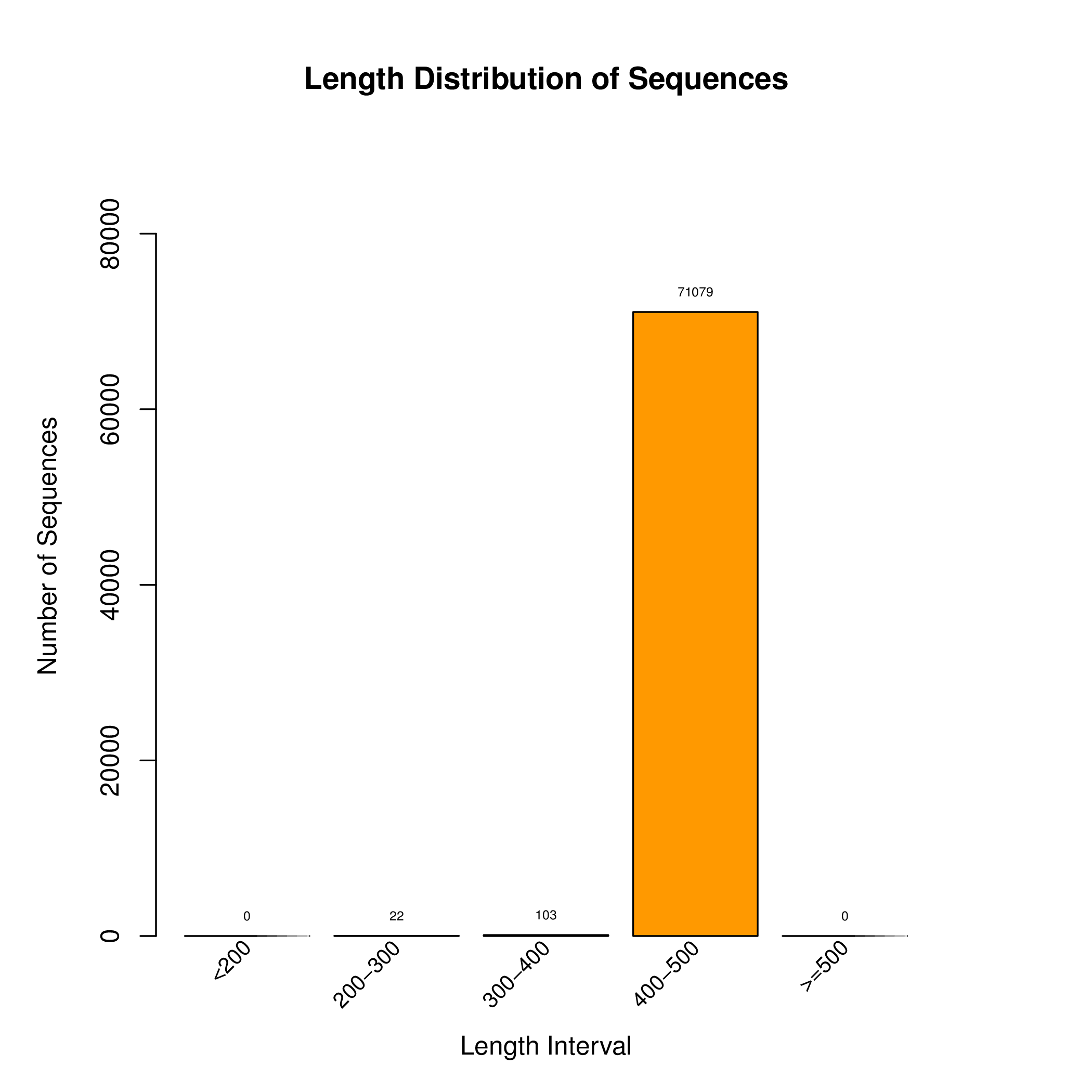

Supplement: Supplemental Information 1 [file peerj-11-16289-s001.zip › 2_clean_data/W13/W13_len_dist.png]

# Length Distribution of Sequences

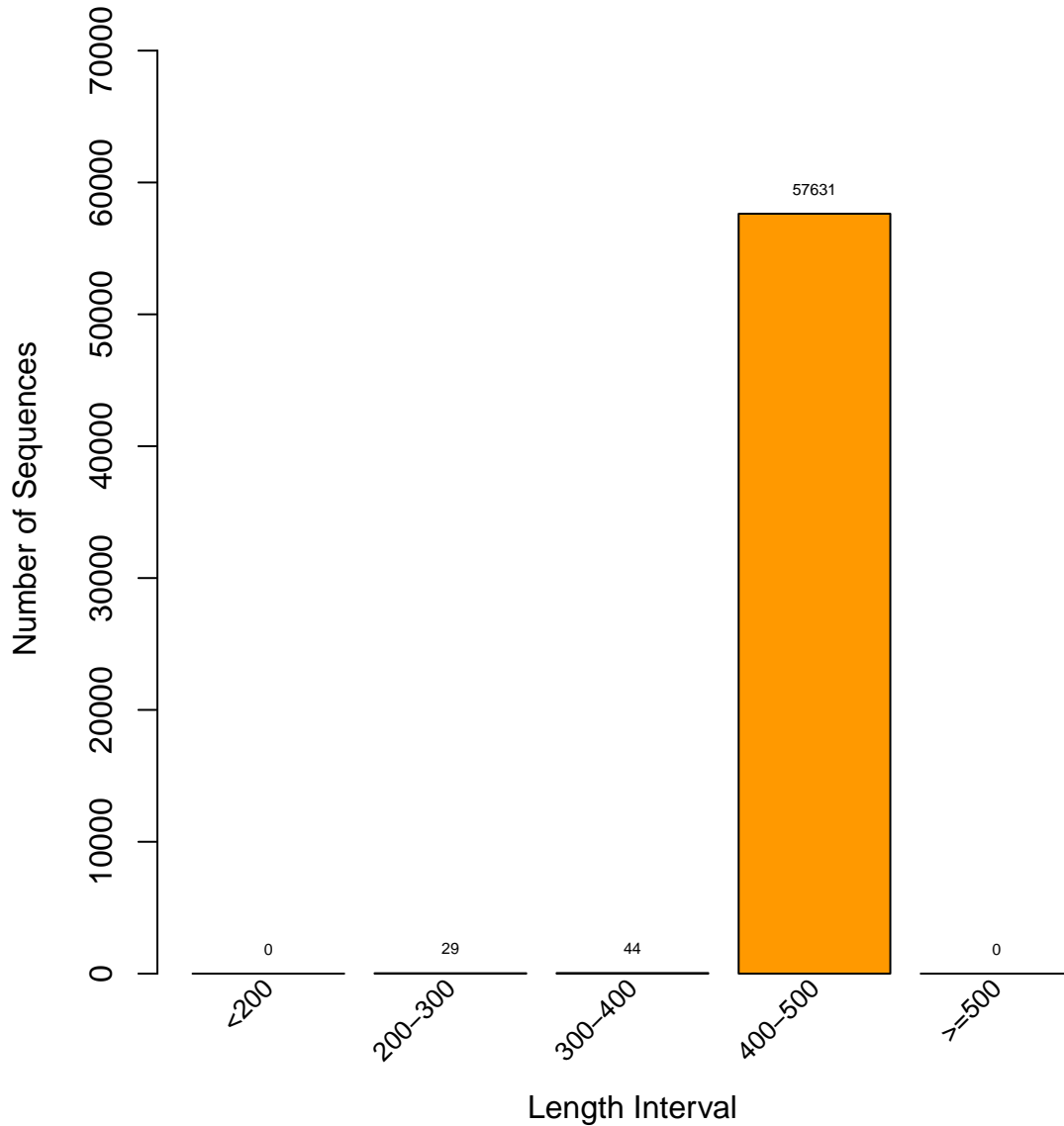

Supplement: Supplemental Information 1 [file peerj-11-16289-s001.zip › 2_clean_data/W2/W2_len_dist.pdf]

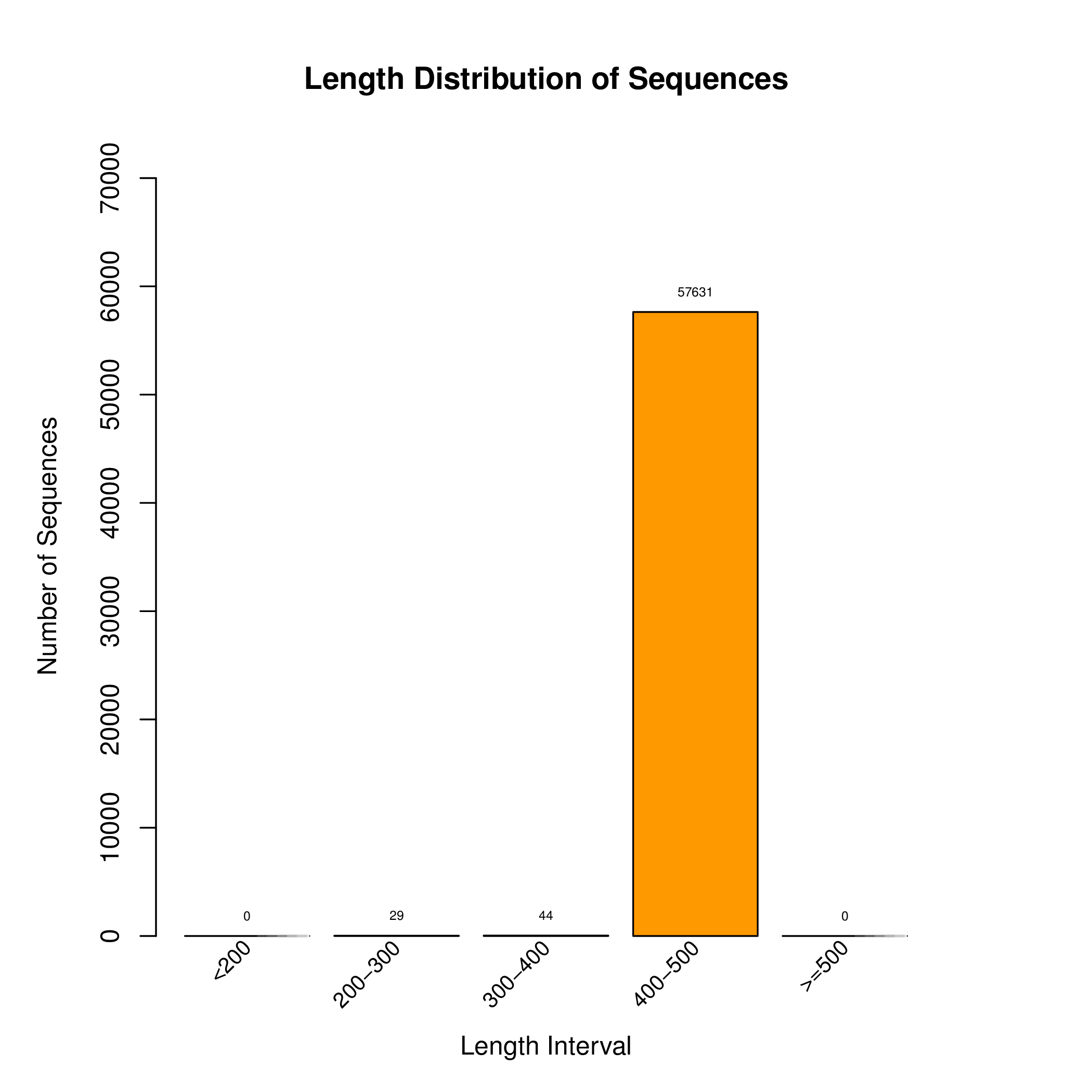

Supplement: Supplemental Information 1 [file peerj-11-16289-s001.zip › 2_clean_data/W2/W2_len_dist.png]

# Length Distribution of Sequences

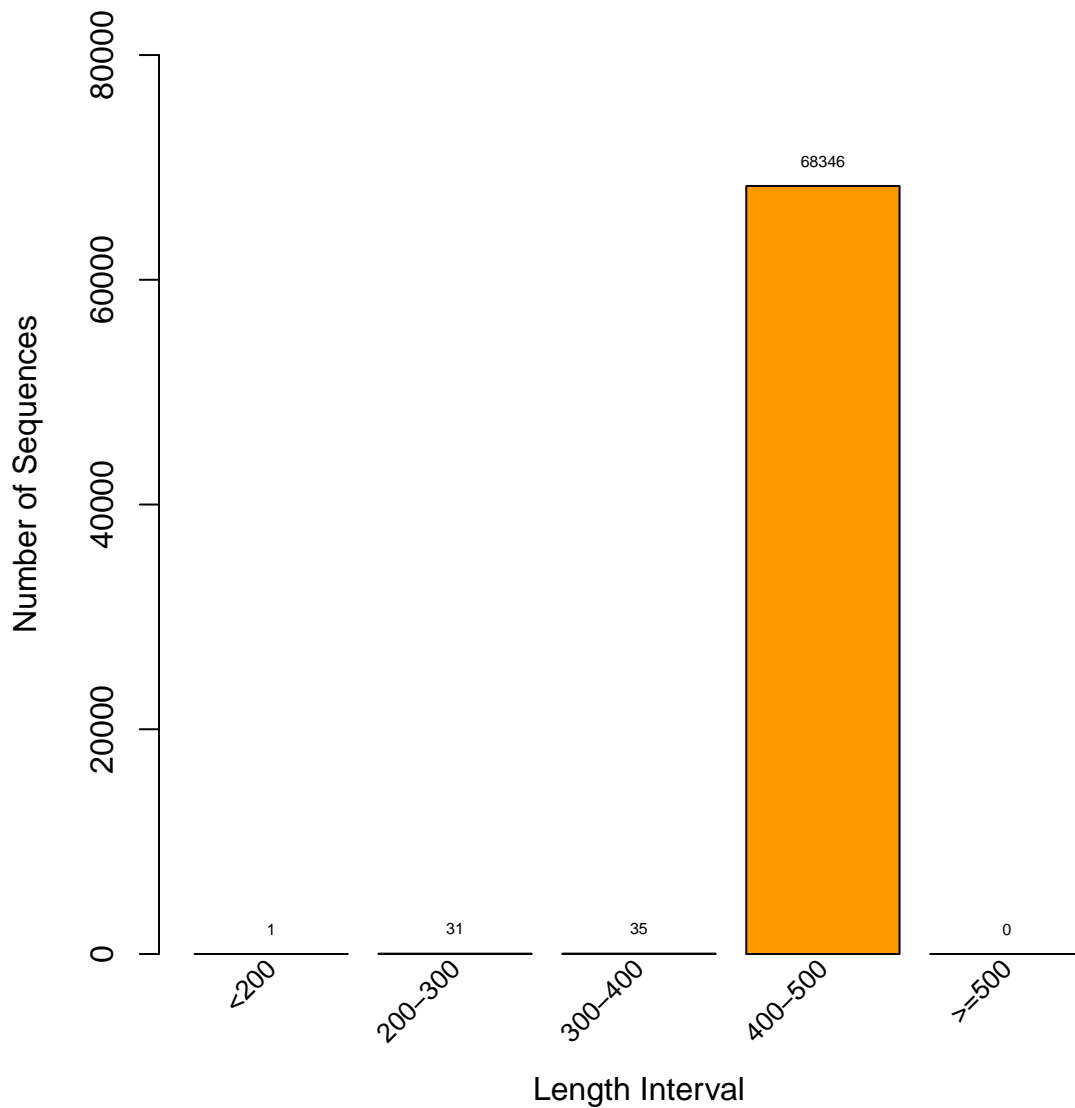

Supplement: Supplemental Information 1 [file peerj-11-16289-s001.zip › 2_clean_data/W3/W3_len_dist.pdf]

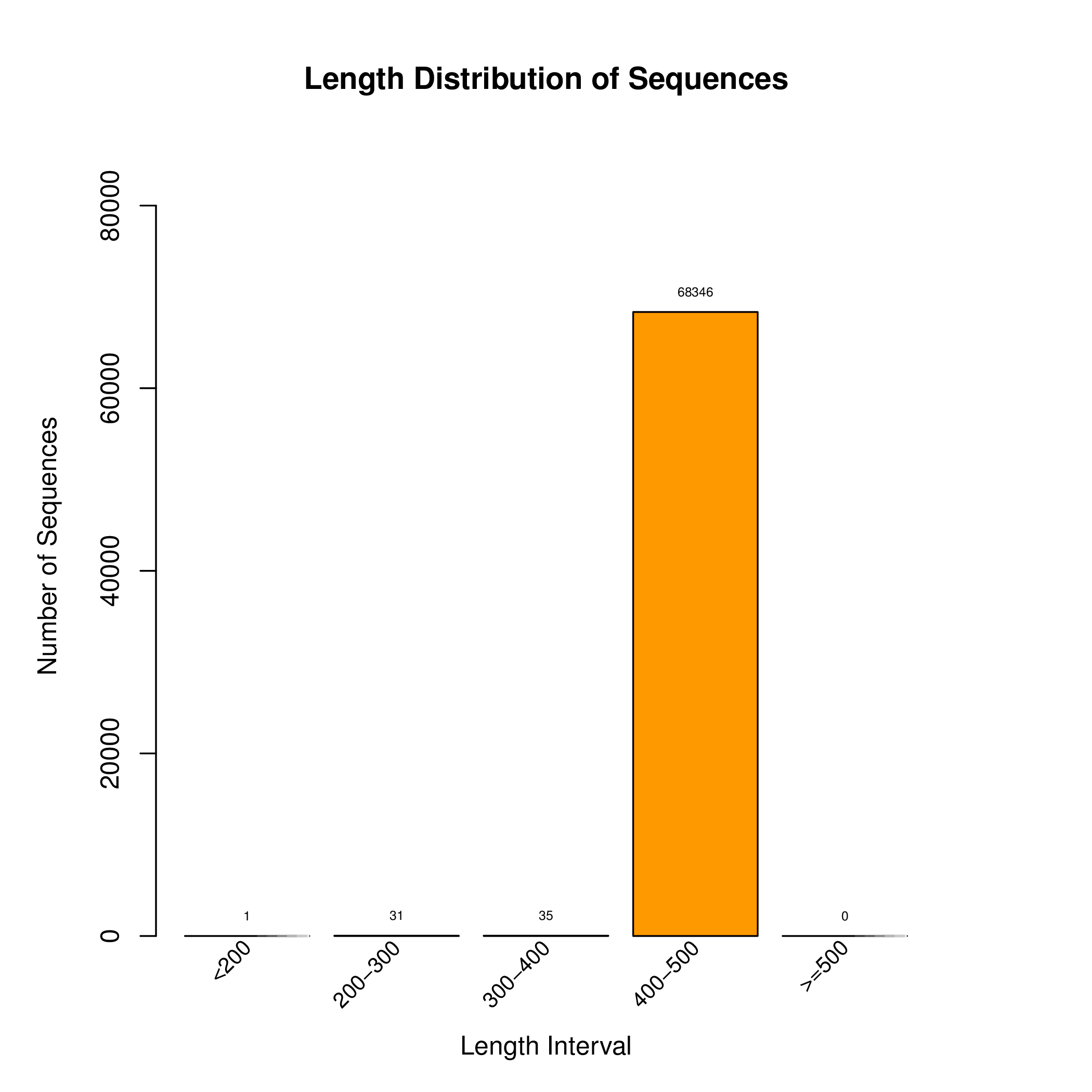

Supplement: Supplemental Information 1 [file peerj-11-16289-s001.zip › 2_clean_data/W3/W3_len_dist.png]

# Length Distribution of Sequences

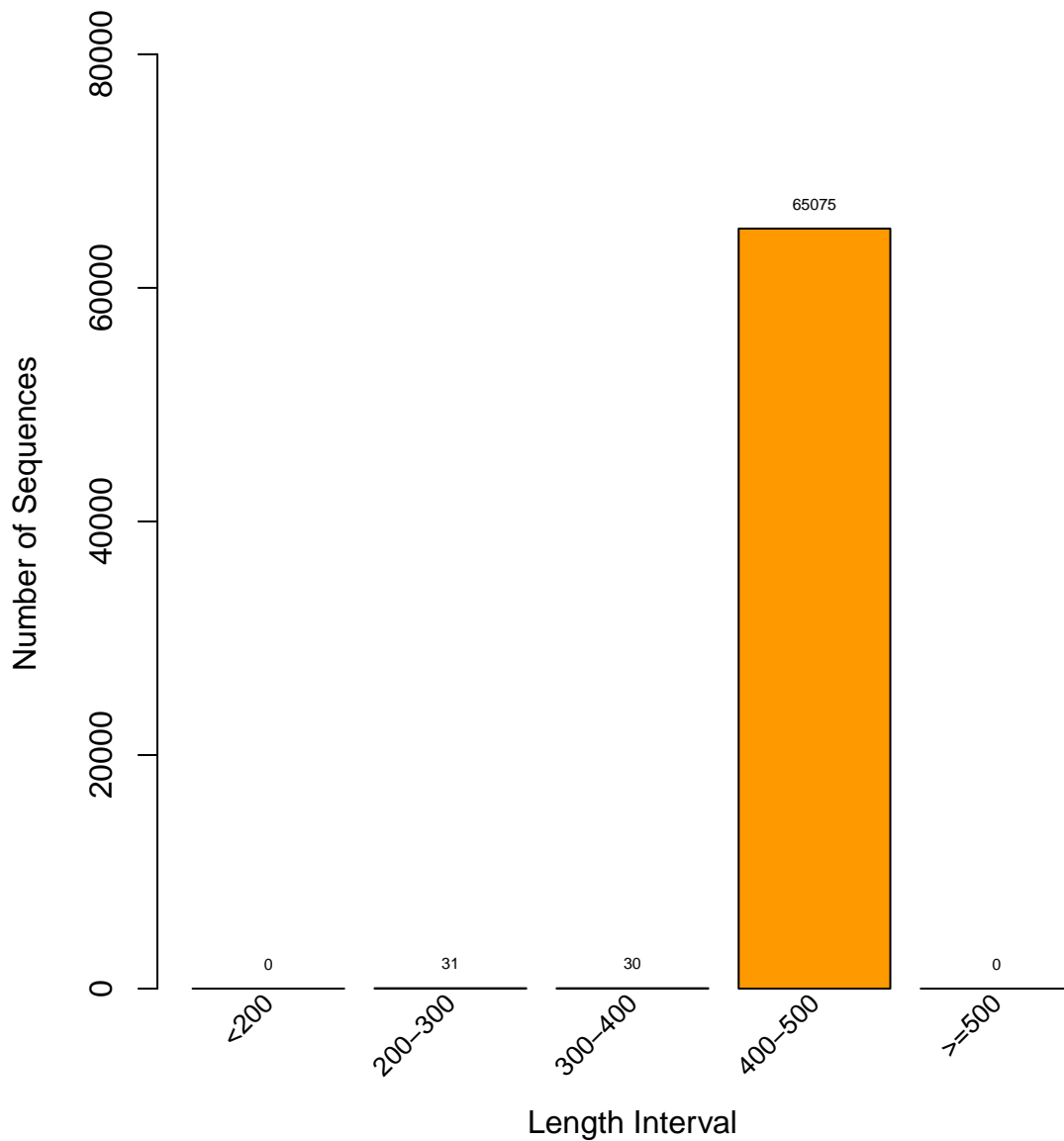

Supplement: Supplemental Information 1 [file peerj-11-16289-s001.zip › 2_clean_data/W4/W4_len_dist.pdf]

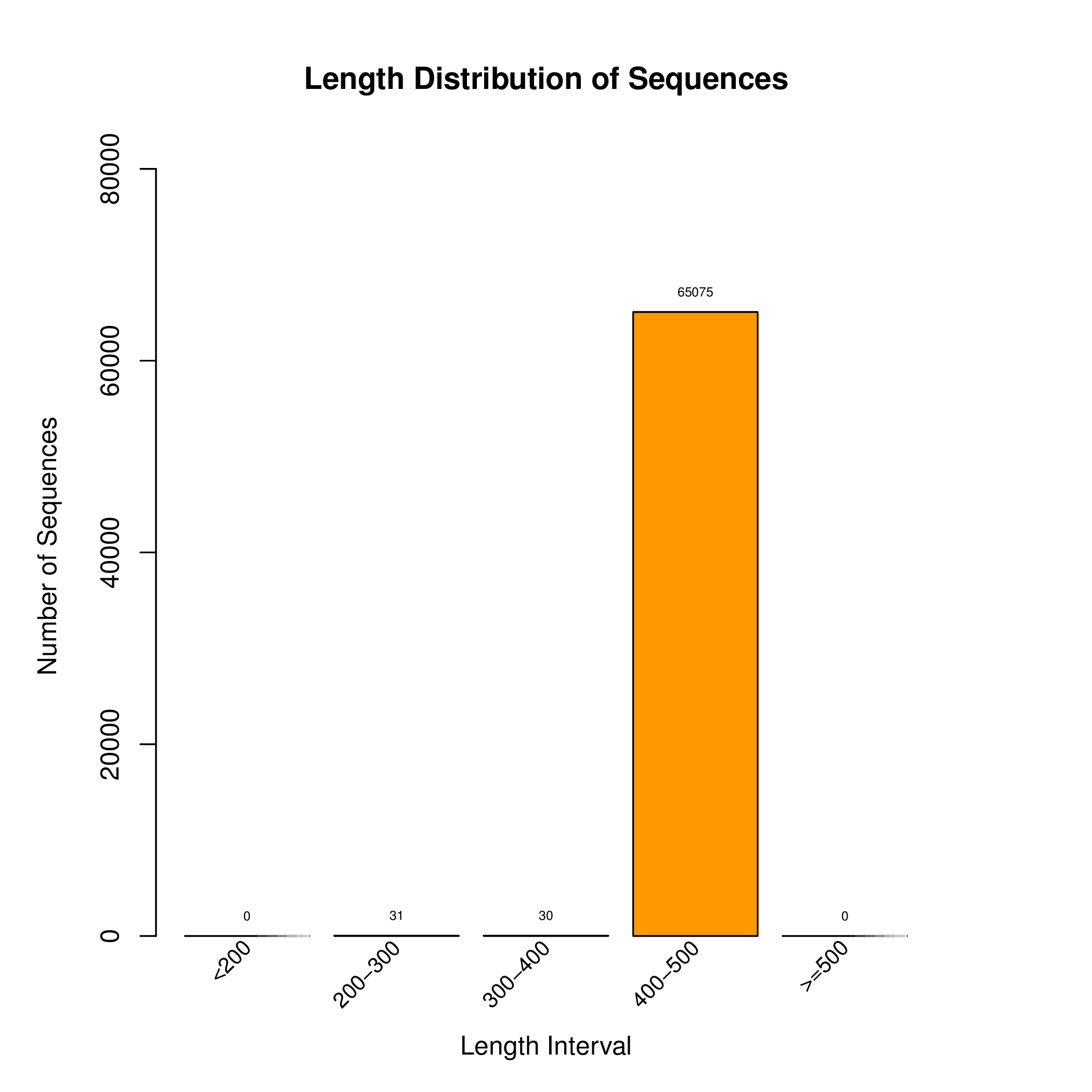

Supplement: Supplemental Information 1 [file peerj-11-16289-s001.zip › 2_clean_data/W4/W4_len_dist.png]

# Length Distribution of Sequences

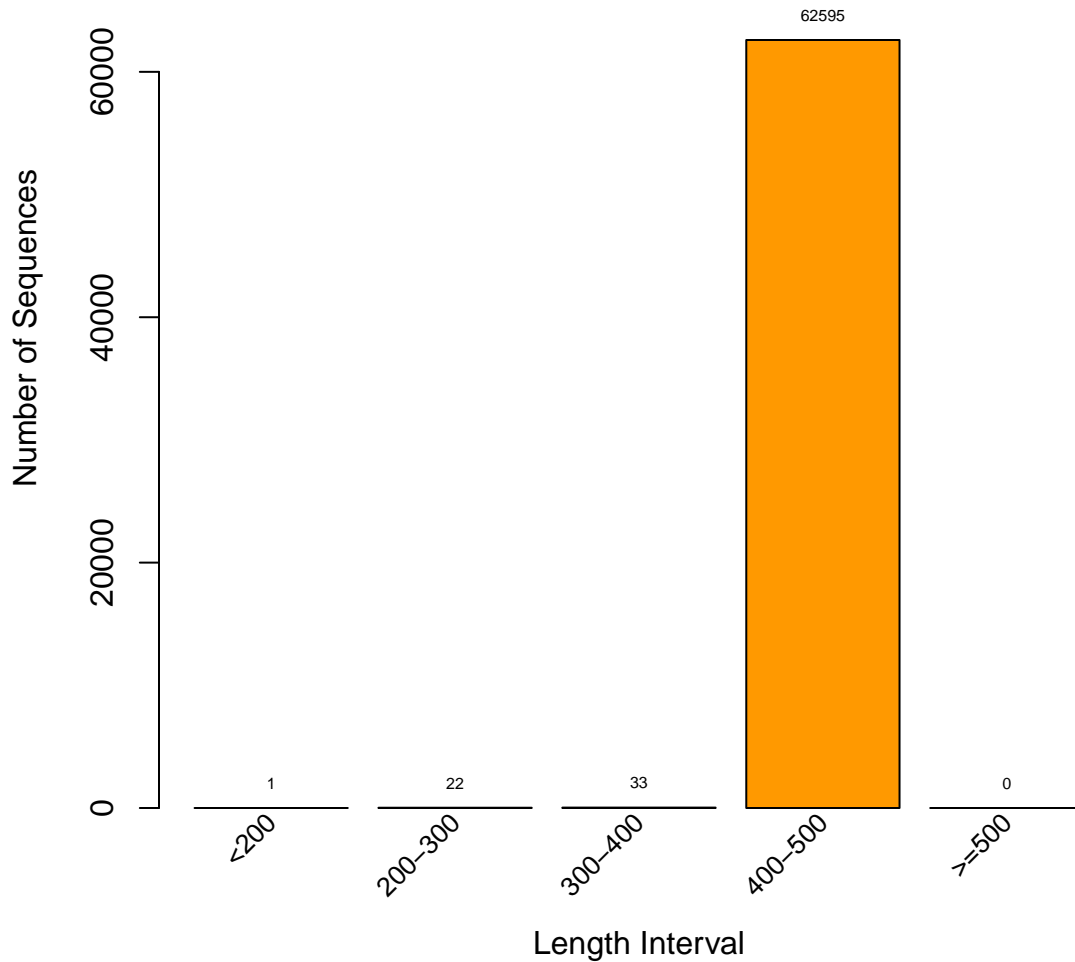

Supplement: Supplemental Information 1 [file peerj-11-16289-s001.zip › 2_clean_data/W5/W5_len_dist.pdf]

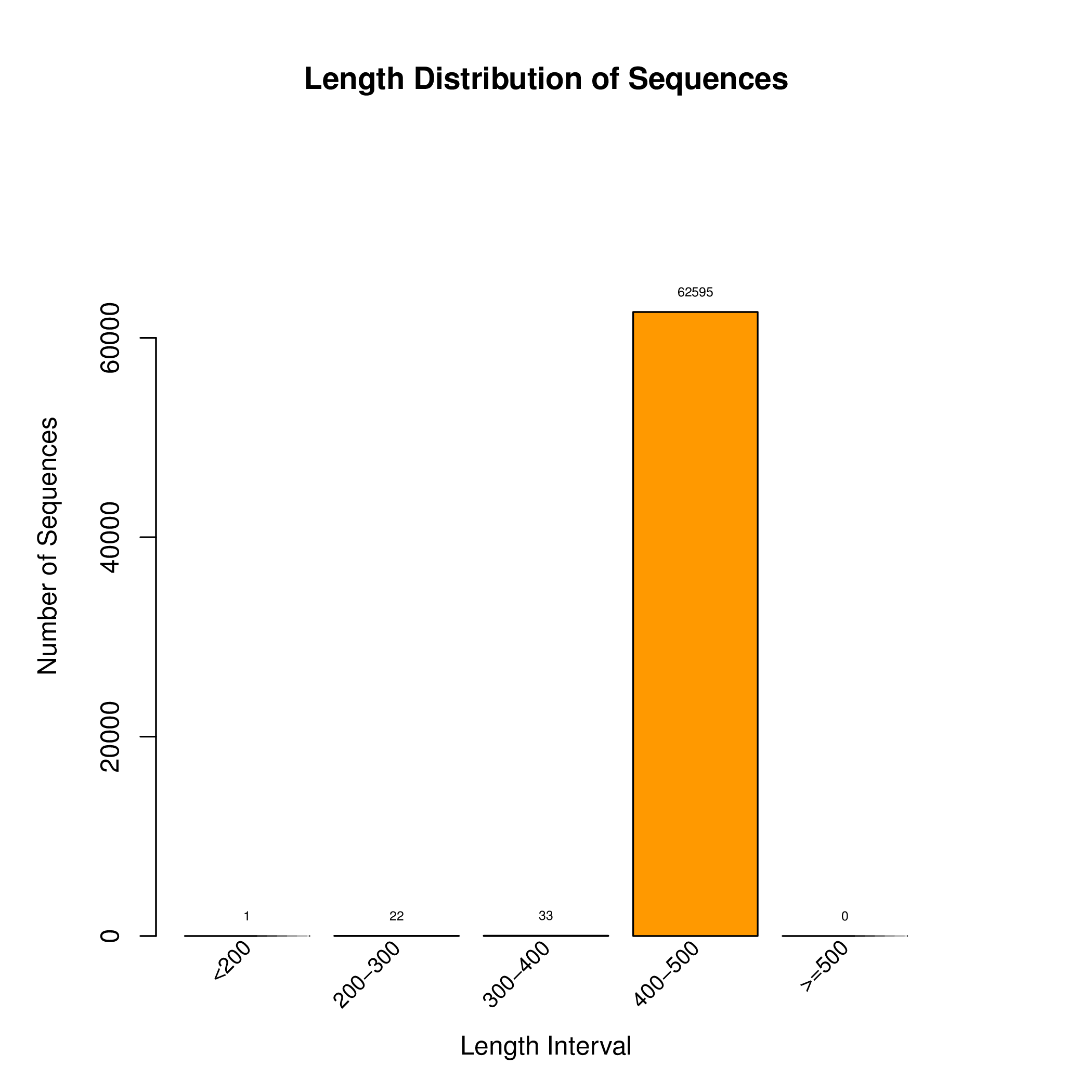

Supplement: Supplemental Information 1 [file peerj-11-16289-s001.zip › 2_clean_data/W5/W5_len_dist.png]

# Length Distribution of Sequences

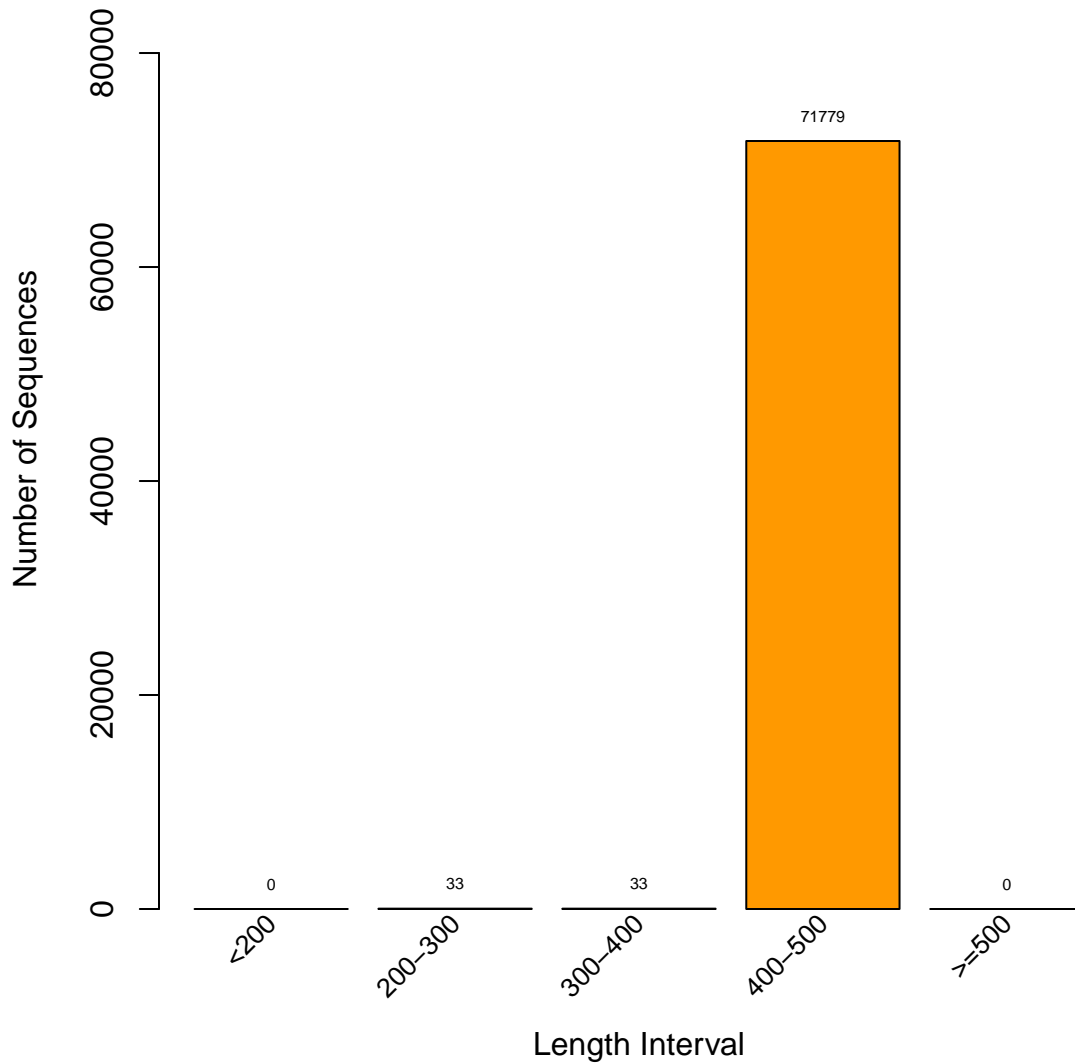

Supplement: Supplemental Information 1 [file peerj-11-16289-s001.zip › 2_clean_data/W6/W6_len_dist.pdf]

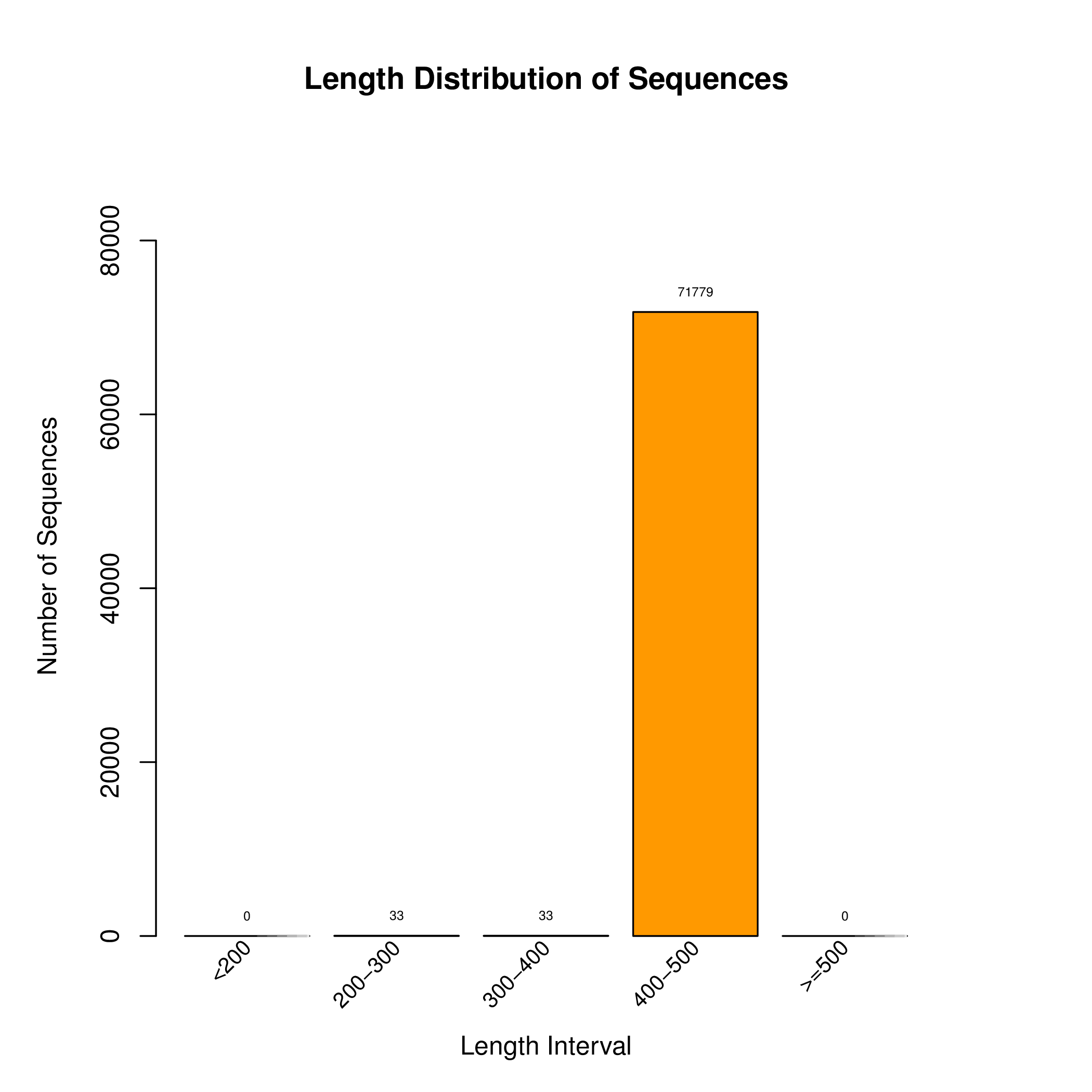

Supplement: Supplemental Information 1 [file peerj-11-16289-s001.zip › 2_clean_data/W6/W6_len_dist.png]

# Length Distribution of Sequences

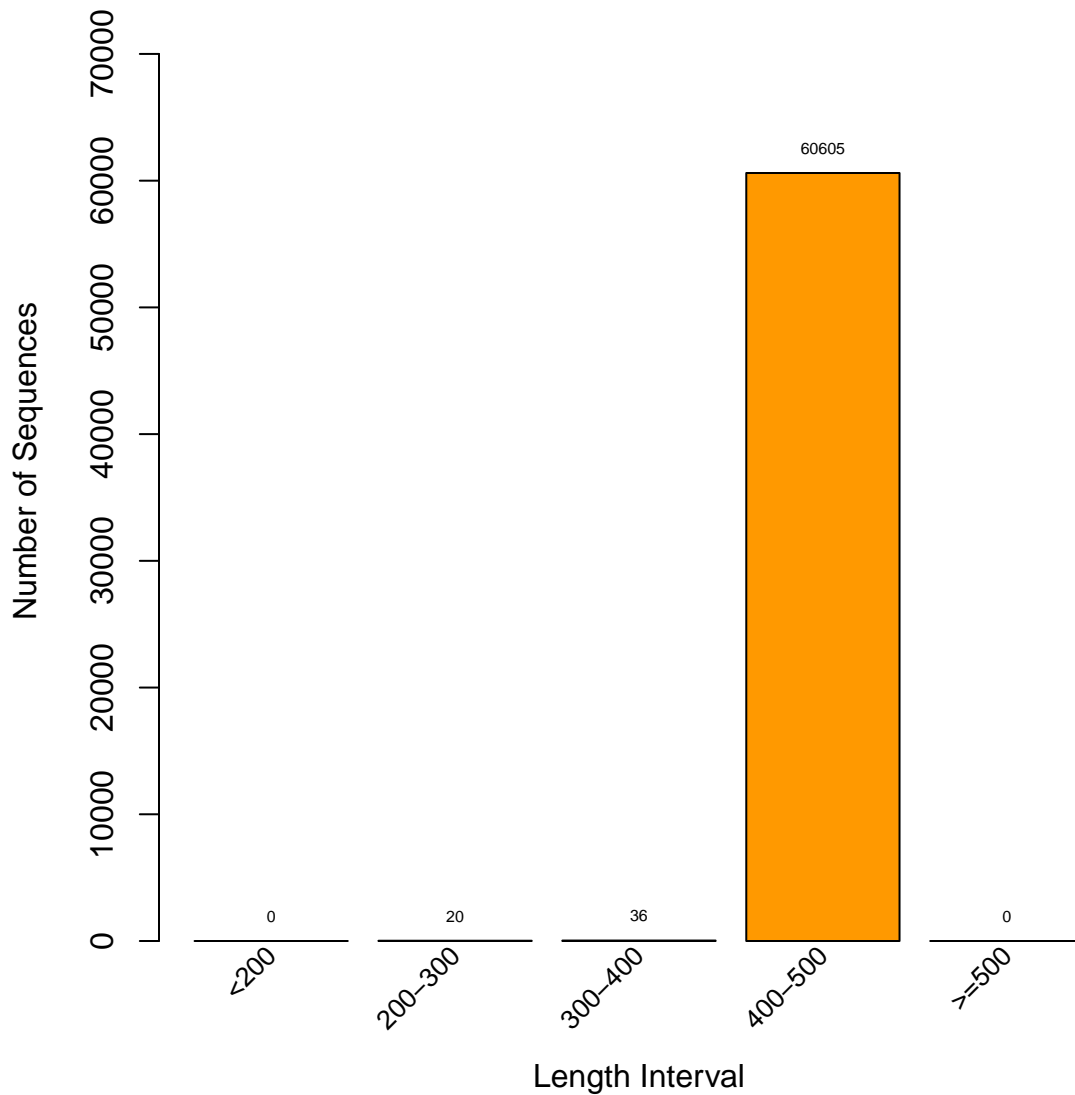

Supplement: Supplemental Information 1 [file peerj-11-16289-s001.zip › 2_clean_data/W7/W7_len_dist.pdf]

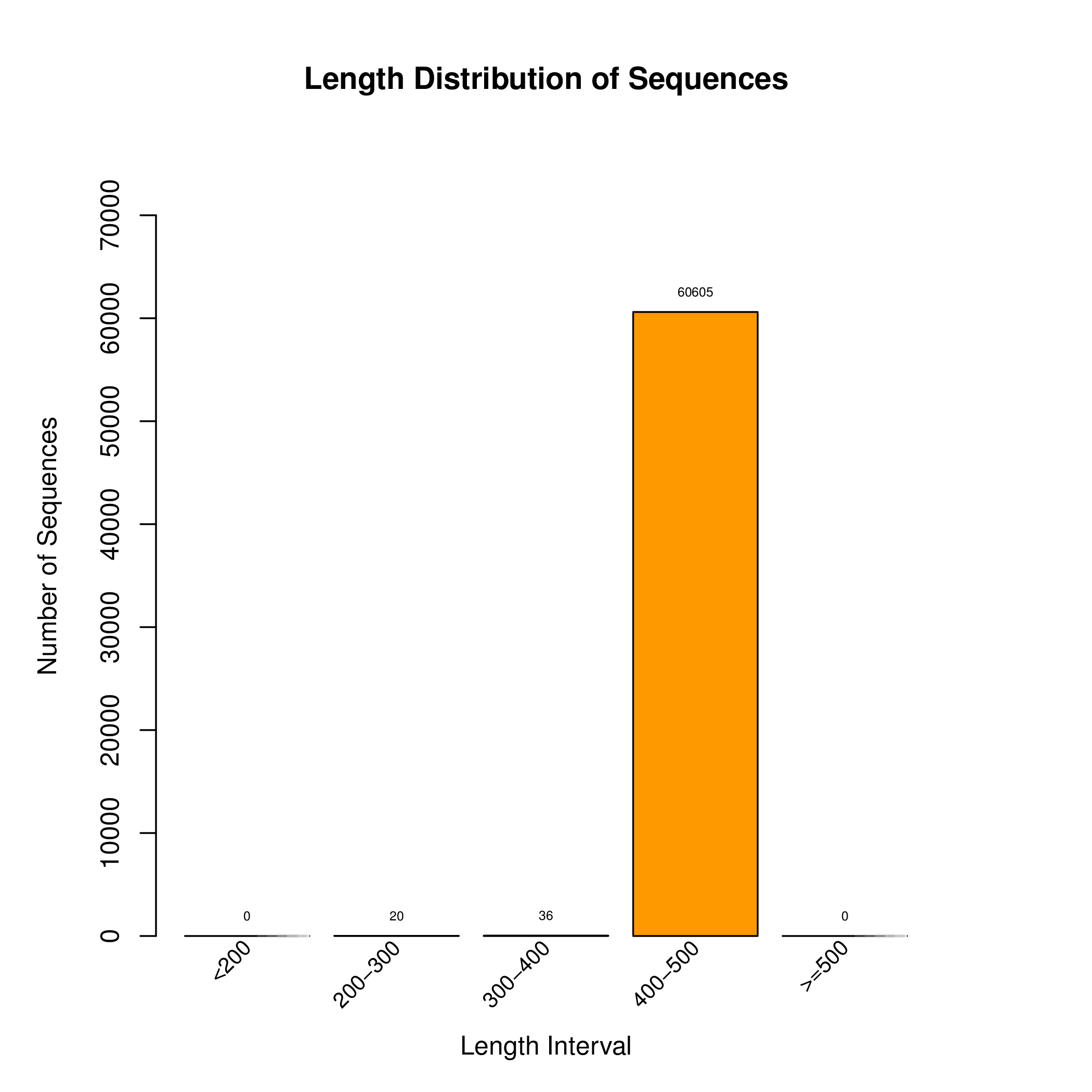

Supplement: Supplemental Information 1 [file peerj-11-16289-s001.zip › 2_clean_data/W7/W7_len_dist.png]

# Length Distribution of Sequences

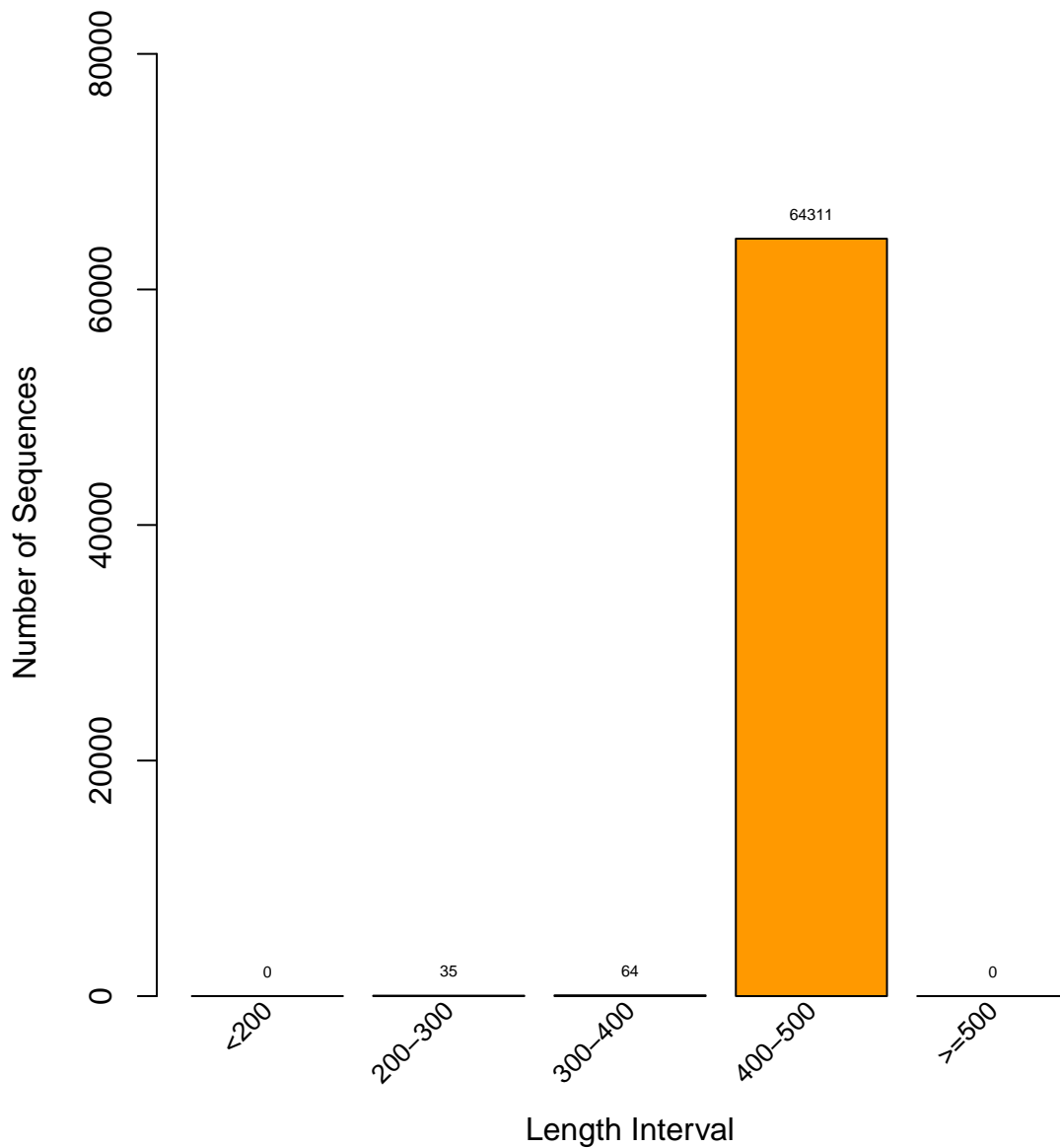

Supplement: Supplemental Information 1 [file peerj-11-16289-s001.zip › 2_clean_data/W8/W8_len_dist.pdf]

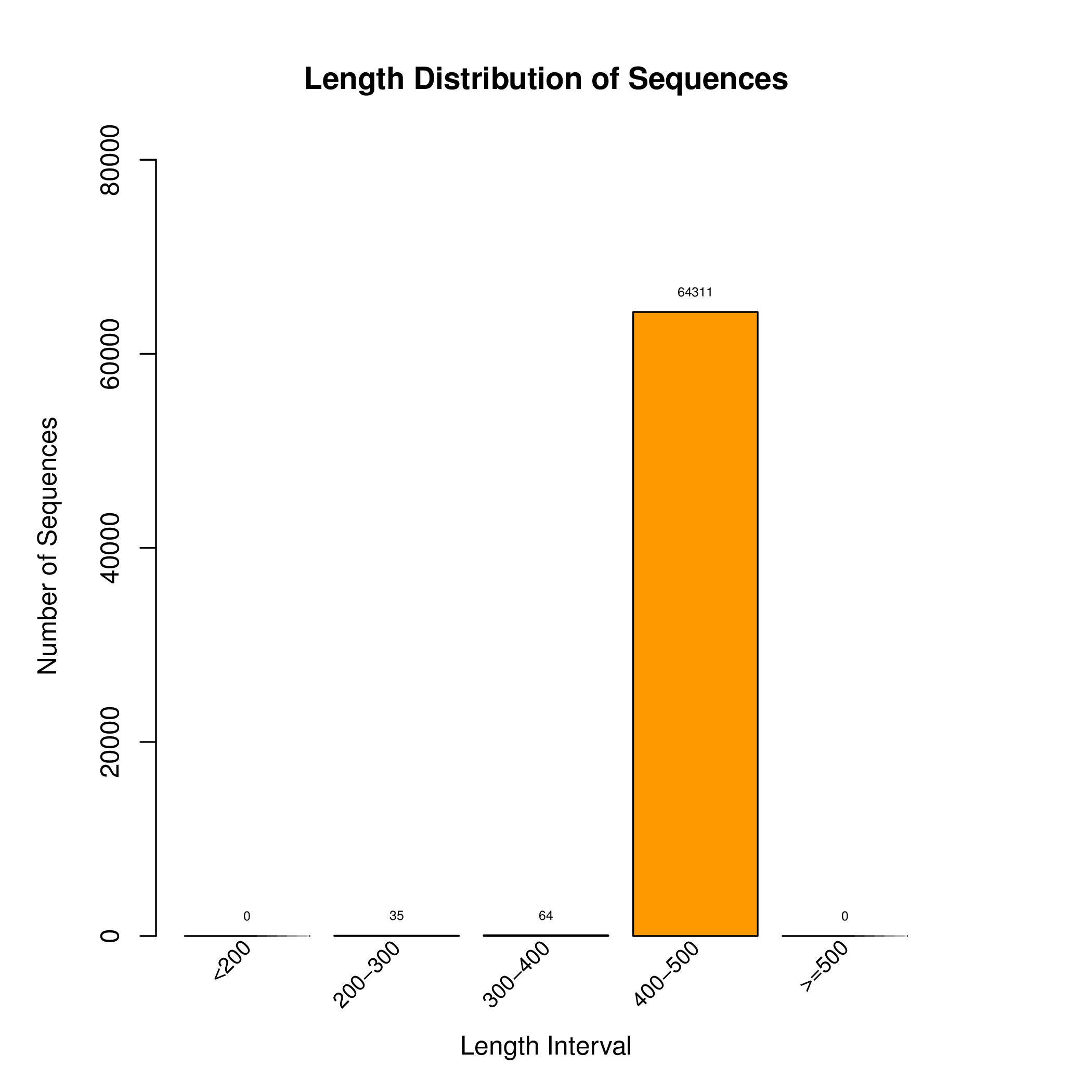

Supplement: Supplemental Information 1 [file peerj-11-16289-s001.zip › 2_clean_data/W8/W8_len_dist.png]

# Length Distribution of Sequences

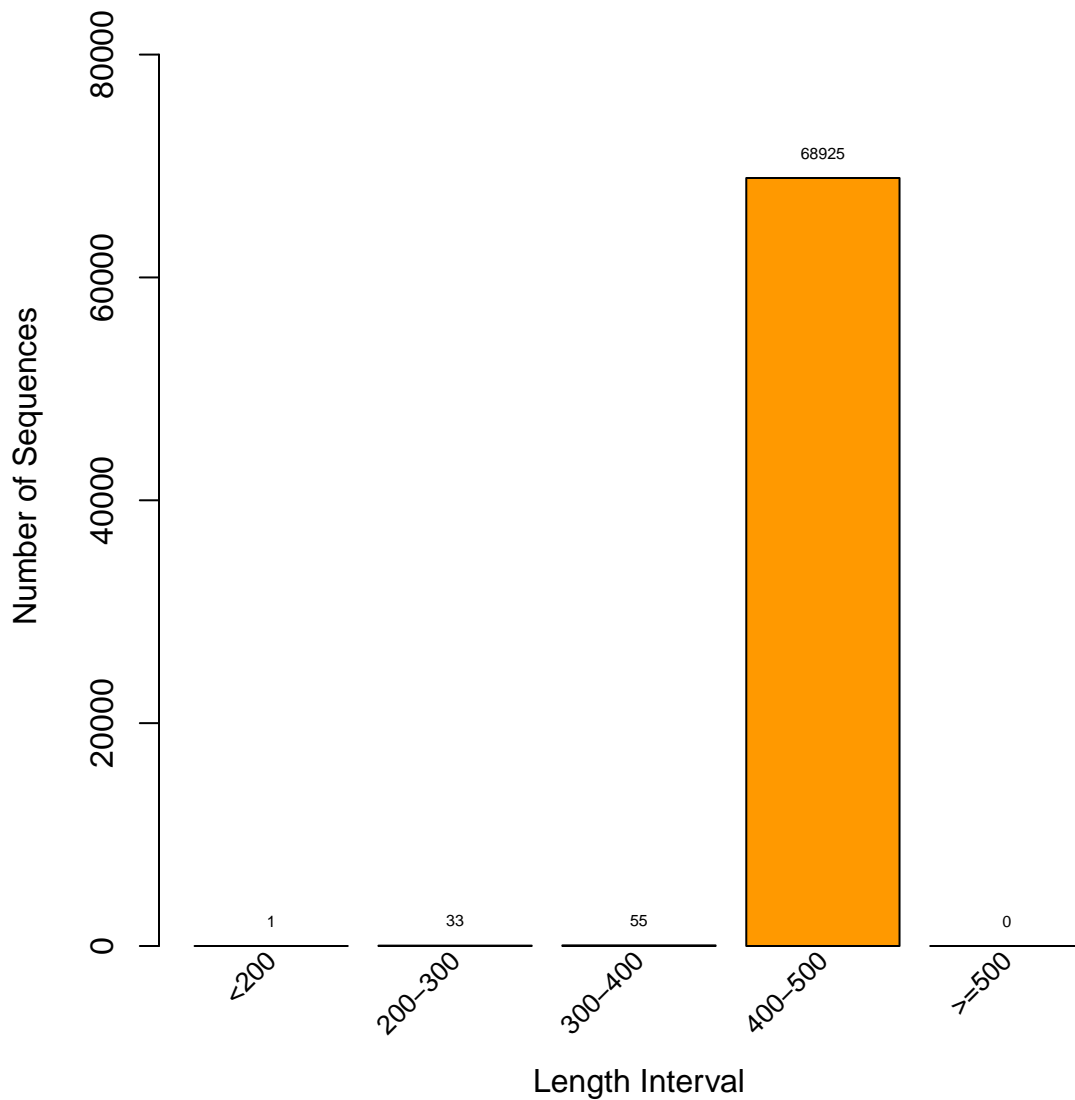

Supplement: Supplemental Information 1 [file peerj-11-16289-s001.zip › 2_clean_data/W9/W9_len_dist.pdf]

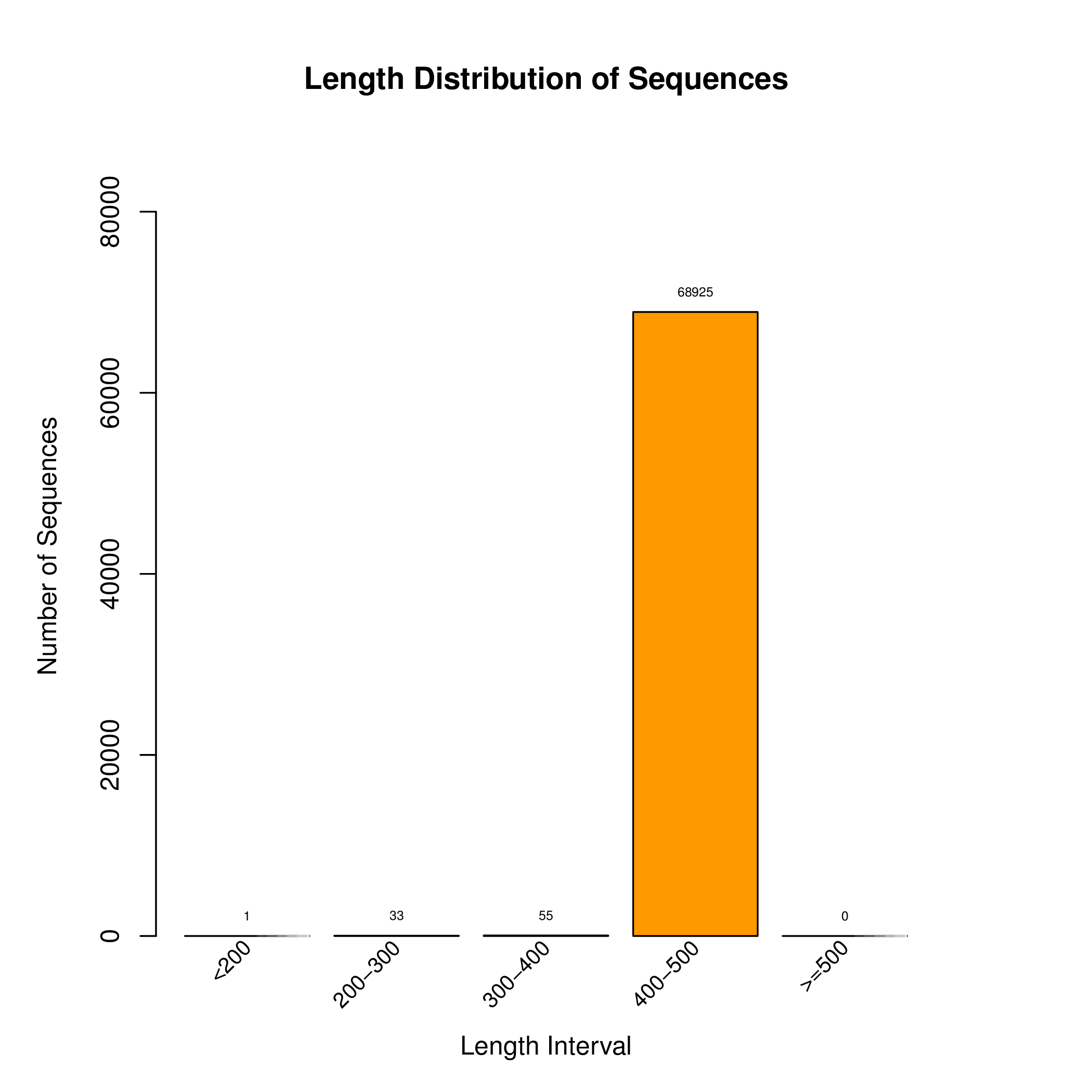

Supplement: Supplemental Information 1 [file peerj-11-16289-s001.zip › 2_clean_data/W9/W9_len_dist.png]

Venn diagram of W10\_vs\_W11\_vs\_W12\_vs\_W13

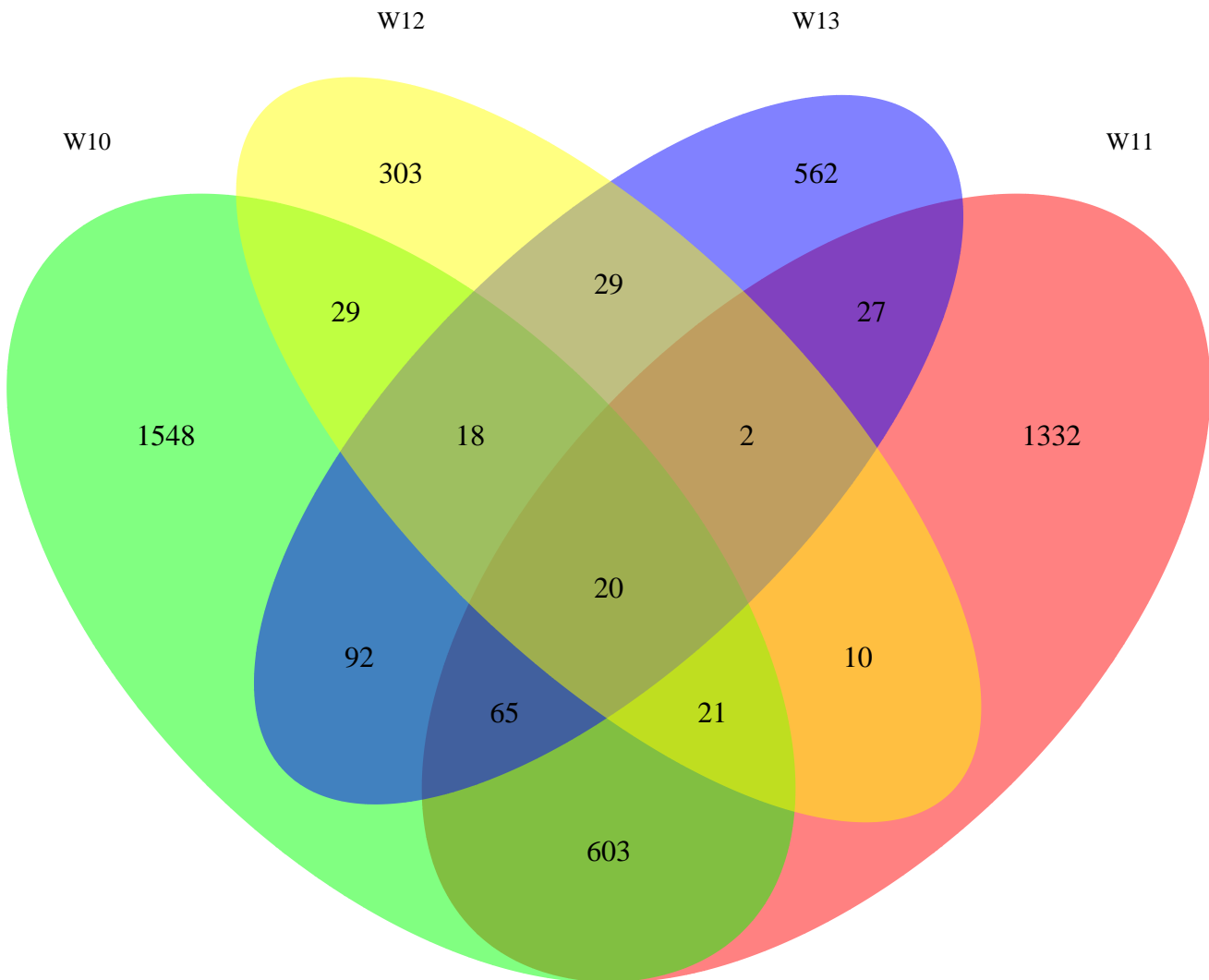

Supplement: Supplemental Information 1 [file peerj-11-16289-s001.zip › 3_ASV_profiling/2_venn_diagrams/venn_diagram_Group_W10_vs_W11_vs_W12_vs_W13.pdf]

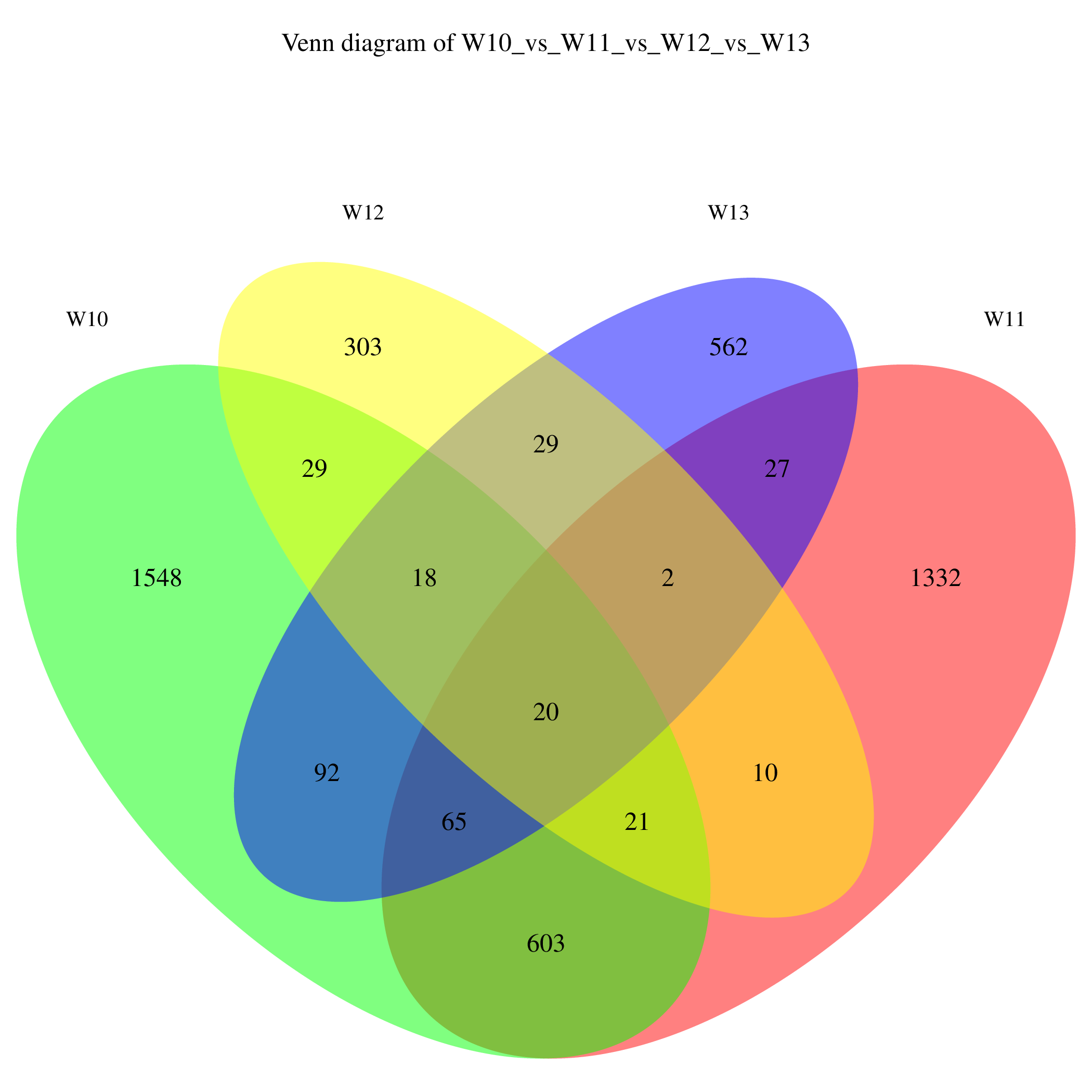

Supplement: Supplemental Information 1 [file peerj-11-16289-s001.zip › 3_ASV_profiling/2_venn_diagrams/venn_diagram_Group_W10_vs_W11_vs_W12_vs_W13.png]

Venn diagram of W1\_vs\_W2\_vs\_W3\_vs\_W13

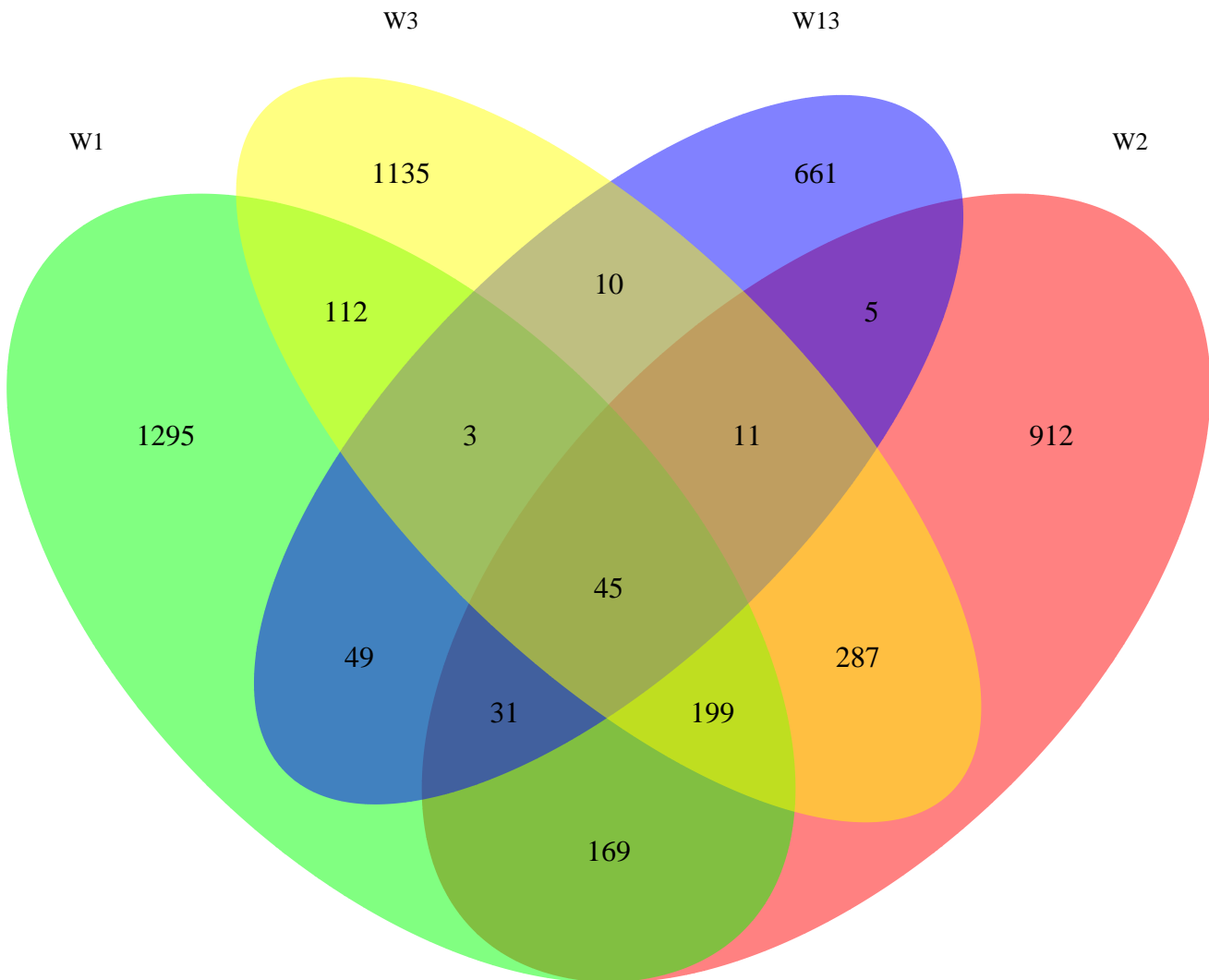

Supplement: Supplemental Information 1 [file peerj-11-16289-s001.zip › 3_ASV_profiling/2_venn_diagrams/venn_diagram_Group_W1_vs_W2_vs_W3_vs_W13.pdf]

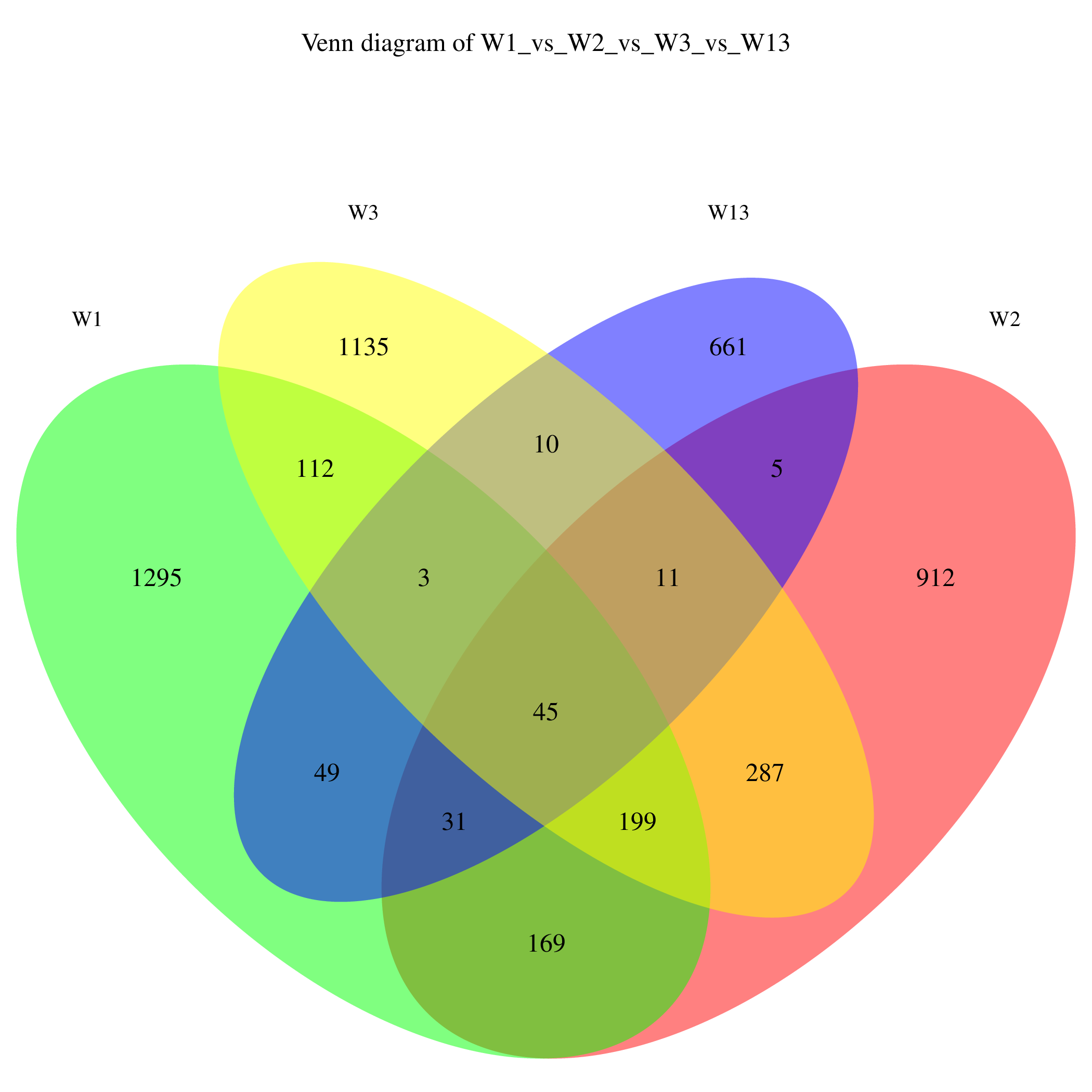

Supplement: Supplemental Information 1 [file peerj-11-16289-s001.zip › 3_ASV_profiling/2_venn_diagrams/venn_diagram_Group_W1_vs_W2_vs_W3_vs_W13.png]

Venn diagram of W4\_vs\_W5\_vs\_W6\_vs\_W13

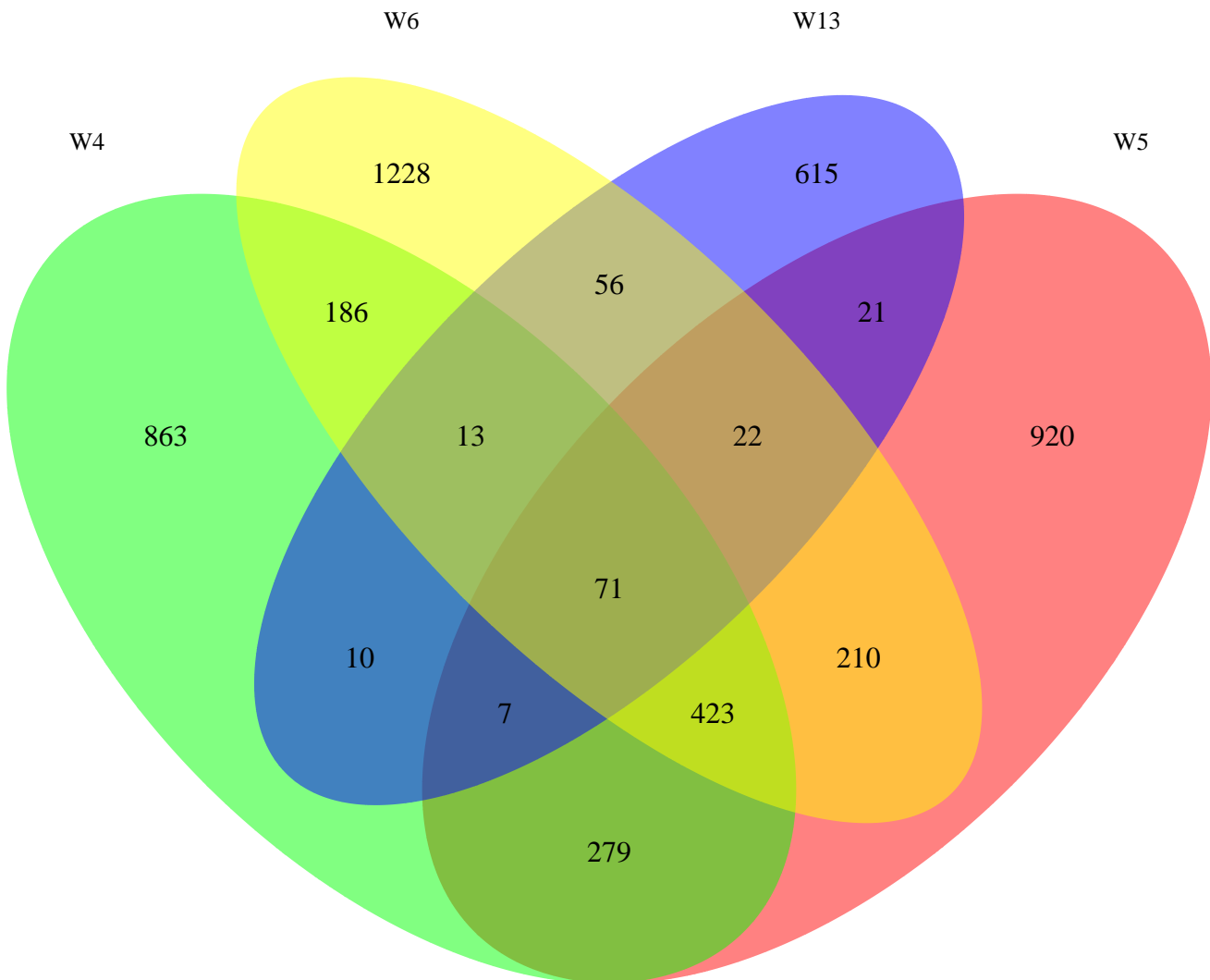

Supplement: Supplemental Information 1 [file peerj-11-16289-s001.zip › 3_ASV_profiling/2_venn_diagrams/venn_diagram_Group_W4_vs_W5_vs_W6_vs_W13.pdf]

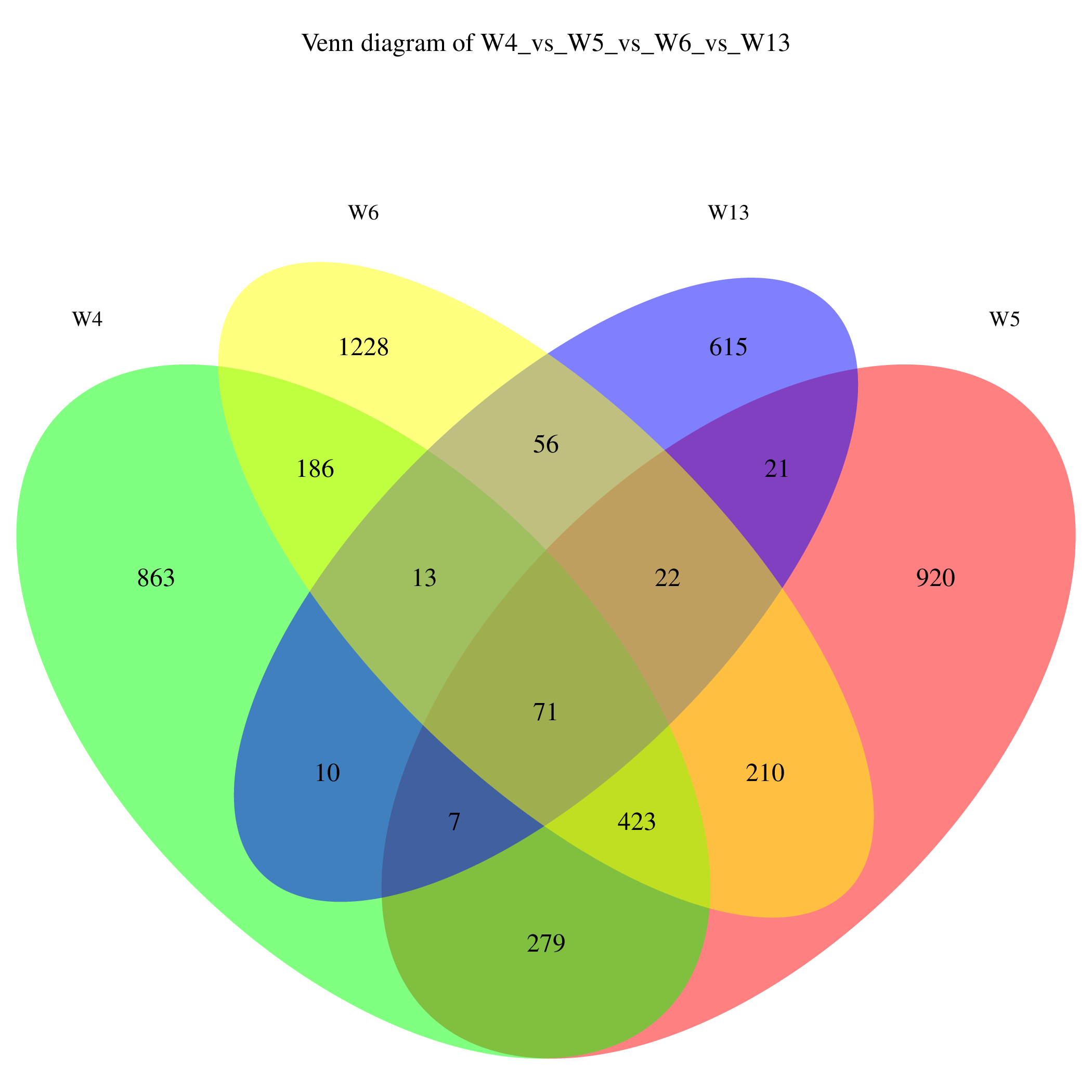

Supplement: Supplemental Information 1 [file peerj-11-16289-s001.zip › 3_ASV_profiling/2_venn_diagrams/venn_diagram_Group_W4_vs_W5_vs_W6_vs_W13.png]

Venn diagram of W7\_vs\_W8\_vs\_W9\_vs\_W13

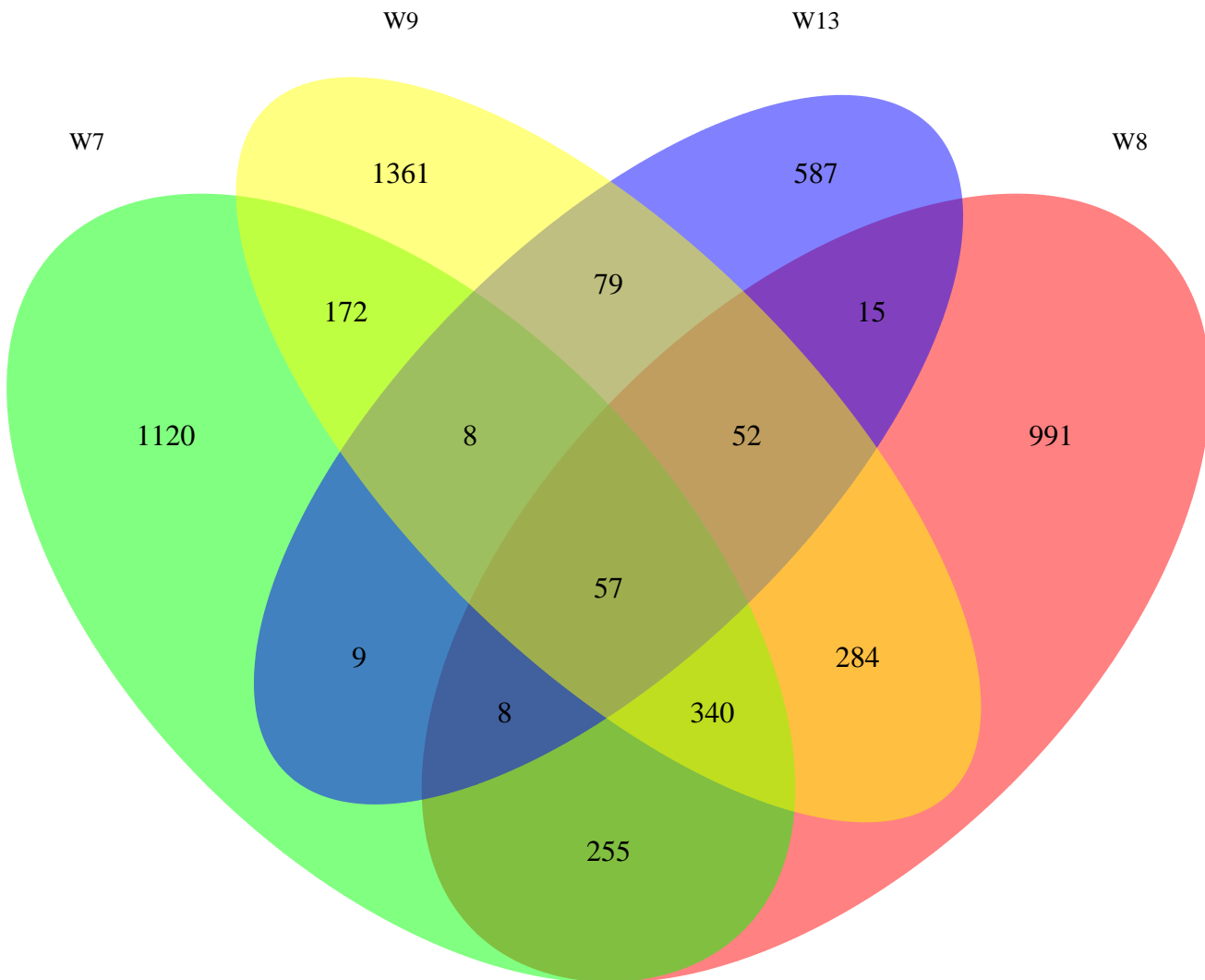

Supplement: Supplemental Information 1 [file peerj-11-16289-s001.zip › 3_ASV_profiling/2_venn_diagrams/venn_diagram_Group_W7_vs_W8_vs_W9_vs_W13.pdf]

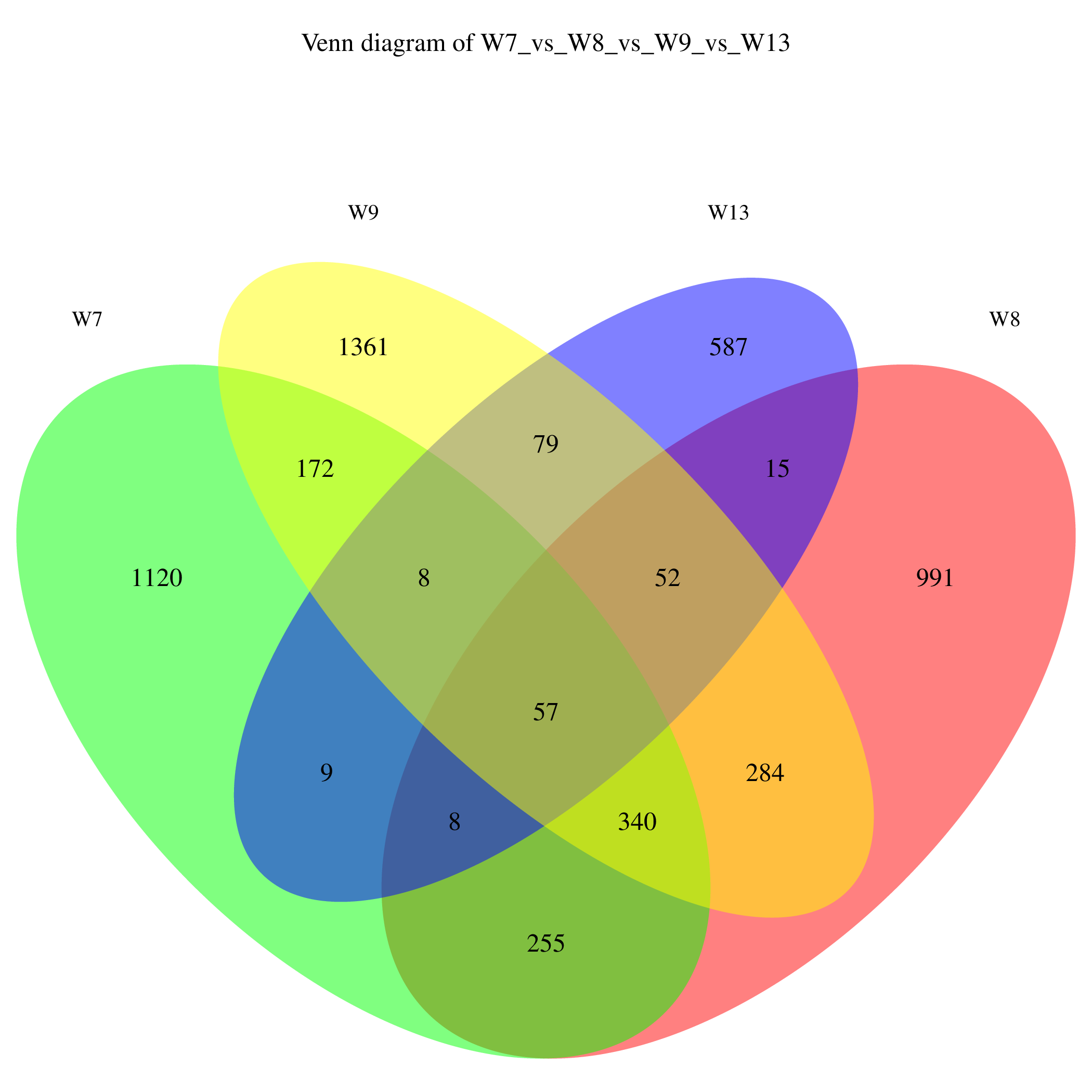

Supplement: Supplemental Information 1 [file peerj-11-16289-s001.zip › 3_ASV_profiling/2_venn_diagrams/venn_diagram_Group_W7_vs_W8_vs_W9_vs_W13.png]

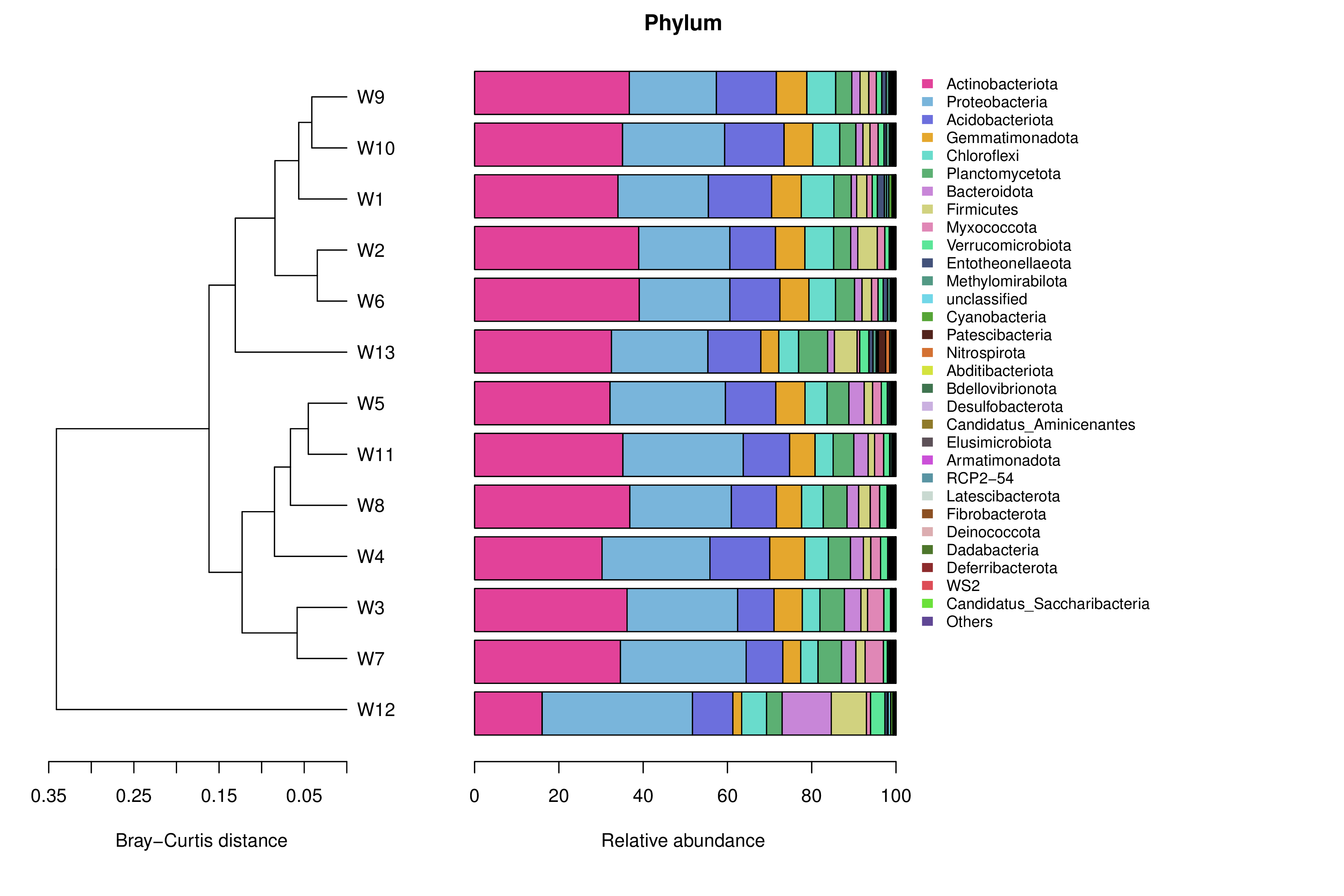

Supplement: Supplemental Information 1 [file peerj-11-16289-s001.zip › 6_taxonomy_community/2_Phylum/All/Phylum_abund_top30_cluster.png]

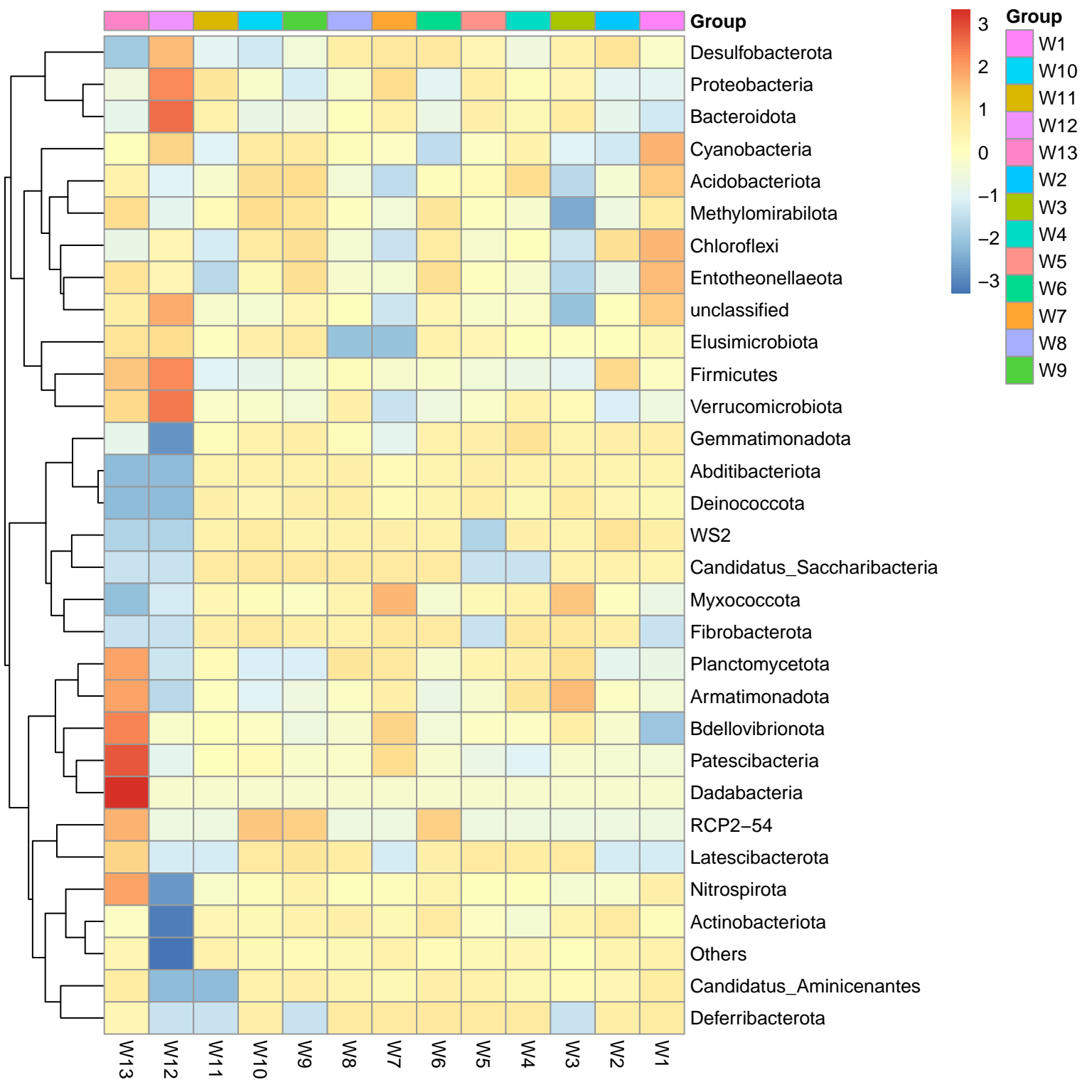

Supplement: Supplemental Information 1 [file peerj-11-16289-s001.zip › 6_taxonomy_community/2_Phylum/All/Phylum_abund_top30_heatmap.pdf]

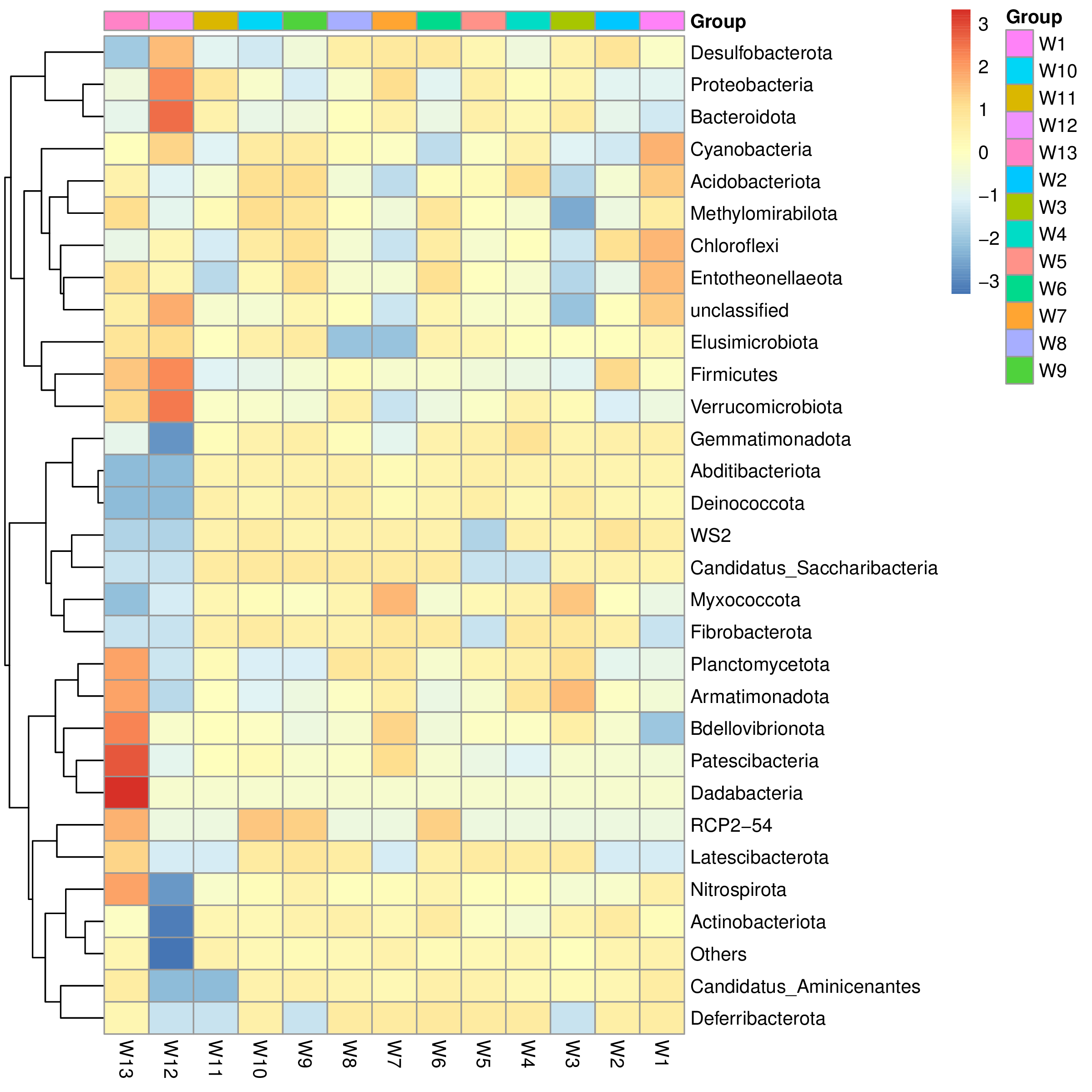

Supplement: Supplemental Information 1 [file peerj-11-16289-s001.zip › 6_taxonomy_community/2_Phylum/All/Phylum_abund_top30_heatmap.png]

# Phylum

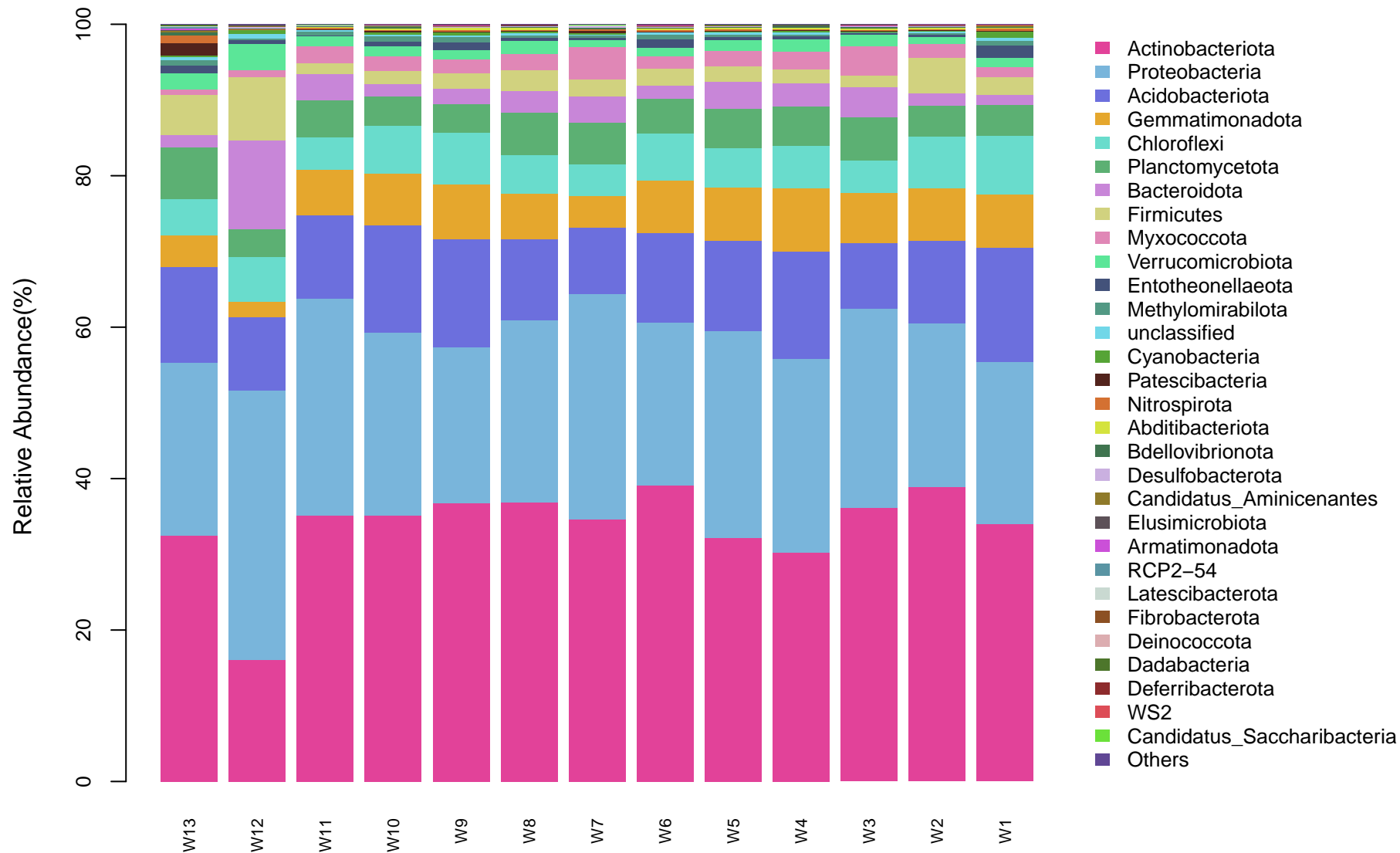

Supplement: Supplemental Information 1 [file peerj-11-16289-s001.zip › 6_taxonomy_community/2_Phylum/All/Phylum_abund_top30_stacked_bar.pdf]

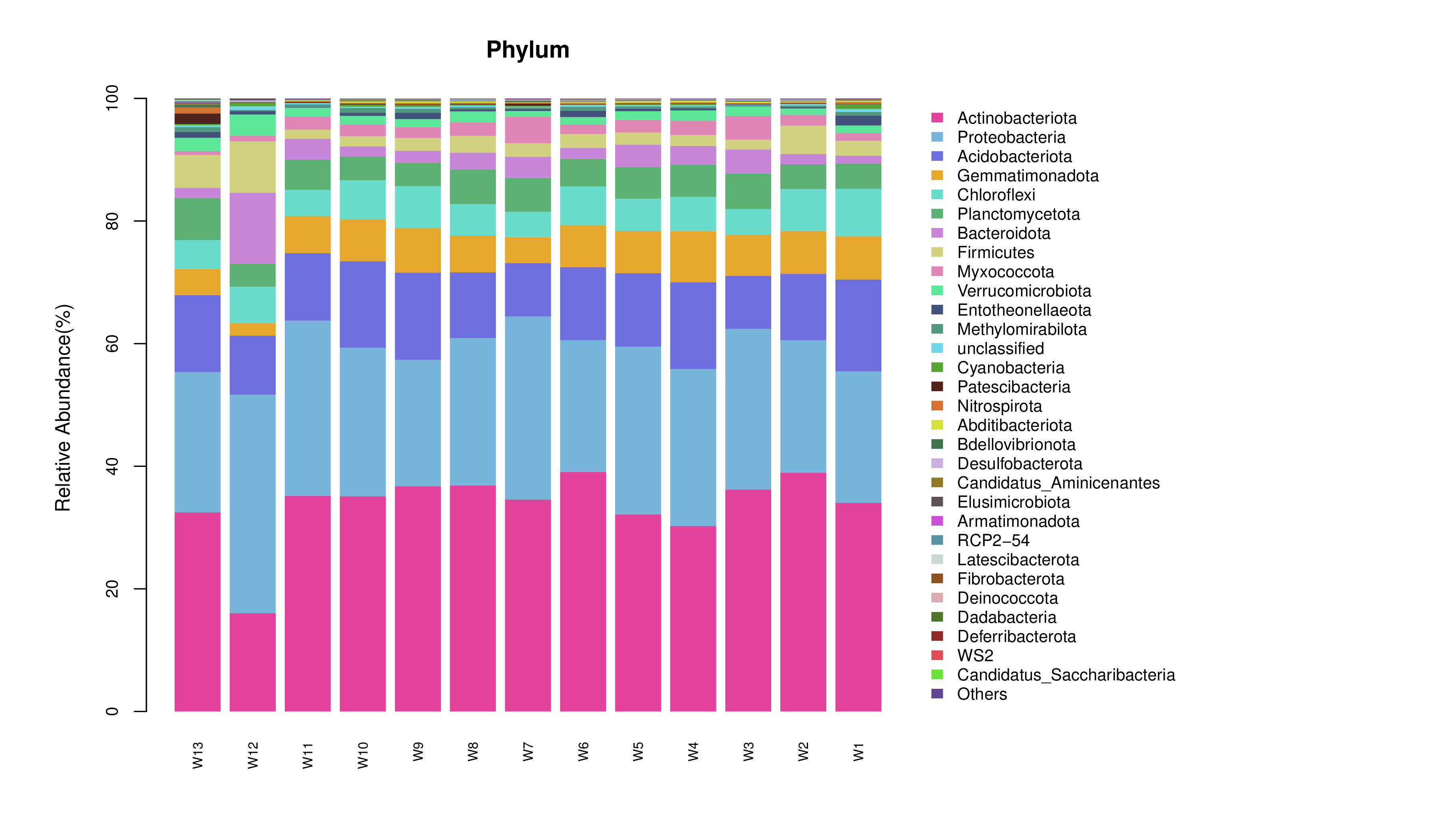

Supplement: Supplemental Information 1 [file peerj-11-16289-s001.zip › 6_taxonomy_community/2_Phylum/All/Phylum_abund_top30_stacked_bar.png]

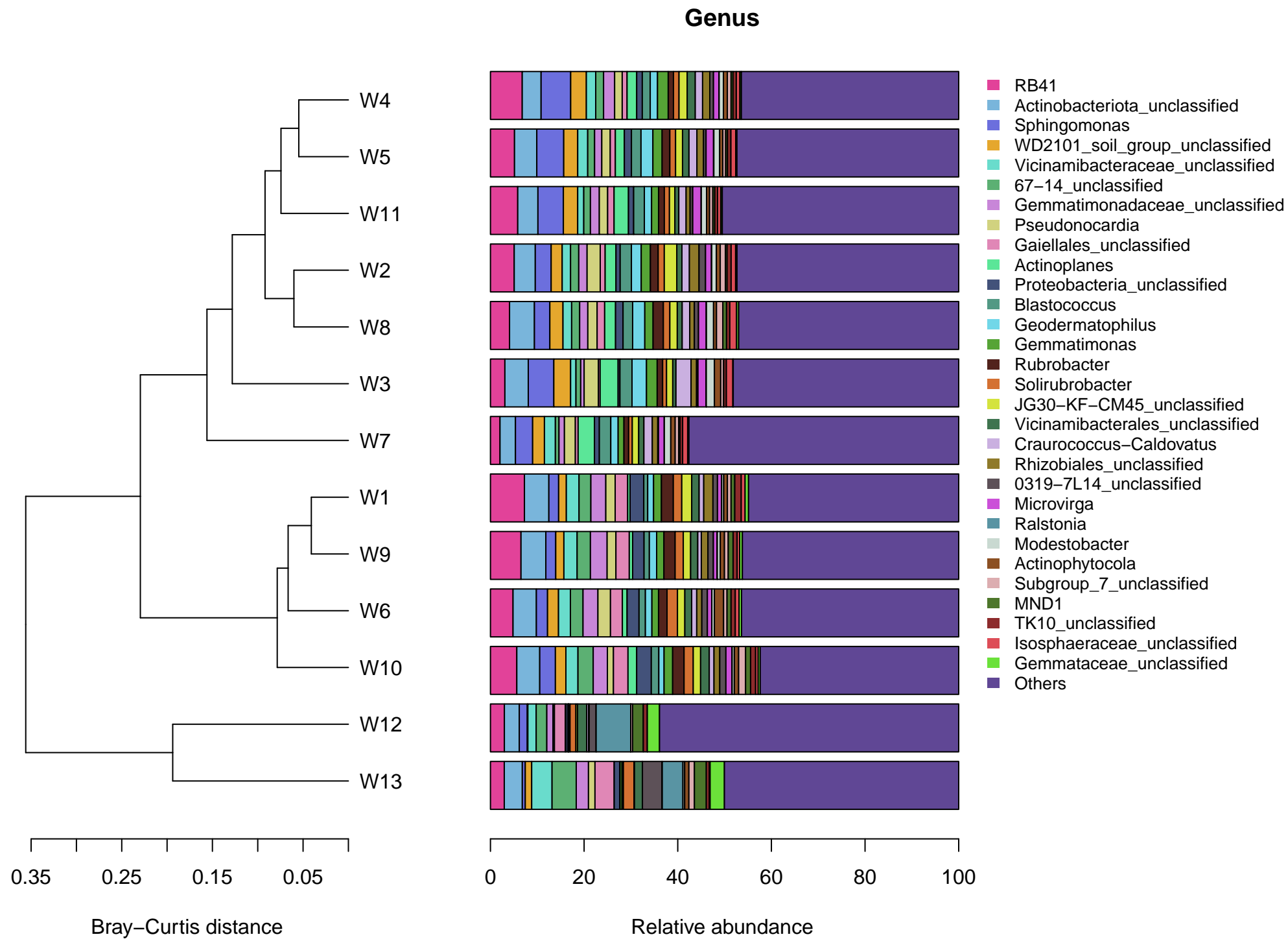

Supplement: Supplemental Information 1 [file peerj-11-16289-s001.zip › 6_taxonomy_community/6_Genus/All/Genus_abund_top30_cluster.pdf]

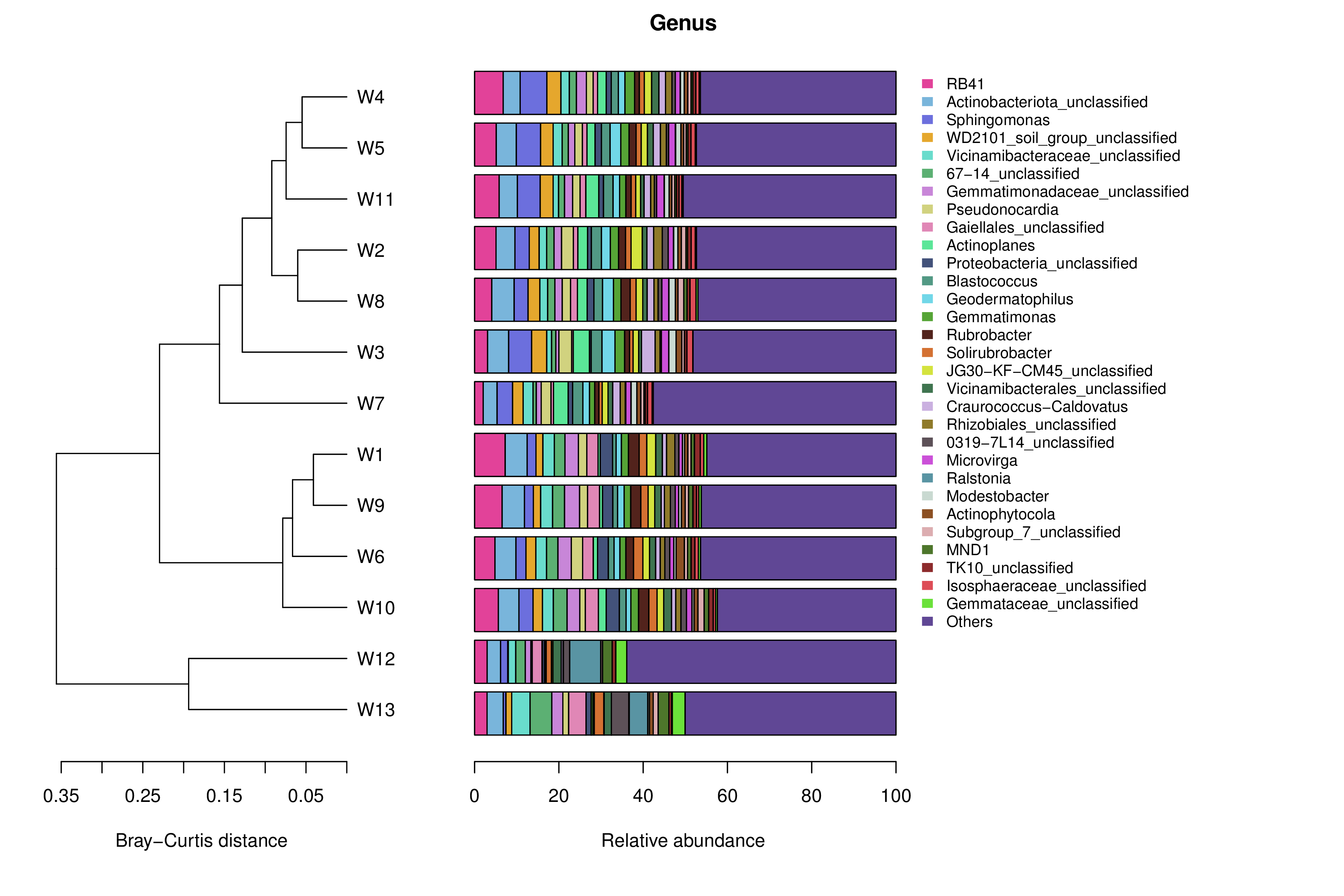

Supplement: Supplemental Information 1 [file peerj-11-16289-s001.zip › 6_taxonomy_community/6_Genus/All/Genus_abund_top30_cluster.png]

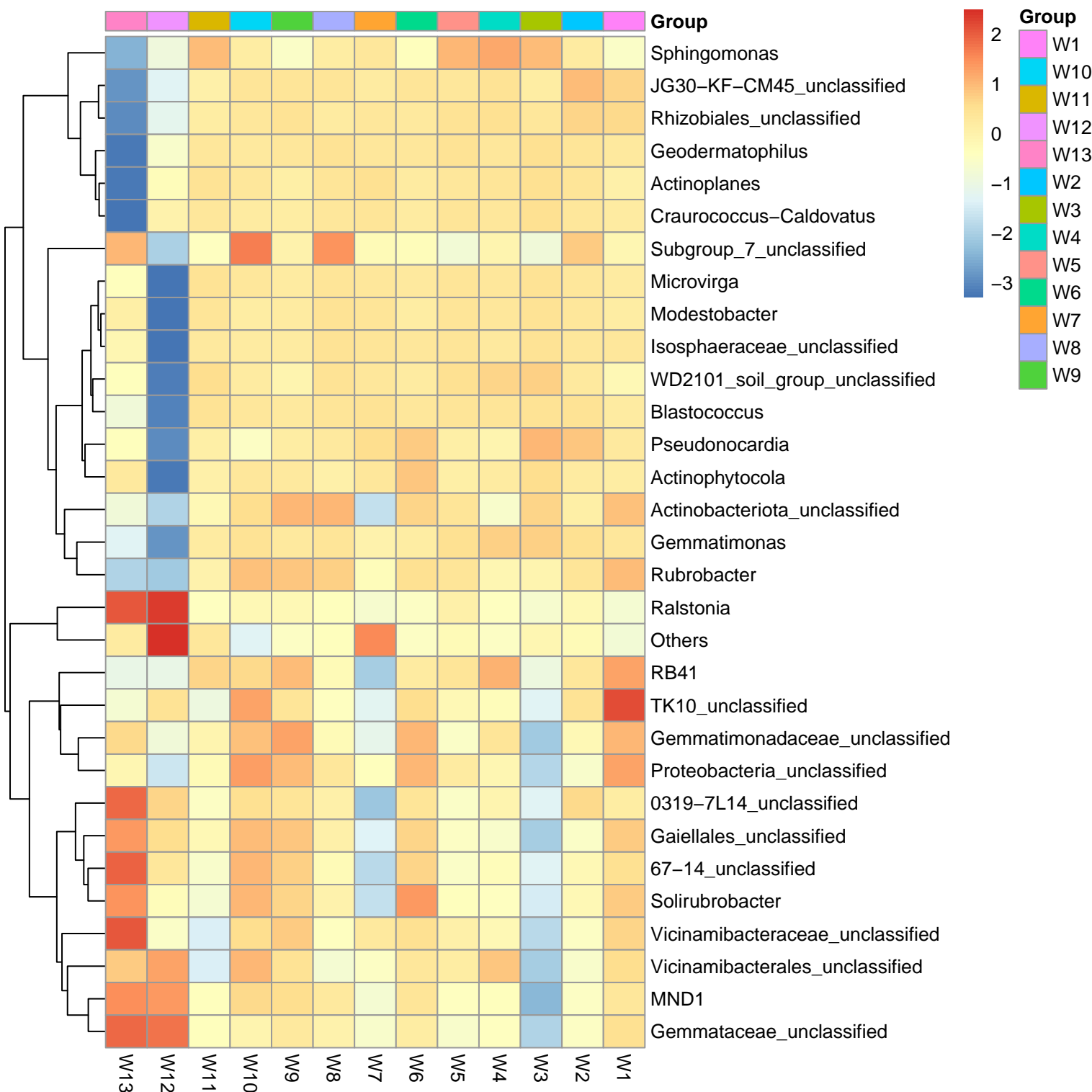

Supplement: Supplemental Information 1 [file peerj-11-16289-s001.zip › 6_taxonomy_community/6_Genus/All/Genus_abund_top30_heatmap.pdf]

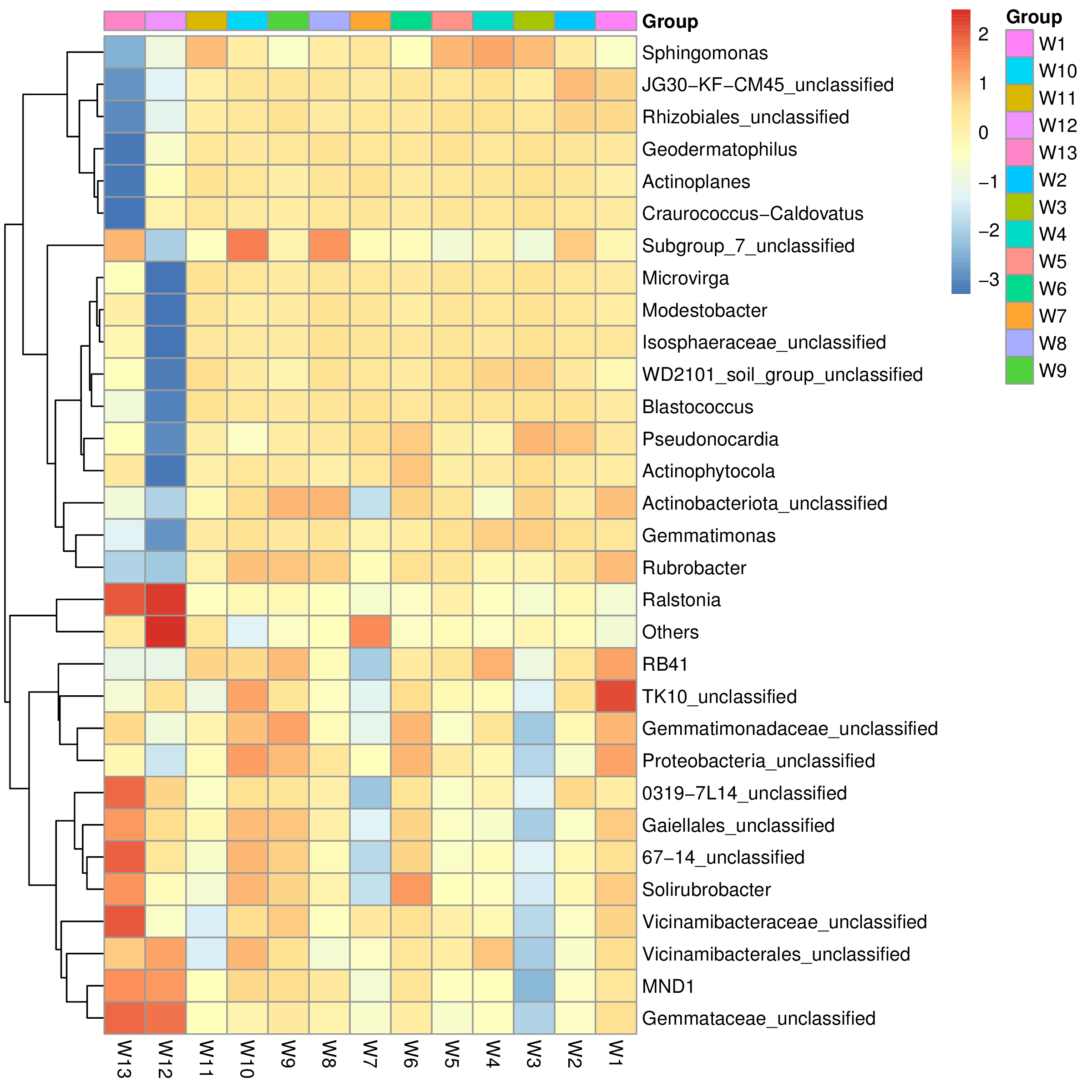

Supplement: Supplemental Information 1 [file peerj-11-16289-s001.zip › 6_taxonomy_community/6_Genus/All/Genus_abund_top30_heatmap.png]

# Genus

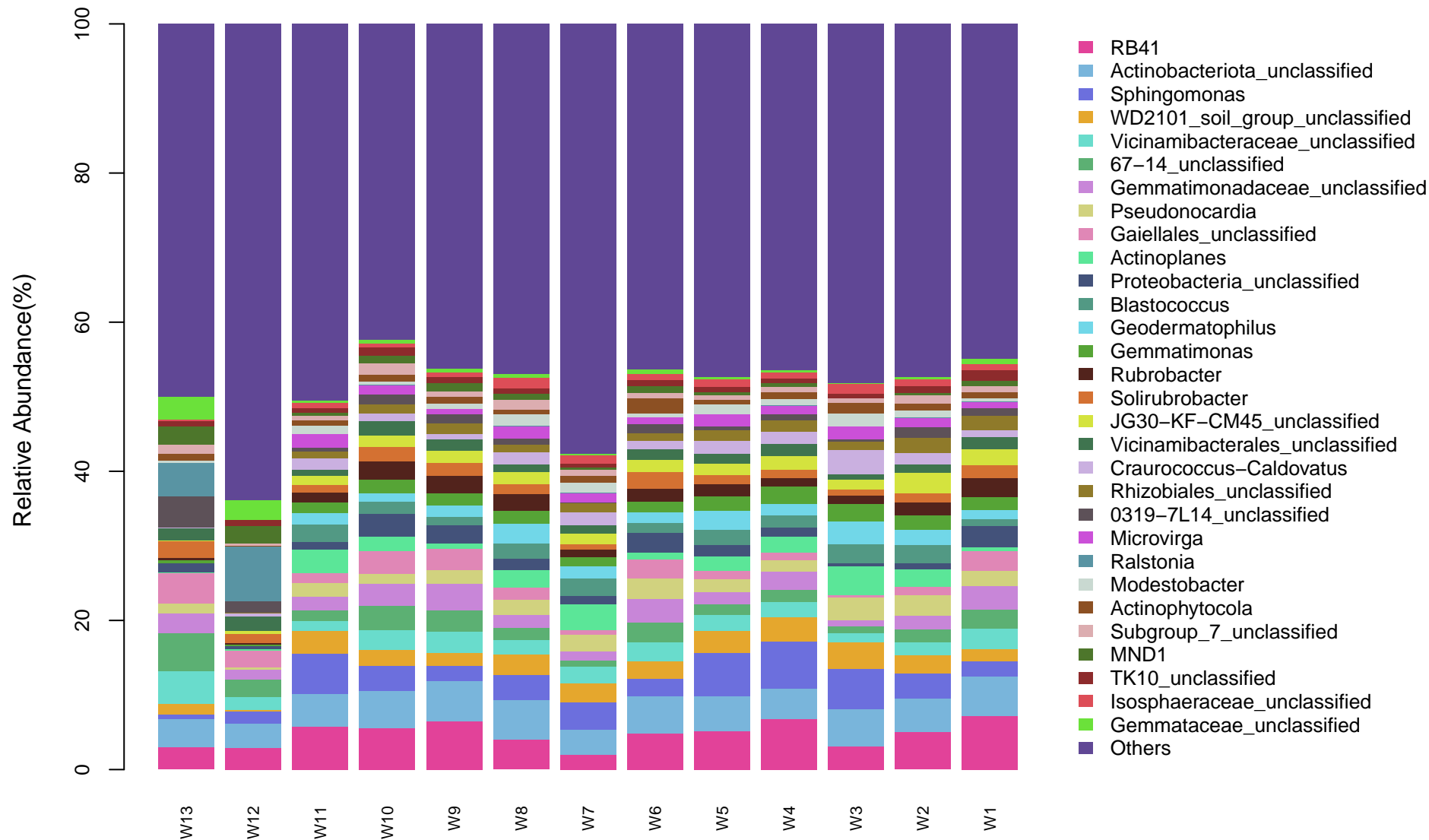

Supplement: Supplemental Information 1 [file peerj-11-16289-s001.zip › 6_taxonomy_community/6_Genus/All/Genus_abund_top30_stacked_bar.pdf]

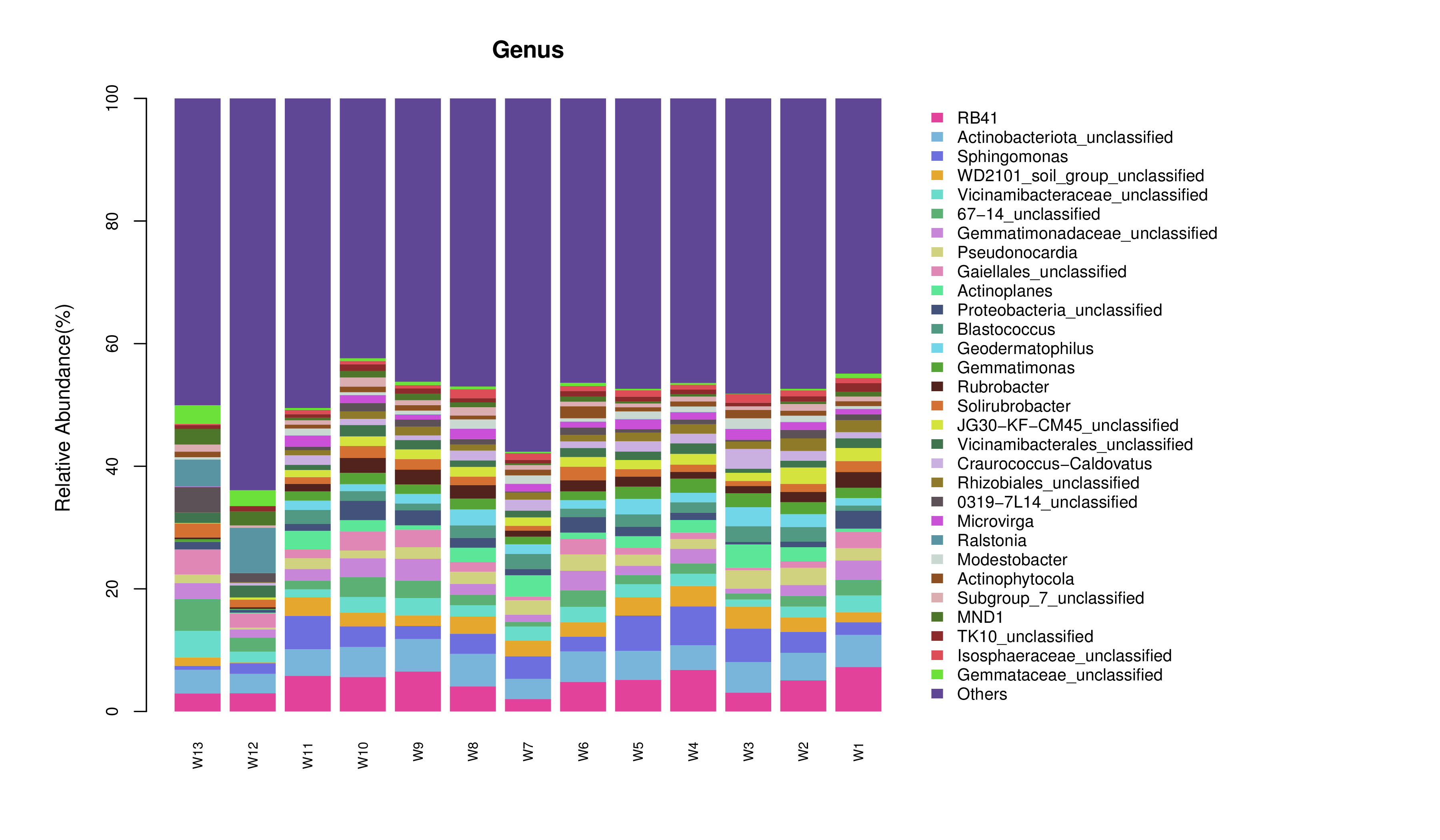

Supplement: Supplemental Information 1 [file peerj-11-16289-s001.zip › 6_taxonomy_community/6_Genus/All/Genus_abund_top30_stacked_bar.png]

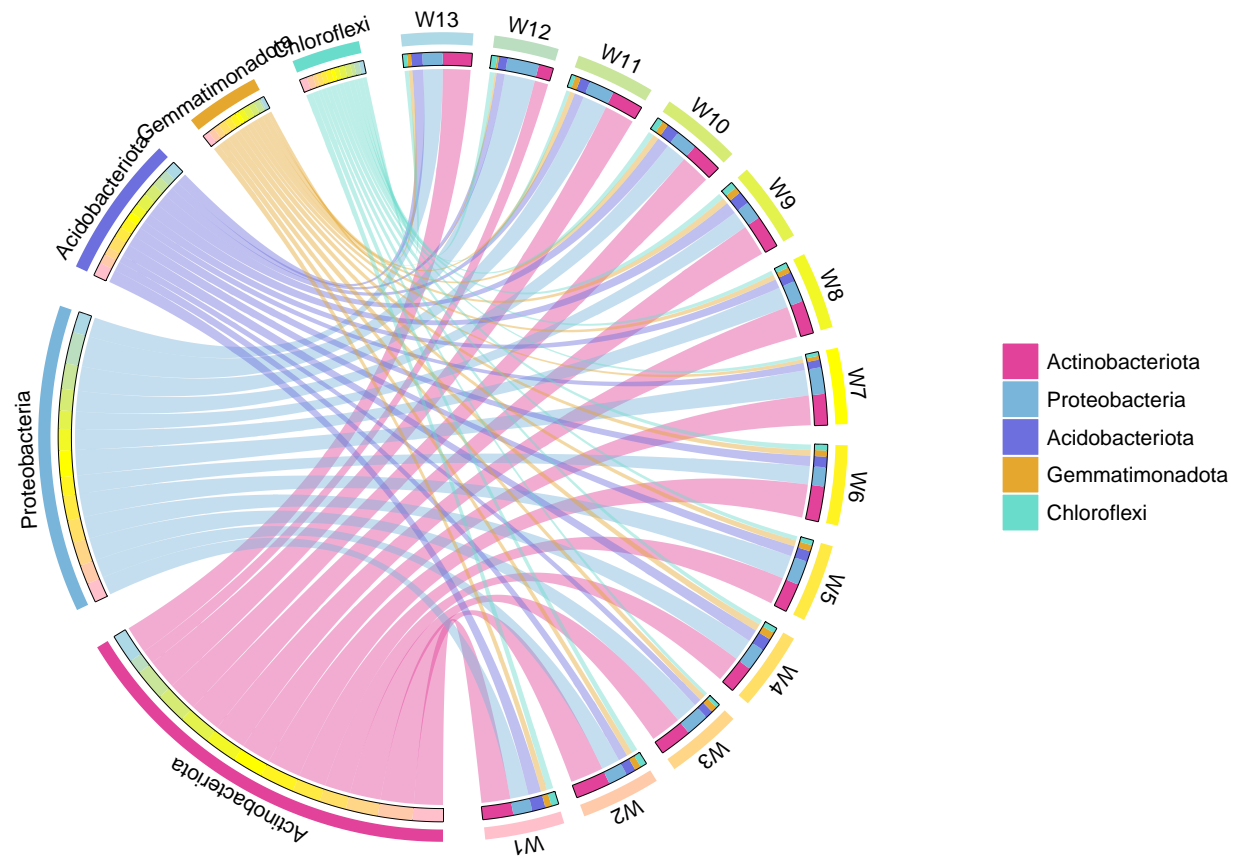

Supplement: Supplemental Information 1 [file peerj-11-16289-s001.zip › 8_advanced_analyse/1_taxonomy_analysis/2_Circos_plot/2_Phylum/Phylum_Circos_top5.pdf]

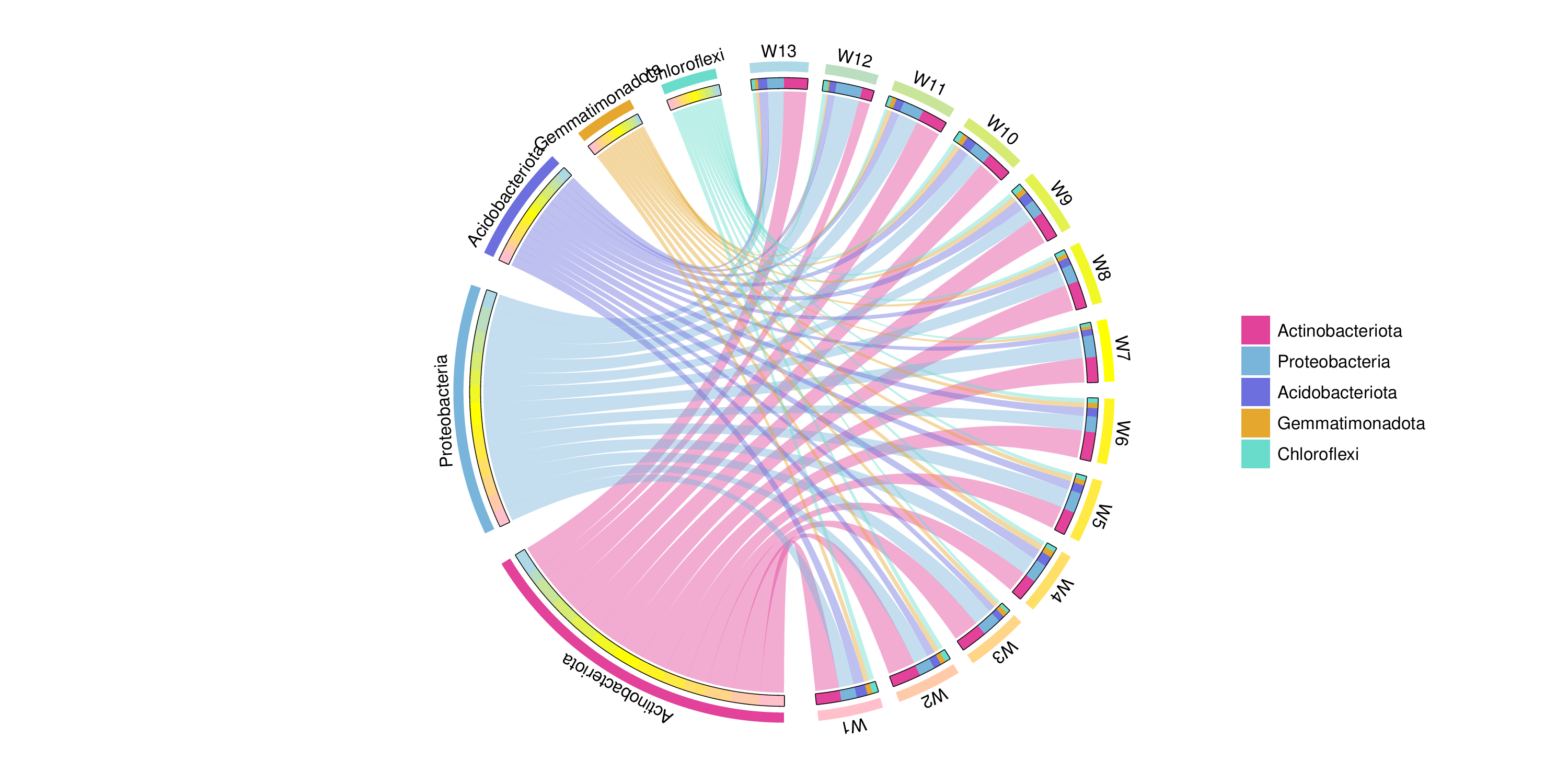

Supplement: Supplemental Information 1 [file peerj-11-16289-s001.zip › 8_advanced_analyse/1_taxonomy_analysis/2_Circos_plot/2_Phylum/Phylum_Circos_top5.png]

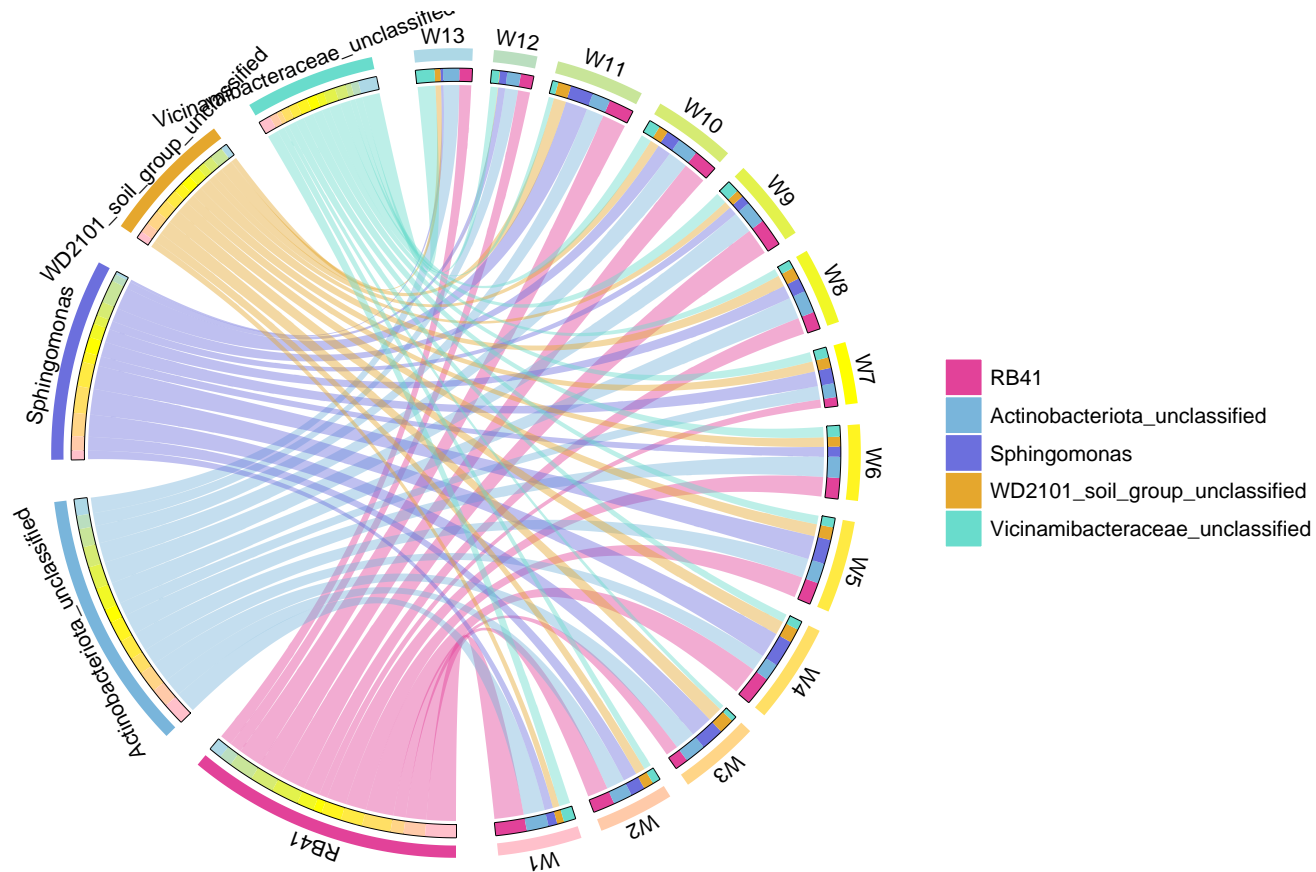

Supplement: Supplemental Information 1 [file peerj-11-16289-s001.zip › 8_advanced_analyse/1_taxonomy_analysis/2_Circos_plot/6_Genus/Genus_Circos_top5.pdf]

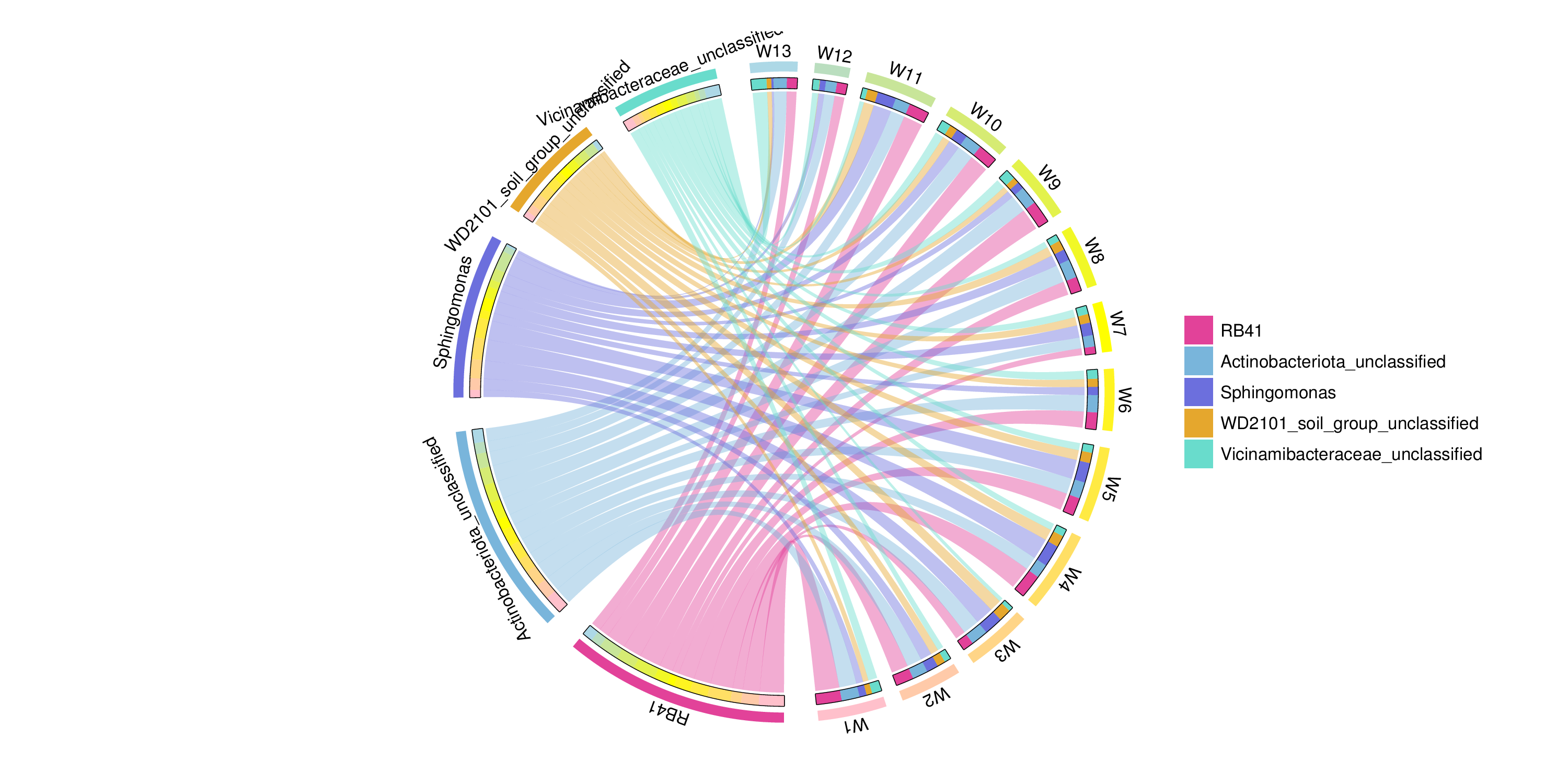

Supplement: Supplemental Information 1 [file peerj-11-16289-s001.zip › 8_advanced_analyse/1_taxonomy_analysis/2_Circos_plot/6_Genus/Genus_Circos_top5.png]

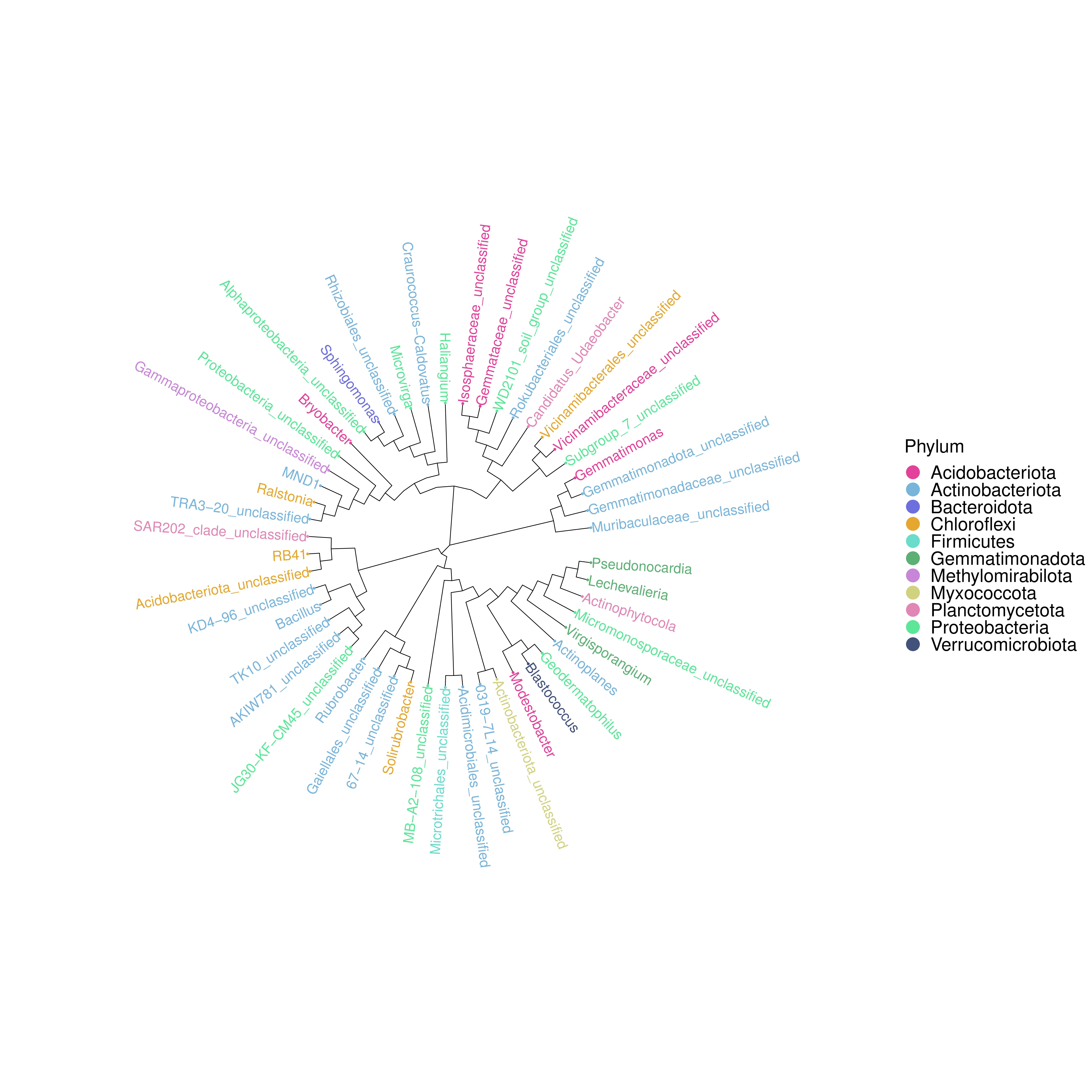

Supplement: Supplemental Information 1 [file peerj-11-16289-s001.zip › 8_advanced_analyse/1_taxonomy_analysis/4_Genus_phylotree/genus_phylotree.png]

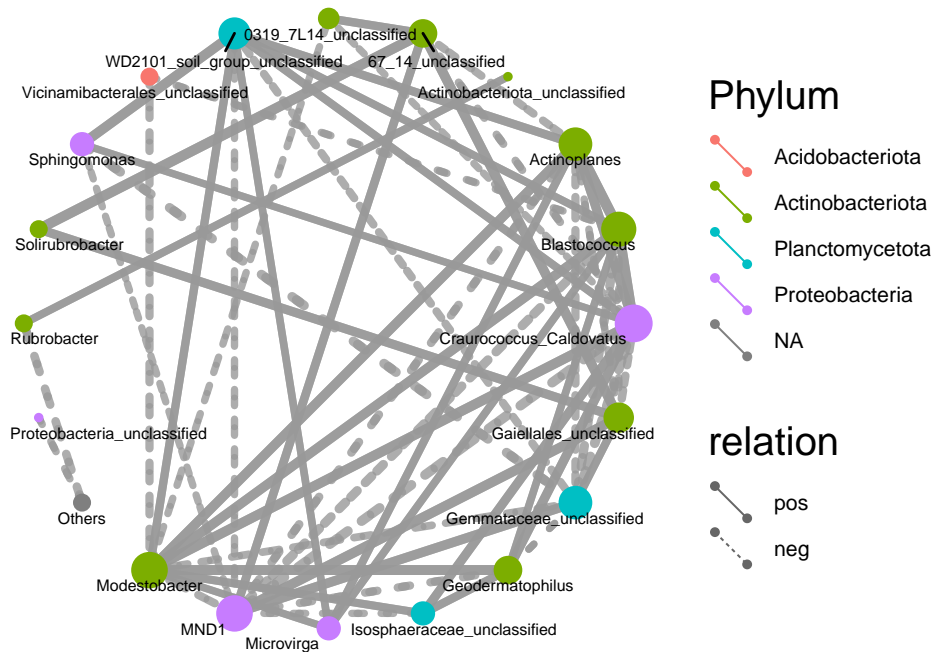

Supplement: Supplemental Information 1 [file peerj-11-16289-s001.zip › 8_advanced_analyse/3_correlation_analysis/network_0.8.pdf]

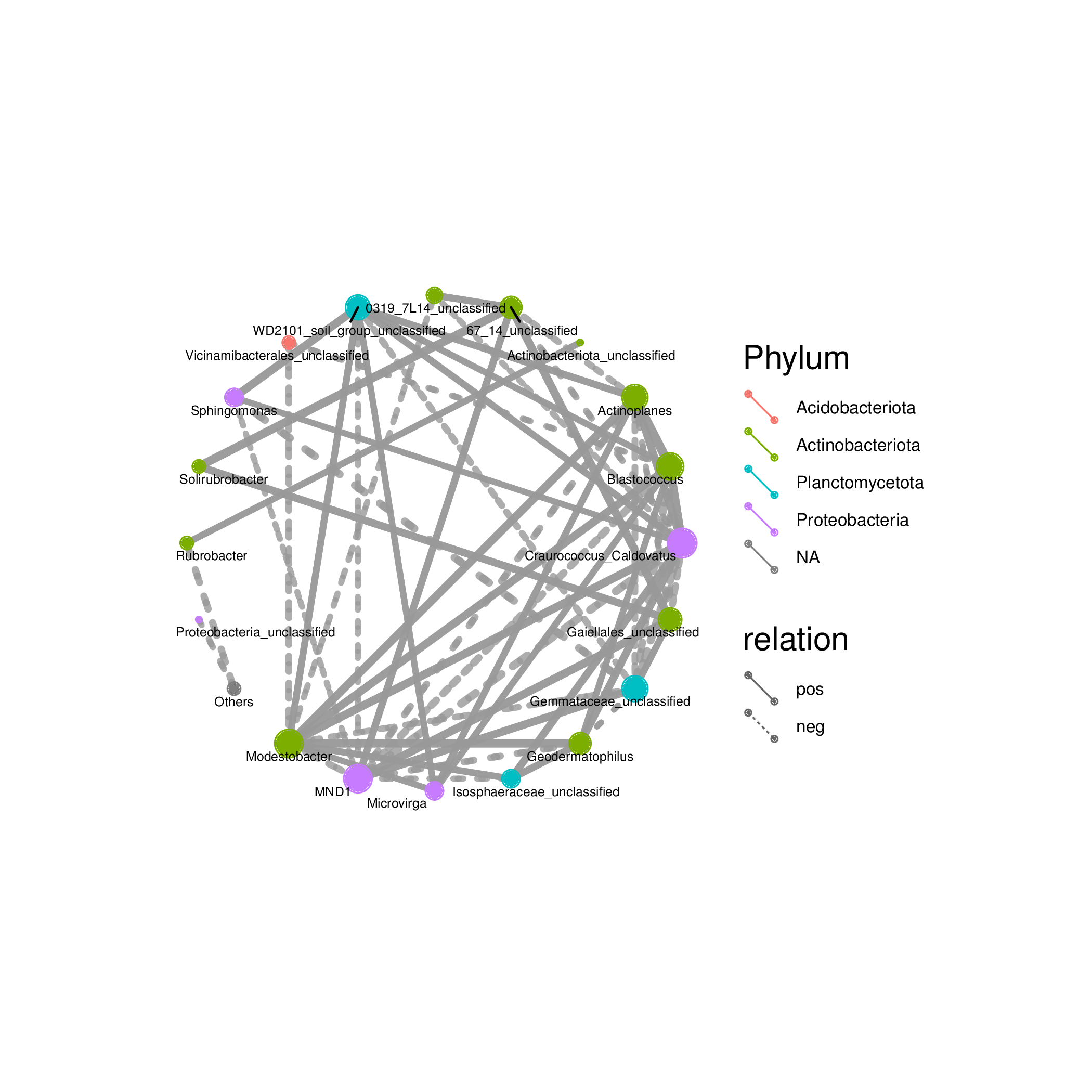

Supplement: Supplemental Information 1 [file peerj-11-16289-s001.zip › 8_advanced_analyse/3_correlation_analysis/network_0.8.png]
